# Different definitions of feeding intolerance and their associations with outcomes of critically ill adults receiving enteral nutrition: a systematic review and meta-analysis

**DOI:** 10.1186/s40560-023-00674-3

**Published:** 2023-07-05

**Authors:** Jianbo Li, Lijie Wang, Huan Zhang, Tongjuan Zou, Yan Kang, Wei He, Yuan Xu, Wanhong Yin

**Affiliations:** 1 Department of Critical Care Medicine, West China Hospital of Sichuan University, 37 Guo Xue Xiang St., Chengdu, 610041 Sichuan China; 2 Department of Critical Care Medicine, Beijing Tongren Hospital of Capital Medical University, Beijing, 100730 China; 3 Department of Critical Care Medicine, Beijing Tsinghua Chunggung Hospital, Tsinghua University, 168 Litang Rd., Beijing, 102218 China

**Keywords:** Feeding intolerance, Definitions, Critically ill adults, Enteral nutrition

## Abstract

**Background:**

A unified clinical definition of feeding intolerance (FI) is urged for better management of enteral nutrition (EN) in critically ill patients. We aimed to identify optimum clinical FI definitions based on reported evidence.

**Methods:**

We searched clinical studies comparing FI with non-FI with a clear definition, summarized the evidence by random-effect meta-analyses, and rated the certainty of evidence by the Grading of Recommendations Assessment, Development and Evaluation frameworks.

**Results:**

Five thousand five hundred twenty-five records were identified, of which 26 eligible studies enrolled 25,189 adult patients. Most patient-centered outcomes were associated with FI overall. Low to very low certainty evidence established FI defined as large gastric residual volume (GRV) ≥ 250 ± 50 mL combined with any other gastrointestinal symptoms (GIS) had a significant association with high mortalities in particular all-cause hospital mortality (odds ratio [OR] 1.90, 95% confidence interval [CI] 1.40–2.57), the incidence of pneumonia (OR 1.54, 95% CI 1.13–2.09) and prolonged length of hospital stay (mean difference 4.20, 95% CI 2.08–6.32), with a moderate hospital prevalence (41.49%, 95% CI 31.61–51.38%). 3-day enteral feeding (EF) delivered percentage < 80% had a moderate hospital prevalence (38.23%, 95% CI 24.88–51.58) but a marginally significant association with all-cause hospital mortality (OR 1.90, 95% CI 1.03–3.50).

**Conclusions:**

In critically ill adult patients receiving EN, the large-GRV-centered GIS to define FI seemed to be superior to 3-day EF-insufficiency in terms of both close associations with all-cause hospital mortality and acceptable hospital prevalence (Registered PROSPERO: CRD42022326273).

*Trial registration*: The protocol for this review and meta-analysis was registered with PROSPERO: CRD42022326273. Registered 10 May 2022.

**Supplementary Information:**

The online version contains supplementary material available at 10.1186/s40560-023-00674-3.

## Background

Feeding intolerance (FI) is one of the most concerned complications of enteral nutrition (EN). EN has been recommended as the first-line nutritional therapy to improve outcomes in critically ill patients who are unable to resume oral food intake by reducing stress-induced catabolic responses and preventing malnutrition associated with nutritional deficiencies or pre-existing malnutrition [1]. Monitoring FI during EN to revise the feeding plan timely is crucial to ensure successful feeding [2, 3]. However, there is still no consensus on the FI definition, resulting in differentiated practice and evidence interpretation as well as impeding the development and validation of interventions that might improve patient-centered outcomes [1, 4].

Numerous large-scale cohort studies [5–8] with evidence-based data on FI in critical care medicine are propelling the formulation of a unified clinical definition for better management of EN in critically ill patients. In 2012, recommendations of the European Society of Intensive Care Medicine (ESICM) working group on abdominal problems proposed for the first time that “feeding intolerance is present if at least 20 kcal/kg BW/day via enteral route cannot be reached within 72 h of feeding attempt” [2]. However, the definition based on the 3-day enteral feeding (EF) insufficiency [approximately < 80% of standard feeding delivered (25 kcal/kg BW/day)] is questioned as inconducive to early/real-time FI judgment and subsequent processing. Therefore, a practical definition framework with immediate objective indicators that brings forward the time window of FI diagnosis is needed during EN.

Gastric residual volume (GRV) potentially with objectivity and quantification once preferred widely as one of the gastrointestinal symptoms (GIS) to define FI with or without other GIS. However, studies showed concerns about the reliability of GRV monitoring: unreliable prediction for regurgitation and aspiration, undefined normal value, no validated single cutoff value, and no standard techniques for measuring [9, 10]. A recent comprehensive systematic review encompassing 89 empirical studies concluded that the GRV threshold had no significant correlation with the prevalence of FI due to poor relationships with gastric emptying [11]. However, this conclusion mainly based on empirical evidence was worth discussing, and as this review’s author pointed out that FI was reportedly common among critically ill adults but inconsistently defined in the literature [11]. Accordingly, a consensus for the definition of FI is strongly expected for future research and is of substantial clinical significance.

To reconceptualize FI, we proposed to position the clinical value of various FI definitions based on the correlation of patient-centered outcomes, including but not limited to mortality, pneumonia, length of hospital stay, etc.—an extended approach stemmed from Reintam-Blaser et al.’s study [5] that investigated a series of self-defined FIs and their relevance with mortality in the same cohort. Here, we undertook a systematic review and meta-analysis to assess the associations of FI across different definitions with patient-centered outcomes, together with their prevalence, providing a comprehensive reference basis for the consensus process of formulating FI definition, for the better management of EN in critically ill adults.

## Methods

### Study design

This systematic review and meta-analysis were performed as part of the China adult ICU nutritional assessment and monitoring guideline project. A nationwide multidisciplinary panel consisting of intensive care physicians, emergency physicians, general surgeons, nutritionists, nurses, and methodologists formulated the clinical questions and provided input into the study protocol. They sought evaluation of FI in critically ill adult patients receiving EN. The results will inform the group’s guideline recommendation. We registered our study on PROSPERO (CRD42022326273, registered 10 May 2022.) and followed the Meta-analysis of Observational Studies in Epidemiology (MOOSE) and Preferred Reporting Items for Systematic Reviews and Meta-Analyses (PRISMA) [12, 13] (Additional file 1: Table S1).

### Search strategy and selection criteria

We searched PubMed, Web of Science, the Cochrane Library, ClinicalTrials.gov, and main Chinese medical databases (CBM, CNKI, and WanFang) from inception to April 26, 2022. To supplement the identified citations, we searched the citation lists of key reviews and meta-analyses. Searches included terms relating to EN, FI, and critical illness and were completed by two appointed authors independently according to the same proposed strategies (Additional file 2: Table S2). A re-run search was done before the completion of the manuscript to include the latest eligible studies during writing. Duplicate records were removed with EndNote X9 (Clarivate Analytics, Philadelphia, PA, USA). Teams of paired reviewers independently screened titles, abstracts, and then full-text manuscripts, and extracted data on study conductors, study design and settings, participant baseline characteristics, exposures, and outcomes. Discrepancies were resolved by discussion or, if necessary, by the panel of experts adjudication.

Eligible studies involved critical illness adults receiving EN regardless of the study design (a cohort, a case–control, or an RCT). These studies compared FI with non-FI of which they have a clear definition description (such as a large GRV with a clear threshold to define FI) to distinguish between exposure and non-exposure; reported the patient-centered outcomes comprising all-cause mortality/ICU mortality (usually with a time frame of fewer than 30 days)/hospital mortality (with a time frame of 30–90 days)/long-term mortality (with a time frame of 90 days or more), the incidence of pneumonia (given the existing literature, we did not mandate a specific definition for the presence of FI related pneumonia, nor provided a generally accepted definition of ventilator-associated pneumonia), length of hospital/ICU stay, or mechanical ventilation days and had an EN duration of 4 h or more (chosen as the shortest duration in which FI, If present, are more likely clinically determined) with no limit set on maximum duration. We excluded studies for these main reasons: with FI only as a general complication or a baseline characteristic or other reasons resulting in inability to directly present the association of FI with outcomes; with unacceptable poor reporting quality (referred to the panel of experts for consideration of their exclusion according to the STROBE and the CONSORT statements [14, 15]); with a cohort of more than 75% overlap with the already included eligible studies but no more usable data for pooling. PRISMA 2020 flow diagram was produced using the R package “PRISMA2020” [16].

### Data analysis

The guideline panel judged the following outcomes as primary outcome indicators: all-cause hospital mortality, all-cause long-term mortality, the incidence of pneumonia, length of hospital stay, and mechanical ventilation days. The panel judged the following as secondary outcomes: all-cause mortality, all-cause ICU mortality, and length of ICU stay. They adjusted the importance level according to the definition details of specific FI which would markedly affect the outcome (Additional file 3: Table S3). Outcome data were extracted preferentially from results produced by cohorts comparison with balanced baseline characteristics if multiple dependent sub-cohorts were present to prove the same outcome. Alternatively, we extracted results from cohorts with larger sample sizes or a longer follow-up. If present, the results of multiple independent cohorts in a study to demonstrate the same outcome would enter the pooling program as they were. We chose the following measures of effect: odds ratios (ORs) for individual-based binary outcomes, such as all-cause hospital mortality; incidence rate ratios for event-based binary outcomes, such as pneumonia events in which people can have more than one event; mean differences (MDs) for comparison in the length of hospital stay, length of ICU stay and mechanical ventilation days. When studies did not report the sample mean and standard deviation but instead provided quantiles, we converted these data using a Box–Cox method [17] with the R package “estmeansd”.

Meta-analysis was performed with a random-effect model by R package “meta”. The pooling estimator was based on the Mantel–Haenszel method for binary outcome data or the inverse-variance method for continuous outcome data. For studies with a zero cell count, 0.5 was added to all cell frequencies of these studies. The percentage of variability in the effect sizes was evaluated by Higgin’s and Thompson’s I^2^, according to which heterogeneity was divided into low (less than 25%), moderate (25–75%), and substantial (more than 75%) [18]. Heterogeneity from the between-study variance was measured with tau-squared based on the restricted maximum-likelihood method [19]. A two-tailed *P* value less than 0.05 was statistically significant.

Forest plots and league tables of the relative effects were used to visualize comparisons of outcomes estimations across FI definitions. We performed prespecified subgroup analyses for all outcomes according to FI definition-related details including thresholds for large GRV (75 ± 50 mL vs 250 ± 50 mL vs 500 ± 50 mL vs 1000 mL), and GRV measured intervals (4 h vs 6 h vs 24 h) in a specific sub-population or overall population disregarding any kind of FI definitions across population characteristic levels including the proportion of male patients, average age, the proportion of underlying diseases (surgical, abdominal surgery, trauma, burn, digestive, sepsis), proportion of mechanical ventilation, average Sequential Organ Failure Assessment (SOFA) score, and Acute Physiology and Chronic Health Evaluation II (APACHE II) score (Additional file 4: Fig S1). In the post hoc subgroup analysis, we limited the total number of candidate GIS to 4 vs 5 for the impact of the number of GIS to determine FI on outcomes. In sensitivity analysis, we removed the studies written in Chinese. The publication bias assessments for outcomes in the overall population are detailed in Additional file 5 and 6 (Table S4 and Fig S2). Two researchers (LW and HZ) independently assessed the risk of bias for every outcome indicator in each included study or FI-definition-differentiated cohort using the Newcastle–Ottawa Scale (NOS) [20], with discrepancies resolved by a third researcher (JL). The NOS scores were assessed for (1) selection, (2) comparability, and (3) exposure for case control or outcome for cohort studies. Each domain is composed of 2 to 4 items of criteria, and each criterion was scored in the form of stars. As described in our previous report [21], a total score of 8 or 9 was assessed as low risk of bias, 6 or 7 as some concerns, and ≤ 5 as high risk (Additional file 7: Table S5).

According to the Grading of Recommendations Assessment, Development, and Evaluation (GRADE) [22], the certainty of the evidence of each comparison across FI definitions was rated as high, moderate, low, or very low. Absolute effects of the FI were calculated with non-FI risk (as baseline risk) and the pooled relative effects were compared with non-FI across different FI definitions in which effects of FI and non-FI changed pairwisely. When a mean NOS score was no more than 6, we considered rating down for risk of bias. When evidence body occurred with high heterogeneity and obvious directional differences between studies, we considered rating down for inconsistency. To make judgments regarding rating down for imprecision, we chose targets of GRADE certainty of evidence ratings with a small effect threshold method which is also called a minimally important difference (MID) [23, 24]. For this estimate, we posited that the MIDs for FI were 1 ± 0.2 times the adverse risk compared with non-FI, or 0 ± 2 days for duration variables. Therefore, the evidence body was considered as serious imprecision when it came with marginal significant results judged by overlapping a small effect threshold with 0.8 or 1.2 times the death/pneumonia risk, or 2 fewer or 2 more days rather than the usual effect thresholds of 1 times risk or 0 days. In other considerations of the GRADE framework, publication bias was strongly suspected for the number of studies less than 10 and all plausible residual confounding was considered none (Additional file 3: Table S3).

### Role of the funding source

The funder had no role in the study design, data collection, analysis, interpretation, or writing of the manuscript and the decision to submit it. The corresponding author had full access to all the data in the study and had final responsibility for the decision to submit it for publication.

## Results

### Overview of included studies, overall results, and quality evaluation

**Fig. 1:**
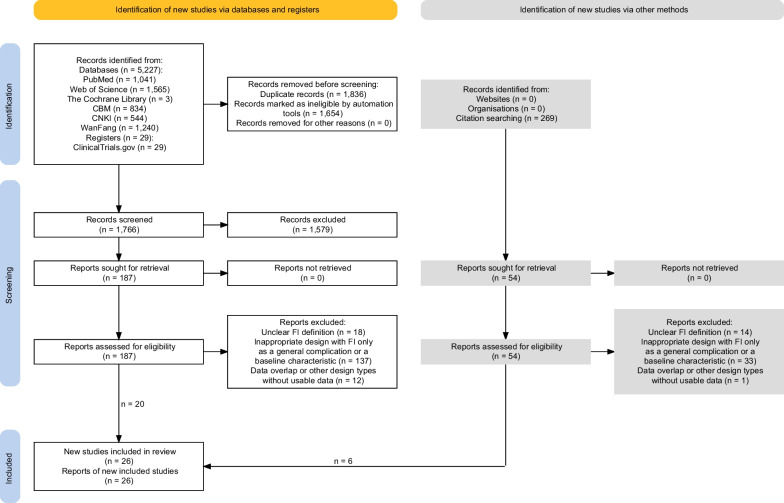
PRISMA flow diagram. New studies were identified via databases and registers together with other methods for reporting of grey literature searches and results. The databases of CBM, CNKI, and WanFang are the main Chinese medical databases. *FI* feeding intolerance

**Fig. 2:**
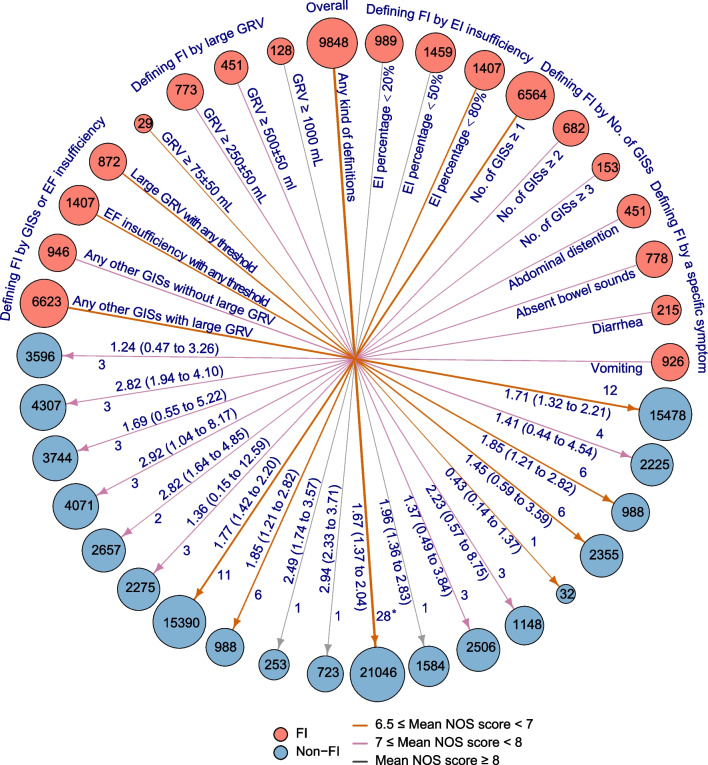
General summary plots. The number of pooled studies together with involved patients in two arms as well as ORs for all-cause mortality overall and by different FI definitions is summarized. Each node (solid circle) together with the number in it stands for the number of patients with FI (filled with orange) or non-FI (filled with blue) under a certain definition or overall situation with definition indiscriminately. The size of nodes is logarithmically proportional to the number of patients (i.e., the sample size in the FI or non-FI arm) involving the specific classification situation. The color of the arrows stands for different mean NOS score levels and the number right to the arrows as well as arrow thickness stands for the number of independent cohorts involving data pooling. The number string left to the arrows stands for pooled OR and its 95% CI when FI versus non-FI. *The number of cohorts exceeds the total number of included studies because some studies provided one more different FI definitions-based independent cohorts of eligible data for pooling. *FI* feeding intolerance, *EF* enteral feeding, *GRV* gastric residual volume, *GIS* gastrointestinal symptoms, *NOS* Newcastle–Ottawa Scale, *No. *number, *OR* odds ratio, *CI* confidence interval

Of 5525 records identified, the team assessed 241 full-text manuscripts for eligibility of which 26 studies [5–8, 25–46] involving 10 prospective and 16 retrospectives, which included 25,189 adults in total, proved eligible (Table 1; Fig. 1). Among the included studies, the reported sample size for studied cohorts (one study may have one or more independent cohorts which were analyzed separately) ranged from 20 to 15,918 with the male proportion from 50 to 75% [median (IQR) 63.5% (61–70.8%)], mean age ranging from 33.8 to 71.5 years [median (IQR) 58.1 (53.9–64)], surgery patients proportion ranged from 0 to 100% [median (IQR) 0% (0–21.3%)], mechanical ventilation patients proportion ranged from 26 to 100% [Median (IQR) 100% (82–100%)], and length of follow-up ranged from 13 to 150 days [median (IQR) 30 (24–60)] (Table 1; Additional file 8: Table S6). Five primary and three secondary outcome indicators were pooled in this study and the risks of bias for every outcome indicator if each included FI-definition-differentiated cohorts were separately assessed for the NOS scores. Eventually, a total of outcome-and-cohort-positioned 170 NOS scores with a median score of 7 (4–9) were obtained (Additional file 7: Table S5). Figure 2 shows the summary plots for all-cause mortality. There were 18 different kinds of FI definitions derived from four main categories according to which FI was defined based on any GIS with large GRV, any GIS without large GRV, only large GRV, or EF insufficiency. The detailed data and related GRADE evidence results for all outcome indicators are seen in Additional file 3 (Table S3), and forest plots of partial main results are presented in Additional file 9 (Fig S3).

**Table 1:** Summary characteristics of included studies

Characteristics	Median value (IQR)/cumulative no. of studies (no. of patients)
Eligible studies	
Total no. of studies (no. of patients)	26 (25189)
Prospective	10 (3552)
Retrospective	16 (21637)
Mortality, median (IQR) %	
All-cause hospital mortality	21.9 (15.2–29.3)
All-cause long-term mortality	35.9 (28.9–43.2)
Median (IQR) %, male	63.5 (61–70.8)
Median (IQR), age (years)	58.1 (53.9–64)
Median (IQR) follow-up (days)	30 (24–60)
Country	
America	6 (889)
Australia	3 (476)
China	8 (3185)
Estonia	1 (1712)
France	1 (153)
Greece	1 (46)
Iran	2 (395)
New Zealand	1 (455)
Spain	1 (72)
Multinational	2 (17806)
Diseases/patient state, median (IQR) %	
Surgery	0 (0–21.3)
Trauma	19 (14–54)
Burns	55 (8.8–100)
Digestive	7 (3–9.5)
Sepsis	29 (12–100)
Mechanical ventilation	100 (82–100)
FI definitions	
Defined by GISs	22 (8441)
Defined by large GRV	8 (1381)
Defined by EF insufficiency	8 (3855)
Defined by no. of GISs	16 (7399)
Defined by a specific symptom	12 (2370)

*IQR* interquartile range, *ICU* intensive care unit, *FI* feeding intolerance, *GISs* gastrointestinal symptoms, *GRV* gastric residual volume, *EF* enteral feeding

### Relative effects overall and by different FI definitions

**Fig. 3:**
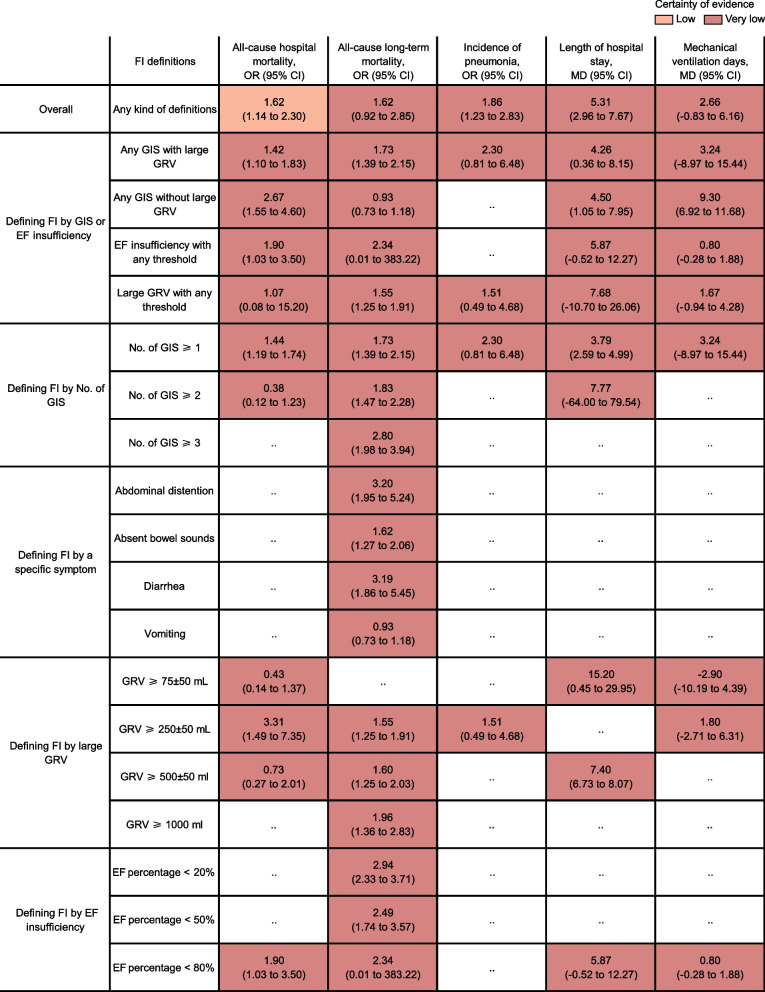
Data summary of relative effects overall and by different FI definitions. The relative effects overall and across 18 different kinds of FI definitions were measured as ORs for all-cause hospital mortality, long-term mortality, and incidence of pneumonia, as well as MDs for the length of hospital stay, and mechanical ventilation, along with 95% CIs. The color of each cell indicates the certainty of evidence according to the Grading of Recommendations Assessment, Development, and Evaluation. *FI* feeding intolerance, *GIS* gastrointestinal symptoms, *GRV* gastric residual volume, *EF* enteral feeding, *No.* number, *OR* odds ratio, *MD* mean difference, *CI* confidence interval

Figure 3 shows a summary of relative effects for the five primary outcomes overall and across 18 different kinds of FI definitions. By and large, when it did not distinguish between defined types FI was significantly associated with all-cause hospital mortality (OR 1.62, 95% CI 1.14–2.30; low certainty evidence; Fig. 3), the incidence of pneumonia (OR 1.86, 95% CI 1.23–2.83; very low certainty evidence; Fig. 3) and length of hospital stay (MD 5.31, 95% CI 2.96–7.67; very low certainty evidence; Fig. 3). FI usually presents with GIS such as large GRV (36.11%, 95% CI 20.75–51.48%), vomiting (defined as any visible regurgitation of gastric contents in most studies or emesis ˃100 mL × 2 episodes [26]; 18.68%, 95% CI 5.87–31.5%), absent bowel sounds (detected by auscultation which was daily performed, 15.54%, 95% CI 3.65–27.42%), abdominal distension (suspected clinically or increased abdominal girth measured by the clinician or radiologically confirmed, 12.19%, 95% CI − 4.07% to 28.45%), and diarrhea (having 3 or more loose or liquid stools per day with a stool weight > 200–250 g/day or > 250 mL/day (5.24% 95% CI 1.32–9.17%) (the pooled prevalence comes from the cases of calculating all-cause mortality rates; Additional file 10: Table S7). Due to insufficient data, reflux aspiration or aspiration pneumonia was analyzed only as one outcome situation that embraced generalized pneumonia rather than an independent variable. Provided that it was greater than or equal to 250 ± 50 mL (per 24 h on a single calendar day), large GRV alone was significantly associated with high all-cause hospital mortality (OR 3.31, 95% CI 1.49–7.35; very low certainty evidence; Fig. 3) and all-cause long-term mortality (OR 1.55, 95% CI 1.25–1.91; very low certainty evidence; Fig. 3). Except for vomiting, large GRV alone no matter with any threshold or GRV ≥ 250 ± 50 mL/500 ± 50 mL, abdominal distention, absent bowel sounds, and diarrhea seemed all to be associated with high all-cause long-term mortality (very low certainty evidence; Fig. 3). When FI was determined according to GIS, large GRV was more likely a powerful candidate symptom which nearly doubled the risk of all-cause long-term mortality compared to without it (very low certainty evidence; Fig. 3). The risk of all-cause long-term mortality went up with the increase in the number of GIS (at least one symptom of large GRV) which determined FI (Fig. 3). The number of GIS ≥ 3 was 1.5 times the risk of all-cause long-term mortality when compared to the number of GIS ≥ 1, while the number of GIS ≥ 2 had a similar risk with GIS ≥ 1 (very low certainty evidence; Fig. 3). When FI has been defined as EF insufficiency with threshold indiscriminately or with EF percentage < 80% [cohorts with EF percentage < 20% and < 50% were completely covered by the same cohort [5] with EF percentage < 80%], it was associated with hospital mortality but with a marginal significance (OR 1.90, 95% CI 1.03–3.50; very low certainty evidence; Fig. 3) and showed no significant difference for other outcomes (Fig. 3). Similar results were found in the three secondary outcomes (Additional file 11: Fig S4).

### Subgroup analyses for relative effects

**Fig. 4:**
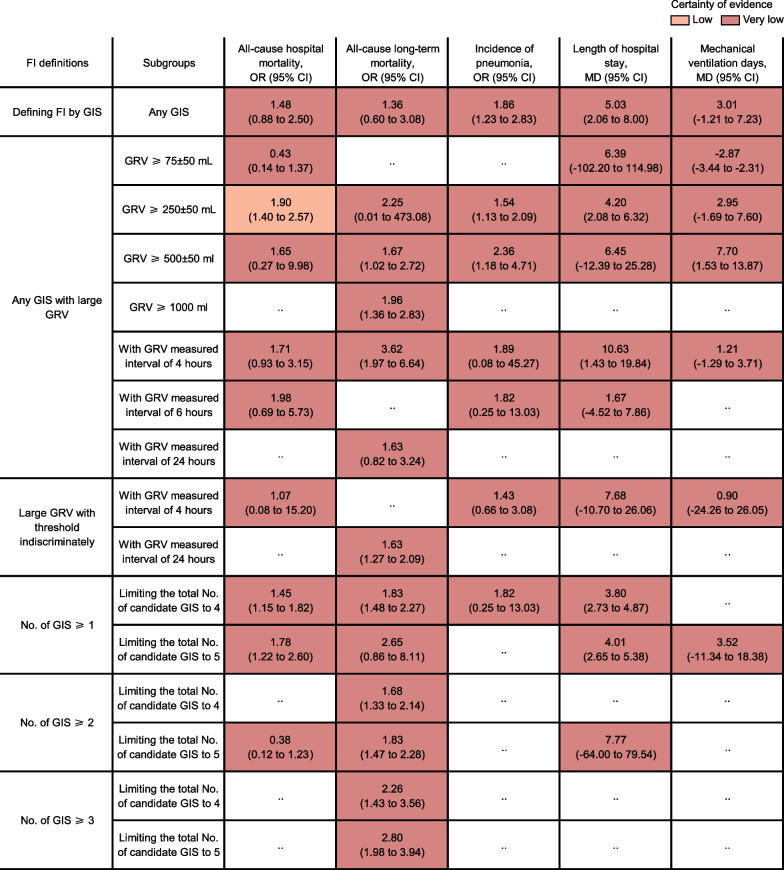
Subgroup analyses according to FI definitions-related key elements. The certainty of the evidence was rated by the Grading of Recommendations Assessment, Development, and Evaluation criteria. GIS here no matter in candidate GIS or selected GIS to determine FI at least included large GRV. GIS is referred to as a large GRV alone or a large GRV combined with another one or any combination of symptoms including vomiting, absent bowel sounds, abdominal distension, and diarrhea. Data were expressed as ORs and MDs along with their 95% CIs. *FI *feeding intolerance, *GIS* gastrointestinal symptoms, *GRV* gastric residual volume, *No.* number, *OR* odds ratio, *MD* mean difference, *CI *confidence interval

Further subgroup analyses were conducted for exploring the impact on outcomes in each kind of FI definition framework of such variation as thresholds for large GRV or lengths of GRV measured intervals or the total numbers of candidate GIS involved in relevant FI definitions; the impact of the different characteristic levels of the study patients on the overall associations between outcomes and FI of any kind of definitions was also explored (Fig. 4; Additional file 4: Fig S1; Additional file 12: Fig S5; Additional file 13: Fig S6). When large GRV was combined with any other GIS, there was GRV ≥ 250 ± 50 mL having a significant impact on all-cause hospital mortality (OR 1.90, 95% CI 1.40–2.57; low certainty evidence; Fig. 4), the incidence of pneumonia (OR 1.54, 95% CI 1.13–2.09; very low certainty evidence; Fig. 4) and length of hospital stay (MD 4.20, 95% CI 2.08–6.32; very low certainty evidence; Fig. 4). No matter whether GRV ≥ 250 ± 50 mL or 500 ± 50 mL, a high incidence of pneumonia occurred in patients with large GRV combined with any other GIS (very low certainty evidence; Fig. 4). However, GRV ≥ 250 ± 50 mL alone was not associated with the incidence of pneumonia (very low certainty evidence; Fig. 3). No significant difference was found among GRV measured intervals of 4, 6, and 24 h in most cases, however, the risk of all-cause long-term mortality was higher and the length of hospital stay was longer in the 4-h measurement interval when compared to other intervals (Fig. 4). Subgroup analyses by limiting the total number of candidate GIS to 4 or 5 proved consistent with the primary results regarding the impact of the number of GIS-determined-FI on outcomes (Fig. 4). Relative effects of FI on all outcomes in the overall population disregarding any kind of FI definitions were conducted across 26 characteristic levels of study patients (Additional file 4: Fig S1; Additional file 13: Fig S6). From the perspective of the underlying disease composition, the risk of all-cause hospital mortality of FI went up with the increase of the surgical proportion (especially abdominal surgery), and sepsis proportion (Additional file 4: Fig S1).

### Absolute effects overall and by different FI definitions

**Fig. 5:**
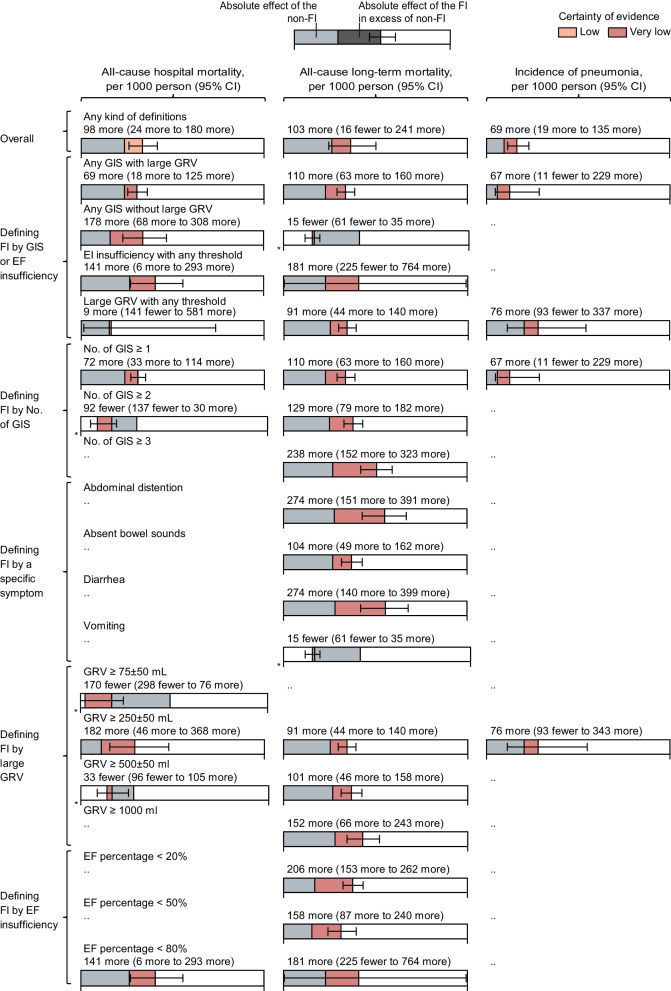
Data summary of absolute effects on all-cause hospital mortality, long-term mortality, and incidence of pneumonia overall and by FI definitions. Effects of FI and non-FI changed together in pairs with the different definitions of FI. Absolute effects of the FI in excess of non-FI were estimated overall or across 18 different FI definitions via a random-effect meta-analysis of rates. This pooled effect represents how many more patients with poor outcomes can expect to occur due to FI. *Axis starting point is -200 per 1000 events. *FI* feeding intolerance, *GIS* gastrointestinal symptoms, *GRV* gastric residual volume, *EF* enteral feeding, *No.* number, *CI* confidence interval

Absolute effects of the FI over non-FI varied greatly with the different definitions for FI because every change of requirements in definitions for FI brought about the re-division of the exposed cohort and non-exposed cohort. Overall, FI resulted in 98 more patients per 1000 person-years contributing to all-cause hospital mortality (low certainty evidence), 103 more patients per 1000 person-years contributing to all-cause long-term mortality (very low certainty evidence), and 69 more patients per 1000 person-years contributing to the incidence of pneumonia (very low certainty evidence) (Fig. 5). For the outcome of all-cause hospital mortality, FI was defined as any other GIS combined with large GRV, any GIS without large GRV, EF insufficiency with any threshold, and large GRV with any threshold resulting in, respectively, 69, 178, 141, and 9 more dead patients than the corresponding non-FI (very low certainty evidence; Fig. 5). FI defined as GRV ≥ 250 ± 50 mL resulted in 182 more all-cause hospital death, 91 more all-cause long-term death, and 76 more pneumonia occurrence than the corresponding non-FI (very low certainty evidence; Fig. 5). Overall absolute effects of FI on all-cause mortality and all-cause ICU mortality showed similar results with all-cause hospital mortality (Additional file 14: Fig S7).

### FI prevalence overall and by different FI definitions

**Fig. 6:**
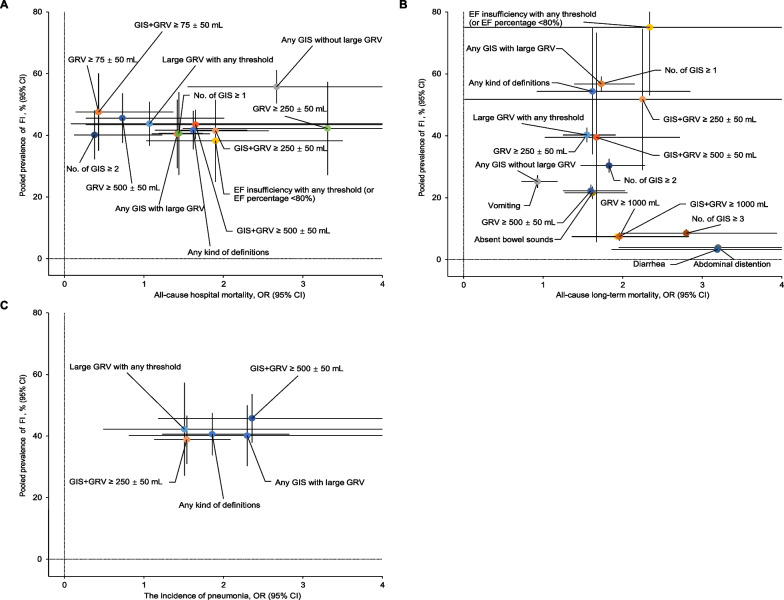
Two-dimensional graphs of FI prevalence versus all-cause hospital mortality, long-term mortality, and the incidence of pneumonia overall and by different FI definitions. **A** All-cause hospital mortality versus FI prevalence. **B** All-cause long-term mortality versus FI prevalence. **C** The incidence of pneumonia versus FI prevalence. Effect sizes for FI by different definitions are represented by colored nodes, with bars representing the corresponding 95% CIs. FI prevalence by EF percentage < 20% or < 50% was not pooled due to their cohorts being completely covered by the same cohort with EF percentage < 80% in our study, if not, significant errors of selectivity were expected. *FI *feeding intolerance, *EF* enteral feeding, *GRV* gastric residual volume, *GIS* gastrointestinal symptoms, *No.* number, *OR* odds ratio, *CI* confidence interval

We pooled the proportion of FI under different FI definitions and used two-dimensional graphs to present the relationship between FI prevalence rates and the risk of poor outcomes (all-cause hospital mortality, all-cause long-term mortality, and the incidence of pneumonia) (Fig. 6). Overall, FI, any kind of definitions, had a pooled prevalence of 41.68% (95% CI 35.46–47.9%) with an OR of 1.62 (95% CI 1.14–2.3) for all-cause hospital mortality or a pooled prevalence of 54.37% (95% CI 34.02–74.72%) with an OR of 1.62 (95% CI 0.92–2.85) for all-cause long-term mortality or a pooled prevalence of 40.59% (95% CI 33.73–47.44%) with an OR of 1.86 (95% CI 1.23–2.83) for the incidence of pneumonia (Fig. 6; Additional file 10: Table S7). EF insufficiency with any threshold (EF percentage < 80%) resulted in 38.23% (95% CI 24.88–51.58) of FI prevalence while 1.9 (95% CI 1.03–3.5) of OR for all-cause hospital mortality, or resulted in 75.05% (95% CI 53.02–97.09) of FI prevalence while 2.34 (95% CI 0.01–383.22) of OR for all-cause long-term mortality (Fig. 6; Additional file 10: Table S7). Compared to any GIS without large GRV, any GIS with large GRV, in other words, the addition of large GRV promoted OR of all-cause long-term mortality from 0.93 (95% CI 0.73–1.18) to 1.73 (95% CI 1.39–2.15) with a rise in FI prevalence (25.23%, 95% CI 23.18–27.29% vs 56.78%, 95% CI 54.43–59.12%). GIS + GRV ≥ 250 ± 50 mL had moderate all-cause hospital mortality (1.9, 95% CI 1.4–2.57) and a moderate FI prevalence (41.49%, 95% CI 31.61–51.38%) (Fig. 6 and Additional file 10: Table S7). GIS + GRV ≥ 500 ± 50 mL had a high incidence of pneumonia (2.36, 95% CI 1.18–4.71) and a moderate FI prevalence (45.75%, 95% CI 37.86–53.65%) (Fig. 6; Additional file 10: Table S7). The no. of GIS ≥ 1 had moderate all-cause hospital mortality (1.44, 95% CI 1.19–1.74), and a moderate FI prevalence (40.6%, 95% CI 27.15–54.05%) (Fig. 6; Additional file 10: Table S7). Although the no. of GIS ≥ 3 and GRV ≥ 1000 mL could raise OR of all-cause long-term mortality to 2.8 (95% CI 1.98–3.94) and 1.96 (95% CI 1.36–2.83), respectively, while their prevalence dropped to 8.53% (95% CI 7.21–9.85) and 7.48% (95% CI 6.23–8.72) accordingly (Fig. 6; Additional file 10: Table S7). Our analysis also showed similar two-dimensional results of FI prevalence versus all-cause mortality and all-cause ICU mortality (Additional file 10: Table S7; Additional file 15: Fig S8).

### Sensitivity analyses and publication bias

Sensitivity analyses by removal of the only multi-center prospective study [32] written in Chinese proved consistent with the primary results (Additional file 16: Fig S9). Although Egger’s test results and funnel plots for main outcomes proved no significant publication bias, the number of studies for data pooling of all-cause long-term mortality, pneumonia rate, and mechanical ventilation days was less than 10, which may increase the risk of publication bias (Additional file 5: Table S4; Additional file 6: Fig S2).

## Discussion

This meta-analysis involving 26 studies that included 25,189 adults provided low to very low certainty evidence that FI defined as large GRV (GRV ≥ 250 ± 50 mL) combined with any other GIS has a significant association with high all-cause hospital mortality, high incidence of pneumonia, and prolonged length of hospital stay in critically ill patients receiving EN, with a moderate FI prevalence. Although related to adverse outcomes, EF insufficiency with any threshold (EF percentage < 80%) marginally correlates with all-cause hospital mortality resulting in the second preferred definition for FI during EN in critically ill patients. Moreover, very low certainty evidence indicated that surgical patients (especially those who underwent abdominal surgery) and sepsis patients were more likely to have higher mortality risk associated with FI disregarding the kinds of FI definitions.

Our findings answered the question that to what extent are FI and outcomes of critically ill adults receiving EN associated across different definitions through a quantitative meta-analysis. The FI classification in our review is consistent with and more refined compared with two previous systematic reviews separately in 2014 and 2022 as well as an invited review in 2021 summarizing from the literature the existing FI types [1, 4, 11]. The systematic review in 2014 reported a pooled total FI prevalence of 38.3% from 31 studies involving 4339 patients in intensive care [4]. Our findings are in line, but we almost doubled the number of patients for pooling regarding ICU prevalence despite fewer studies being involved due to the topic requirements. The review in 2021 demonstrated divergent outcomes regarding GRV’s role in FI judgment and proposed a definition of FI as a failure to reach EN targets plus the presence of GIS only based on qualitative evidence with more empirical inference[11].

FI usually presents with GIS such as large GRV, vomiting, absent bowel sounds, abdominal distension, and diarrhea. Our findings show a high risk of all-cause long-term mortality with single GIS except for vomiting. As one of the GIS, however, GRV is difficult to measure in standard using the aspiration method which is widely used now [9, 47]. In reality, clarifying the role of GRV is complex—the undetermined role of GRV combined with other GISs rather than GRV alone in FI judgment, the unclear role of continuous monitoring of GRV, and the rise of bedside ultrasonic as a substitute for manual aspiration for measurement of GRV. Therefore, it is urgent to solve the problem of how to reasonably use large GRV in determining FI in clinical practice. Our findings showed that compared to GIS without large GRV, any GIS with large GRV had a higher risk for all-cause long-term mortality, and with the increase in the minimum number of GIS including large GRV used to determine FI, the risk of all-cause long-term mortality increased. Thus, a large GRV-centered GIS combination is a preferred alternative for the current clinical definition of FI.

At present, it is generally accepted that EN should not be stopped automatically when GRV < 500 mL and no other intolerance of EN [48]. But what if in the case where GRV ≥ 250 ± 50 mL coincides with other intolerance of EN? Our findings showed GRV ≥ 250 ± 50 mL in conjunction with the other GIS presented a significant association with hospital mortality. Although several studies [49, 50] have shown that GRV was unable to accurately predict the risk of reflux and aspiration in critical care patients, large GRV (≥ 250 ± 50 mL) combined with any other GIS instead of GRV ≥ 250 ± 50 mL alone was associated with a high incidence of pneumonia in our findings.

As far as we know, our review is the first study on the association between different definitions of FI and patient-centered outcomes of critically ill adults receiving EN, through the most comprehensive synthesis of evidence to date. With the backing of a nationwide multidisciplinary guideline panel in formulating the clinical questions, selecting patient-centered outcomes, and subgroup analyses, the review ensured relevance for clinical practice. We used the most up-to-date approaches to assess and present the findings using GRADE frameworks, with the integration of the NOS score results and adoption of MID to determine rating down for risk of bias or imprecision. Moreover, we considered the prevalence of and critical thresholds in each kind of definition at the same time, allowing for judgment considering the size of the target population and clinical applicability in practice.

Limitations of our review include the absence of RCTs’ data for pooling due to restriction by the special study topic for which only observational studies were available at present, which increased the risk of bias regarding comparability between exposure and non-exposure cohorts. Furthermore, the lack of individual patient data for pooling particularly weakened the precision of synthesis for subgroup effects. Studies varied in outcomes reporting, resulting in some outcomes such as the incidence of pneumonia and mechanical ventilation days failing to be compared under certain specific FI definitions. Due to limited data, we investigated common but not all GIS and failed to study the relevance among them.

## Conclusions

In conclusion, the large-GRV-centered GIS to define FI with a short-time window for diagnosis proved to have a significant association with all-cause hospital mortality, high incidence of pneumonia, and prolonged length of hospital stay together with a moderate FI prevalence in critically ill patients receiving EN. 3-day EF-insufficiency had a marginally significant association with all-cause hospital mortality. The evidence for the FI’s associations with outcomes provides the reference of these findings as a basis for the definition development of FI in critical care, but high-quality confirmatory studies are warranted in the future.

## Supplementary Information

**Additional file 1. Table S1:** PRISMA checklist.

**Additional file 2. Table S2:** Search strategy.

**Additional file 3. Table S3:** GRADE evidence profile

**Additional file 4. Fig S1:** Subgroup analyses for hospital/long-term mortality, and incidence of pneumonia, as well as length of hospital stay, and mechanical ventilation days according to characteristic levels of the study patients regardless of the kinds of FI definitions.

**Additional file 5. Table S4:** Egger’s test results for main outcomes.

**Additional file 6. Fig S2:** Funnel plots for main outcome indicators.

**Additional file 7. Table S5:** Risk of bias of included studies according to pooled indicators

**Additional file 8. Table S6:** Characteristics of included studies.

**Additional file 9. Fig S3** Forest plots of partial main results.

**Additional file 10. Table S7:** FI pooled mortality/incidence of pneumonia and prevalence across different definitions

**Additional file 11. Fig S4:** Data summary of relative effects for all-cause mortality, all-cause ICU mortality and length of ICU stay overall and by different FI definitions.

**Additional file 12. Fig S5:** Subgroup analyses for all-cause mortality, all-cause ICU mortality and length of ICU stay according to FI definition-related key elements.

**Additional file 13. Fig S6:** Subgroup analyses for all-cause mortality and all-cause ICU mortality, as well as the length of ICU stay according to characteristic levels of the study patients regardless of the kinds of FI definitions.

**Additional file 14. Fig S7:** Data summary of absolute effects on all-cause mortality and all-cause ICU mortality overall and by FI definitions.

**Additional file 15. Fig S8:** Two-dimensional graphs of FI prevalence versus all-cause mortality and all-cause ICU mortality by different FI definitions.

**Additional file 16. Fig S9:** Forest plots of sensitivity analysis.

## Data Availability

Not applicable.

## Abbreviations

FI: Feeding intolerance
EN: Enteral nutrition
ESICM: European Society of Intensive Care Medicine
GRV: Gastric residual volume
GIS: Gastrointestinal symptoms
RCT: Randomized controlled trial
ICU: Intensive care unit
MOOSE: Meta-analysis Of Observational Studies in Epidemiology
PRISMA: Preferred Reporting Items for Systematic Reviews and Meta-Analyses
OR: Odds ratio
MD: Mean difference
SOFA: Sequential Organ Failure Assessment
APACHE II: Acute Physiology and Chronic Health Evaluation II
NOS: Newcastle–Ottawa Scale
GRADE: Grading of Recommendations Assessment, Development, and Evaluation
MID: Minimally important difference
EF: Enteral feeding
CI: Confidence interval
CSA: Cross-sectional area
MI: Motion index
