# Supplementary material for: Different definitions of feeding intolerance and their associations with outcomes of critically ill adults receiving enteral nutrition: a systematic review and meta-analysis
Source: J Intensive Care. 2023 Jul 5;11:29. doi: 10.1186/s40560-023-00674-3 (PMC10320932; doi:10.1186/s40560-023-00674-3)
Supplement: Supplementary file 2 — Additional file 2. Table S2: Search strategy. [file 40560_2023_674_MOESM2_ESM.docx]

# Table S2: Search strategy

| - PubMed (inception to April 26, 2022) (n=1041 results)  1. "Critical Care"[Mesh] 2. "Critical Illness"[Mesh] 3. "intensive care units"[Mesh] 4. "critical care"[Title/Abstract] 5. "critical illness"[Title/Abstract] 6. "critically ill"[Title/Abstract] 7. "intensive care"[Title/Abstract] 8. "ICU"[Title/Abstract] 9. OR/#1-8 10. "Nutrition Therapy"[Mesh] 11. "Nutritional Support"[Mesh] 12. "Enteral Nutrition"[Mesh] 13. "Parenteral Nutrition"[Mesh] 14. "Nutrition Therap*"[Title/Abstract] 15. "Nutritional Support*"[Title/Abstract] 16. "Artificial Feeding*"[Title/Abstract] 17. "Nutritional Requirement*"[Title/Abstract] 18. "Enteral Nutrition"[Title/Abstract] 19. "Enteral Feeding*"[Title/Abstract] 20. "Force Feeding*"[Title/Abstract] 21. "Tube Feeding*"[Title/Abstract] 22. "Gastric Feeding Tubes*"[Title/Abstract] 23. "Parenteral Nutrition*"[Title/Abstract] 24. "Parenteral Feeding*"[Title/Abstract] 25. "Intravenous Feeding*"[Title/Abstract] 26. OR/#10-25 27. "drug tolerance"[Mesh] 28. "immune tolerance"[Mesh] 29. "Self Tolerance"[Mesh] 30. intolerab*[Title/Abstract] 31. intoleran*[Title/Abstract] 32. tolerab*[Title/Abstract] 33. toleran*[Title/Abstract] 34. tolerat*[Title/Abstract] 35. OR/#27-34 36. #9 AND #26 AND #35 | - Web of Science (inception to April 26, 2022) (n=1565 results)  1. "critical illness"[Topic] 2. "critical care"[Topic] 3. "Intensive Care Units"[Topic] 4. "ICU"[Topic] 5. "intensive care"[Topic] 6. "Critically Ill"[Topic] 7. OR/#1-6 8. "Nutrition Therap*"[Topic] 9. "Nutritional Support*"[Topic] 10. "Artificial Feeding*"[Topic] 11. "Nutritional Requirement*"[Topic] 12. "Enteral Nutrition"[Topic] 13. "Enteral Feeding*"[Topic] 14. "Force Feeding*"[Topic] 15. "Tube Feeding*"[Topic] 16. "Gastric Feeding Tubes*"[Topic] 17. "Parenteral Nutrition*"[Topic] 18. "Parenteral Feeding*"[Topic] 19. "Intravenous Feeding*"[Topic] 20. OR/#8-19 21. intolerab*[Topic] 22. intoleran*[Topic] 23. tolerab*[Topic] 24. toleran*[Topic] 25. tolerat*[Topic] 26. OR/#21-25 27. #7 AND #20 AND #26 |
| --- | --- |
| - The Cochrane Library (inception to April 26, 2022) (n=3 results)  1. MeSH descriptor: [Critical Illness] explode all trees 2. MeSH descriptor: [Critical Care] explode all trees 3. MeSH descriptor: [Intensive Care Units] explode all trees 4. ("critical illness"):ti,ab,kw 5. ("critical care"):ti,ab,kw 6. ("ICU"):ti,ab,kw 7. ("intensive care"):ti,ab,kw 8. ("Critically Ill"):ti,ab,kw 9. OR/#1-8 10. MeSH descriptor: [Nutrition Therapy] explode all trees 11. MeSH descriptor: [Nutritional Support] explode all trees 12. MeSH descriptor: [Enteral Nutrition] explode all trees 13. MeSH descriptor: [Parenteral Nutrition] explode all trees 14. ("Nutrition Therap*"):ti,ab,kw 15. ("Nutritional Support*"):ti,ab,kw 16. ("Artificial Feeding*"):ti,ab,kw 17. ("Nutritional Requirement*"):ti,ab,kw 18. ("Enteral Nutrition"):ti,ab,kw 19. ("Enteral Feeding*"):ti,ab,kw 20. ("Force Feeding*"):ti,ab,kw 21. ("Tube Feeding*"):ti,ab,kw 22. ("Gastric Feeding Tubes*"):ti,ab,kw 23. ("Parenteral Nutrition*"):ti,ab,kw 24. ("Parenteral Feeding*"):ti,ab,kw 25. ("Intravenous Feeding*"):ti,ab,kw 26. OR/#10-25 27. MeSH descriptor: [Drug Tolerance] explode all trees 28. MeSH descriptor: [Immune Tolerance] explode all trees 29. MeSH descriptor: [Self Tolerance] explode all trees 30. ("intolerab*"):ti,ab,kw 31. ("intoleran*"):ti,ab,kw 32. ("tolerab*"):ti,ab,kw 33. ("toleran*"):ti,ab,kw 34. ("tolerat*"):ti,ab,kw 35. OR/#27-34 36. #9 AND #26 AND #35 | - CBM (search in Chinese, inception to April 26, 2022) (n=834 results)   The keywords presented below are phonetic transcription for Chinese characters   1. "zhongzheng"[Common fields: intelligence] 2. "weizhong"[Common fields: intelligence] 3. "zhongzhengjianhu "[Common fields: intelligence] 4. "ICU"[Common fields: intelligence] 5. OR/#1-4 6. "yingyangzhiliao"[Common fields: intelligence] 7. "yingyangzhichi"[Common fields: intelligence] 8. "changdaoyingyang "[Common fields: intelligence] 9. "changneiyingyang "[Common fields: intelligence] 10. "changsi"[Common fields: intelligence] 11. "guansi"[Common fields: intelligence] 12. "qiangzhiweiyang"[Common fields: intelligence] 13. "weichangwaiyingyang"[Common fields: intelligence] 14. OR/#6-13 15. "naishou"[Common fields: intelligence] 16. #5 AND #14 AND #15 |
| - CNKI (search in Chinese, inception to April 26, 2022) (n=544 results)   The keywords presented below are phonetic transcription for Chinese characters   1. "zhongzheng"(TOPIC) 2. "weizhong"(TOPIC) 3. "zhongzhengjianhu"(TOPIC) 4. "ICU"(TOPIC) 5. OR/#1-4 6. "yingyangzhiliao"(TOPIC) 7. "yingyangzhichi"(TOPIC) 8. "changdaoyingyang"(TOPIC) 9. "changneiyingyang"(TOPIC) 10. "changsi"(TOPIC) 11. "guansi"(TOPIC) 12. "qiangzhiweiyang"(TOPIC) 13. "weichangwaiyingyang"(TOPIC) 14. OR/#6-13 15. "naishou "(TOPIC) 16. #5 AND #14 AND #15 | - WanFang (search in Chinese, inception to April 26, 2022) (n=1240 results)   The keywords presented below are phonetic transcription for Chinese characters   1. TOPIC:("zhongzheng") 2. TOPIC:("weizhong") 3. TOPIC:("zhongzhengjianhu") 4. TOPIC:("ICU") 5. OR/#1-4 6. TOPIC:("yingyangzhiliao") 7. TOPIC:("yingyangzhichi") 8. TOPIC:("changdaoyingyang ") 9. TOPIC:("changneiyingyang") 10. TOPIC:("changsi") 11. TOPIC:("guansi") 12. TOPIC:("qiangzhiweiyang") 13. TOPIC:("weichangwaiyingyang") 14. OR/#6-13 15. TOPIC:("naishou ") 16. #5 AND #14 AND #15 |
| - ClinicalTrials.gov (inception to April 26, 2022) (n= 29 results)   Condition or disease:  "Critical Care" OR "Critical Illness" OR "intensive care units" OR "critical care" OR "critical illness" OR "critically ill" OR "intensive care" OR "ICU"  Intervention/treatment:  "Nutrition Therapy" OR "Nutritional Support" OR "Parenteral Nutrition" OR ("Nutrition Therap*" OR "Nutritional Support*" OR "Artificial Feeding*" OR "Nutritional Requirement*" OR "Enteral Nutrition" OR "Enteral Feeding*" OR "Force Feeding*" OR "Tube Feeding*" OR "Gastric Feeding Tubes*" OR "Parenteral Nutrition*" OR "Parenteral Feeding*" OR "Intravenous Feeding*"  Outcome Measure:  "drug tolerance" OR "immune tolerance" OR "Self Tolerance" OR intolerab* OR intoleran* OR tolerab* OR toleran* OR tolerat*  Applied Filters:  Completed |  |
