# Supplementary material for: Different definitions of feeding intolerance and their associations with outcomes of critically ill adults receiving enteral nutrition: a systematic review and meta-analysis
Source: J Intensive Care. 2023 Jul 5;11:29. doi: 10.1186/s40560-023-00674-3 (PMC10320932; doi:10.1186/s40560-023-00674-3)
Supplement: Supplementary file 3 — Additional file 3. Table S3: GRADE evidence profile [file 40560_2023_674_MOESM3_ESM.docx]

# Table S3: GRADE evidence profile

| **Question: FI compared to non-FI in critically ill adults receiving enteral nutrition^a^** | | | | | | | | | | | | |
| --- | --- | --- | --- | --- | --- | --- | --- | --- | --- | --- | --- | --- |
| **Certainty assessment** | | | | | | | **№ of patients** | | **Effect** | | **Certainty** | **Importance** |
| **№ of studies/cohorts** | **Study design** | **Risk of bias** | **Inconsistency** | **Indirectness** | **Imprecision** | **Other considerations** | **FI** | **non-FI** | **Relative (95% CI)** | **Absolute (95% CI)** |  |  |
| **All-cause ICU mortality (follow-up: range 20 days to 90+ days; assessed with: medical records)^bc^** | | | | | | | | | | | | |
| 17 | observational studies | not serious | not serious | not serious | not serious | none | 881/4488 (19.6%) ^e^ | 982/7099 (13.8%) ^e^ | **OR 1.99** (1.69 to 2.35) | **104 more per 1,000** (from 75 more to 136 more) | ⨁⨁◯◯ Low | IMPORTANT |
| **All-cause hospital mortality (follow-up: range 13 days to 150+ days; assessed with: medical records)^f^** | | | | | | | | | | | | |
| 16 | observational studies | not serious | not serious | not serious | not serious | none | 1799/5808 (31.0%) ^e^ | 3490/14748 (23.7%) ^e^ | **OR 1.62** (1.14 to 2.30) | **98 more per 1,000** (from 24 more to 180 more) | ⨁⨁◯◯ Low | CRITICAL |
| **All-cause long-term mortality (follow-up: range 90 days to 150+ days; assessed with: follow-up records)^g^** | | | | | | | | | | | | |
| 5 | observational studies | not serious | not serious | not serious | serious^h^ | publication bias strongly suspected^i^ | 1034/3129 (33.0%) ^e^ | 852/3266 (26.1%) ^e^ | **OR 1.62** (0.92 to 2.85) | **103 more per 1,000** (from 16 fewer to 241 more) | ⨁◯◯◯ Very low | CRITICAL |
| **All-cause mortality (follow-up: range 13 days to 150+ days; assessed with: medical records)** | | | | | | | | | | | | |
| 28^d^ | observational studies | not serious | not serious | not serious | not serious | none | 3018/9848 (30.6%) ^e^ | 4799/21046 (22.8%) ^e^ | **OR 1.67** (1.37 to 2.04) | **102 more per 1,000** (from 60 more to 148 more) | ⨁⨁◯◯ Low | IMPORTANT |
| **Incidence of pneumonia (follow-up: range 13 days to 90+ days; assessed with: medical records)^j^** | | | | | | | | | | | | |
| 4 | observational studies | serious^k^ | not serious | not serious | not serious | publication bias strongly suspected^i^ | 55/315 (17.5%) | 48/502 (9.6%) | **OR 1.86** (1.23 to 2.83) | **69 more per 1,000** (from 19 more to 135 more) | ⨁◯◯◯ Very low | IMPORTANT |
| **Length of ICU stay (follow-up: range 13 days to 60 days; assessed with: medical records; Scale from: 10 to 30)** | | | | | | | | | | | | |
| 22 | observational studies | serious^k^ | not serious | not serious | not serious | none | 10244 | 28983 | - | MD **3.6 days more** (2.21 more to 5 more) | ⨁◯◯◯ Very low | IMPORTANT |
| **Length of hospital stay (follow-up: range 20 days to 60 days; assessed with: medical records; Scale from: 10 to 50)** | | | | | | | | | | | | |
| 13 | observational studies | serious^k^ | not serious | not serious | not serious | none | 5997 | 15265 | - | MD **5.31 days more** (2.96 more to 7.67 more) | ⨁◯◯◯ Very low | IMPORTANT |
| **Mechanical ventilation days (follow-up: range 13 days to 90+ days; assessed with: medical records; Scale from: 7 to 26)** | | | | | | | | | | | | |
| 8 | observational studies | serious^k^ | not serious | not serious | not serious | publication bias strongly suspected^i^ | 545 | 745 | - | MD **2.66 days more** (0.83 fewer to 6.16 more) | ⨁◯◯◯ Very low | IMPORTANT |
| **All-cause ICU mortality after redefining exposed cohort by defining FI according to GI symptoms cluster (follow-up: range 20 days to 90+ days; assessed with: medical records)** | | | | | | | | | | | | |
| 14 | observational studies | not serious | not serious | not serious | not serious | none | 657/3289 (20.0%) | 822/6339 (13.0%) | **OR 2.02** (1.64 to 2.48) | **102 more per 1,000** (from 67 more to 140 more) | ⨁⨁◯◯ Low | IMPORTANT |
| **All-cause ICU mortality after redefining exposed cohort by defining FI according to large-GRV-containing GI symptoms cluster (follow-up: range 20 days to 90+ days; assessed with: medical records)** | | | | | | | | | | | | |
| 8 | observational studies | not serious | not serious | not serious | not serious | publication bias strongly suspected^i^ | 377/1800 (20.9%) | 246/2111 (11.7%) | **OR 2.29** (1.98 to 2.65) | **115 more per 1,000** (from 91 more to 142 more) | ⨁◯◯◯ Very low | IMPORTANT |
| **All-cause ICU mortality after redefining exposed cohort by defining FI according to GI symptoms cluster without large GRV (follow-up: range 20 days to 90+ days; assessed with: medical records)** | | | | | | | | | | | | |
| 3 | observational studies | not serious | serious^l^ | not serious | serious^h^ | publication bias strongly suspected^i^ | 146/766 (19.1%) | 283/2082 (13.6%) | **OR 1.33** (0.29 to 5.99) | **37 more per 1,000** (from 92 fewer to 349 more) | ⨁◯◯◯ Very low | IMPORTANT |
| **All-cause ICU mortality after redefining exposed cohort by defining FI according to only large GRV (follow-up: range 20 days to 90+ days; assessed with: medical records)** | | | | | | | | | | | | |
| 3 | observational studies | not serious | not serious | not serious | not serious | publication bias strongly suspected^i^ | 134/723 (18.5%) | 293/2146 (13.7%) | **OR 2.31** (1.63 to 3.27) | **131 more per 1,000** (from 68 more to 204 more) | ⨁◯◯◯ Very low | IMPORTANT |
| **All-cause ICU mortality after redefining exposed cohort by defining FI according to EF insufficiency (follow-up: range 20 days to 90+ days; assessed with: medical records)** | | | | | | | | | | | | |
| 3 | observational studies | not serious | not serious | not serious | serious^m^ | publication bias strongly suspected^i^ | 224/1199 (18.7%) | 160/760 (21.1%) | **OR 1.87** (1.08 to 3.24) | **122 more per 1,000** (from 13 more to 253 more) | ⨁◯◯◯ Very low | IMPORTANT |
| **All-cause ICU mortality after redefining exposed cohort by defining FI according to the number of GI symptoms ≥1 (no limit to the total number of candidate symptoms, which can range from 1-6) (follow-up: range 20 days to 90 days; assessed with: medical records)** | | | | | | | | | | | | |
| 8 | observational studies | not serious | not serious | not serious | not serious | publication bias strongly suspected^i^ | 377/1800 (20.9%) | 246/2111 (11.7%) | **OR 2.29** (1.98 to 2.65) | **115 more per 1,000** (from 91 more to 142 more) | ⨁◯◯◯ Very low | IMPORTANT |
| **All-cause ICU mortality after redefining exposed cohort by defining FI according to the number of GI symptoms ≥2 (no limit to the total number of candidate symptoms, which can range from 1-6) (follow-up: range 20 days to 90 days; assessed with: medical records)** | | | | | | | | | | | | |
| 2 | observational studies | not serious | not serious | not serious | serious^m^ | publication bias strongly suspected^i^ | 145/623 (23.3%) | 276/2187 (12.6%) | **OR 2.61** (1.07 to 6.37) | **148 more per 1,000** (from 8 more to 353 more) | ⨁◯◯◯ Very low | IMPORTANT |
| **All-cause ICU mortality after redefining exposed cohort by defining FI according to the number of GI symptoms ≥3 (no limit to the total number of candidate symptoms, which can range from 1-6) (follow-up: range 20 days to 90 days; assessed with: medical records)** | | | | | | | | | | | | |
| 2 | observational studies | not serious | not serious | not serious | not serious | publication bias strongly suspected^i^ | 55/153 (35.9%) | 366/2657 (13.8%) | **OR 4.49** (1.87 to 10.83) | **280 more per 1,000** (from 92 more to 496 more) | ⨁◯◯◯ Very low | IMPORTANT |
| **All-cause ICU mortality after redefining exposed cohort by defining FI according to the number of GI symptoms ≥1 (limit the total number of candidate symptoms to 4) (follow-up: range 20 days to 90+ days; assessed with: medical records)** | | | | | | | | | | | | |
| 4 | observational studies | not serious | not serious | not serious | not serious | publication bias strongly suspected^i^ | 286/1417 (20.2%) | 204/1886 (10.8%) | **OR 2.24** (1.49 to 3.36) | **105 more per 1,000** (from 45 more to 181 more) | ⨁◯◯◯ Very low | IMPORTANT |
| **All-cause ICU mortality after redefining exposed cohort by defining FI according to the number of GI symptoms ≥2 (limit the total number of candidate symptoms to 4) (follow-up: range 20 days to 90+ days; assessed with: medical records)** | | | | | | | | | | | | |
| 2 | observational studies | not serious | not serious | not serious | not serious | publication bias strongly suspected^i^ | 119/480 (24.8%) | 302/2330 (13.0%) | **OR 2.61** (1.44 to 4.74) | **150 more per 1,000** (from 47 more to 284 more) | ⨁◯◯◯ Very low | IMPORTANT |
| **All-cause ICU mortality after redefining exposed cohort by defining FI according to the number of GI symptoms ≥3 (limit the total number of candidate symptoms to 4) (follow-up: range 20 days to 90+ days; assessed with: medical records)** | | | | | | | | | | | | |
| 2 | observational studies | not serious | not serious | not serious | serious^h^ | publication bias strongly suspected^i^ | 26/85 (30.6%) | 395/2725 (14.5%) | **OR 3.09** (0.95 to 10.08) | **199 more per 1,000** (from 6 fewer to 486 more) | ⨁◯◯◯ Very low | IMPORTANT |
| **All-cause ICU mortality after redefining exposed cohort by defining FI according to the number of GI symptoms ≥1 (limit the total number of candidate symptoms to 5) (follow-up: range 20 days to 90+ days; assessed with: medical records)** | | | | | | | | | | | | |
| 5 | observational studies | not serious | not serious | not serious | not serious | publication bias strongly suspected^i^ | 386/1773 (21.8%) | 311/2149 (14.5%) | **OR 2.05** (1.66 to 2.53) | **113 more per 1,000** (from 75 more to 155 more) | ⨁◯◯◯ Very low | IMPORTANT |
| **All-cause ICU mortality after redefining exposed cohort by defining FI according to the number of GI symptoms ≥2 (limit the total number of candidate symptoms to 5) (follow-up: range 20 days to 90+ days; assessed with: medical records)** | | | | | | | | | | | | |
| 2 | observational studies | not serious | not serious | not serious | serious^m^ | publication bias strongly suspected^i^ | 145/623 (23.3%) | 276/2187 (12.6%) | **OR 2.61** (1.07 to 6.37) | **148 more per 1,000** (from 8 more to 353 more) | ⨁◯◯◯ Very low | IMPORTANT |
| **All-cause ICU mortality after redefining exposed cohort by defining FI according to the number of GI symptoms ≥3 (limit the total number of candidate symptoms to 5) (follow-up: range 20 days to 90+ days; assessed with: medical records)** | | | | | | | | | | | | |
| 2 | observational studies | not serious | not serious | not serious | not serious | publication bias strongly suspected^i^ | 55/153 (35.9%) | 366/2657 (13.8%) | **OR 4.49** (1.87 to 10.83) | **280 more per 1,000** (from 92 more to 496 more) | ⨁◯◯◯ Very low | IMPORTANT |
| **All-cause ICU mortality after redefining exposed cohort by defining FI according to symptom of abdominal distention (follow-up: range 28 days to 90 days; assessed with: medical records)** | | | | | | | | | | | | |
| 2 | observational studies | not serious | not serious | not serious | extremely serious^n^ | publication bias strongly suspected^i^ | 107/384 (27.9%) | 314/2426 (12.9%) | **OR 2.86** (0.02 to 481.07) | **169 more per 1,000** (from 126 fewer to 857 more) | ⨁◯◯◯ Very low | IMPORTANT |
| **All-cause ICU mortality after redefining exposed cohort by defining FI according to symptom of absent bowel sounds (follow-up: range 28 days to 90 days; assessed with: medical records)** | | | | | | | | | | | | |
| 2 | observational studies | not serious | serious^l^ | not serious | extremely serious^n^ | publication bias strongly suspected^i^ | 87/408 (21.3%) | 334/2402 (13.9%) | **OR 1.55** (0.00 to 1160.32) | **61 more per 1,000** (from -- to 856 more) | ⨁◯◯◯ Very low | IMPORTANT |
| **All-cause ICU mortality after redefining exposed cohort by defining FI according to symptom of diarrhea (follow-up: range 20 days to 90 days; assessed with: medical records)** | | | | | | | | | | | | |
| 2 | observational studies | not serious | not serious | not serious | serious^h^ | publication bias strongly suspected^i^ | 51/159 (32.1%) | 370/2651 (14.0%) | **OR 2.68** (0.65 to 11.02) | **163 more per 1,000** (from 44 fewer to 502 more) | ⨁◯◯◯ Very low | IMPORTANT |
| **All-cause ICU mortality after redefining exposed cohort by defining FI according to symptom of vomiting (follow-up: range 28 days to 90 days; assessed with: medical records)** | | | | | | | | | | | | |
| 2 | observational studies | not serious | serious^l^ | not serious | very serious^o^ | publication bias strongly suspected^i^ | 81/494 (16.4%) | 340/2316 (14.7%) | **OR 1.49** (0.04 to 52.55) | **57 more per 1,000** (from 140 fewer to 754 more) | ⨁◯◯◯ Very low | IMPORTANT |
| **All-cause ICU mortality after redefining exposed cohort by defining FI according to large GRV (as one of the symptoms) of 500±50 ml (follow-up: range 20 days to 90+ days; assessed with: medical records)** | | | | | | | | | | | | |
| 6 | observational studies | not serious | not serious | not serious | not serious | publication bias strongly suspected^i^ | 152/602 (25.2%) | 384/2722 (14.1%) | **OR 2.52** (1.96 to 3.23) | **152 more per 1,000** (from 102 more to 206 more) | ⨁◯◯◯ Very low | IMPORTANT |
| **All-cause ICU mortality after redefining exposed cohort by defining FI according to large GRV (as one of the symptoms) of 250±50 ml (follow-up: range 20 days to 90+ days; assessed with: medical records)** | | | | | | | | | | | | |
| 6 | observational studies | not serious | not serious | not serious | not serious | publication bias strongly suspected^i^ | 287/1219 (23.5%) | 291/2056 (14.2%) | **OR 2.07** (1.71 to 2.51) | **113 more per 1,000** (from 78 more to 151 more) | ⨁◯◯◯ Very low | IMPORTANT |
| **All-cause ICU mortality after redefining exposed cohort by defining FI according to large GRV (as one of the symptoms) of 1000 ml (follow-up: range 20 days to 90+ days; assessed with: medical records)** | | | | | | | | | | | | |
| 1 | observational studies | not serious | not serious | not serious | not serious | publication bias strongly suspected^i^ | 37/128 (28.9%) | 184/1584 (11.6%) | **OR 3.09** (2.05 to 4.67) | **173 more per 1,000** (from 96 more to 264 more) | ⨁◯◯◯ Very low | IMPORTANT |
| **All-cause ICU mortality after redefining exposed cohort by defining FI according to large GRV (as the only symptom) of 250±50 ml (follow-up: range 28 days to 90+ days; assessed with: medical records)** | | | | | | | | | | | | |
| 2 | observational studies | not serious | not serious | not serious | not serious | publication bias strongly suspected^i^ | 133/720 (18.5%) | 94/1051 (8.9%) | **OR 2.31** (0.54 to 9.93) | **95 more per 1,000** (from 39 fewer to 404 more) | ⨁◯◯◯ Very low | IMPORTANT |
| **All-cause ICU mortality after redefining exposed cohort by defining FI according to large GRV (as the only symptom) of 500±50 ml (follow-up: range 28 days to 90+ days; assessed with: medical records)** | | | | | | | | | | | | |
| 2 | observational studies | not serious | not serious | not serious | not serious | publication bias strongly suspected^i^ | 87/384 (22.7%) | 334/2426 (13.8%) | **OR 2.58** (2.08 to 3.19) | **154 more per 1,000** (from 112 more to 200 more) | ⨁◯◯◯ Very low | IMPORTANT |
| **All-cause ICU mortality after redefining exposed cohort by defining FI according to large GRV (as the only symptom) of 1000 ml (follow-up: range 28 days to 90+ days; assessed with: medical records)** | | | | | | | | | | | | |
| 1 | observational studies | not serious | not serious | not serious | not serious | publication bias strongly suspected^i^ | 37/128 (28.9%) | 184/1584 (11.6%) | **OR 3.09** (2.05 to 4.67) | **173 more per 1,000** (from 96 more to 264 more) | ⨁◯◯◯ Very low | IMPORTANT |
| **All-cause ICU mortality after redefining exposed cohort by defining FI according to the measured interval of GRV (as one of the symptoms) of 4 hours (follow-up: range 20 days to 90 days; assessed with: medical records)** | | | | | | | | | | | | |
| 3 | observational studies | not serious | not serious | not serious | not serious | publication bias strongly suspected^i^ | 140/372 (37.6%) | 174/698 (24.9%) | **OR 1.81** (1.32 to 2.48) | **126 more per 1,000** (from 55 more to 202 more) | ⨁◯◯◯ Very low | IMPORTANT |
| **All-cause ICU mortality after redefining exposed cohort by defining FI according to the measured interval of GRV (as one of the symptoms) of 6 hours (follow-up: range 20 days to 90 days; assessed with: medical records)** | | | | | | | | | | | | |
| 1 | observational studies | not serious | not serious | not serious | not serious | publication bias strongly suspected^i^ | 32/162 (19.8%) | 29/293 (9.9%) | **OR 2.24** (1.30 to 3.86) | **98 more per 1,000** (from 26 more to 199 more) | ⨁◯◯◯ Very low | IMPORTANT |
| **All-cause ICU mortality after redefining exposed cohort by defining FI according to the measured interval of GRV (as one of the symptoms) of 24 hours (follow-up: range 20 days to 90 days; assessed with: medical records)** | | | | | | | | | | | | |
| 3 | observational studies | not serious | not serious | not serious | not serious | publication bias strongly suspected^i^ | 135/779 (17.3%) | 298/2226 (13.4%) | **OR 2.20** (1.48 to 3.28) | **120 more per 1,000** (from 52 more to 203 more) | ⨁◯◯◯ Very low | IMPORTANT |
| **All-cause ICU mortality after redefining exposed cohort by defining FI according to the measured interval of GRV (as the only symptom) of 24 hours (follow-up: range 20 days to 90+ days; assessed with: medical records)** | | | | | | | | | | | | |
| 2 | observational studies | not serious | not serious | not serious | not serious | publication bias strongly suspected^i^ | 129/693 (18.6%) | 292/2117 (13.8%) | **OR 2.27** (2.24 to 2.31) | **129 more per 1,000** (from 126 more to 132 more) | ⨁◯◯◯ Very low | IMPORTANT |
| **All-cause ICU mortality after redefining exposed cohort by defining FI according to EF percentage of 20% (follow-up: range 60 days to 90 days; assessed with: medical records)** | | | | | | | | | | | | |
| 1 | observational studies | not serious | not serious | not serious | not serious | publication bias strongly suspected^i^ | 181/989 (18.3%) | 40/723 (5.5%) | **OR 3.82** (2.68 to 5.47) | **127 more per 1,000** (from 80 more to 187 more) | ⨁◯◯◯ Very low | IMPORTANT |
| **All-cause ICU mortality after redefining exposed cohort by defining FI according to EF percentage of 50% (follow-up: range 60 days to 90 days; assessed with: medical records)** | | | | | | | | | | | | |
| 1 | observational studies | not serious | not serious | not serious | not serious | publication bias strongly suspected^i^ | 213/1459 (14.6%) | 8/253 (3.2%) | **OR 5.24** (2.55 to 10.74) | **114 more per 1,000** (from 45 more to 228 more) | ⨁◯◯◯ Very low | IMPORTANT |
| **All-cause ICU mortality after redefining exposed cohort by defining FI according to EF percentage of 80% (follow-up: range 60 days to 90 days; assessed with: medical records)** | | | | | | | | | | | | |
| 3 | observational studies | not serious | not serious | not serious | serious^m^ | publication bias strongly suspected^i^ | 224/1199 (18.7%) | 160/760 (21.1%) | **OR 1.87** (1.08 to 3.24) | **122 more per 1,000** (from 13 more to 253 more) | ⨁◯◯◯ Very low | IMPORTANT |
| **All-cause ICU mortality in the study population with surgical patients accounted for 0% (follow-up: range 20 days to 90+ days; assessed with: medical records)** | | | | | | | | | | | | |
| 4 | observational studies | serious^k^ | not serious | not serious | not serious | publication bias strongly suspected^i^ | 24/168 (14.3%) | 28/256 (10.9%) | **OR 1.57** (0.33 to 7.61) | **52 more per 1,000** (from 70 fewer to 374 more) | ⨁◯◯◯ Very low | IMPORTANT |
| **All-cause ICU mortality in the study population with surgical patients accounted for 15-20% (follow-up: range 20 days to 90+ days; assessed with: medical records)** | | | | | | | | | | | | |
| 1 | observational studies | not serious | not serious | not serious | serious^m^ | publication bias strongly suspected^i^ | 70/196 (35.7%) | 77/303 (25.4%) | **OR 1.63** (1.10 to 2.41) | **103 more per 1,000** (from 18 more to 197 more) | ⨁◯◯◯ Very low | IMPORTANT |
| **All-cause ICU mortality in the study population with surgical patients accounted for >25% (follow-up: range 20 days to 90+ days; assessed with: medical records)** | | | | | | | | | | | | |
| 2 | observational studies | not serious | not serious | not serious | not serious | publication bias strongly suspected^i^ | 61/232 (26.3%) | 50/376 (13.3%) | **OR 2.18** (1.41 to 3.37) | **118 more per 1,000** (from 45 more to 208 more) | ⨁◯◯◯ Very low | IMPORTANT |
| **All-cause ICU mortality in the study population with male patients accounted for ≤65% (follow-up: range 20 days to 90+ days; assessed with: medical records)** | | | | | | | | | | | | |
| 7 | observational studies | not serious | not serious | not serious | not serious | publication bias strongly suspected^i^ | 487/2854 (17.1%) | 230/2378 (9.7%) | **OR 2.29** (1.86 to 2.83) | **100 more per 1,000** (from 69 more to 136 more) | ⨁◯◯◯ Very low | IMPORTANT |
| **All-cause ICU mortality in the study population with male patients accounted for >65% (follow-up: range 20 days to 90+ days; assessed with: medical records)** | | | | | | | | | | | | |
| 8 | observational studies | not serious | not serious | not serious | not serious | publication bias strongly suspected^i^ | 250/885 (28.2%) | 475/2660 (17.9%) | **OR 2.01** (1.63 to 2.49) | **126 more per 1,000** (from 83 more to 173 more) | ⨁◯◯◯ Very low | IMPORTANT |
| **All-cause ICU mortality in the study population with an average age of >55 (follow-up: range 20 days to 90+ days; assessed with: medical records)** | | | | | | | | | | | | |
| 13 | observational studies | not serious | not serious | not serious | not serious | none | 722/3693 (19.6%) | 697/4979 (14.0%) | **OR 2.14** (1.88 to 2.43) | **118 more per 1,000** (from 94 more to 143 more) | ⨁⨁◯◯ Low | IMPORTANT |
| **All-cause ICU mortality in the study population with an average age of ≤55 (follow-up: range 20 days to 90+ days; assessed with: medical records)** | | | | | | | | | | | | |
| 2 | observational studies | serious^k^ | not serious | not serious | not serious | publication bias strongly suspected^i^ | 15/46 (32.6%) | 8/59 (13.6%) | **OR 5.51** (4.86 to 6.24) | **328 more per 1,000** (from 297 more to 359 more) | ⨁◯◯◯ Very low | IMPORTANT |
| **All-cause ICU mortality in the study population with trauma patients accounted for <25% (follow-up: range 20 days to 90+ days; assessed with: medical records)** | | | | | | | | | | | | |
| 2 | observational studies | not serious | serious^l^ | not serious | extremely serious^n^ | publication bias strongly suspected^i^ | 31/87 (35.6%) | 27/104 (26.0%) | **OR 1.00** (0.00 to 91372.87) | **0 fewer per 1,000** (from -- to 740 more) | ⨁◯◯◯ Very low | IMPORTANT |
| **All-cause ICU mortality in the study population with burn patients accounted for 100% (follow-up: range 20 days to 90+ days; assessed with: medical records)** | | | | | | | | | | | | |
| 2 | observational studies | serious^k^ | not serious | not serious | not serious | publication bias strongly suspected^i^ | 15/46 (32.6%) | 8/59 (13.6%) | **OR 5.51** (4.86 to 6.24) | **328 more per 1,000** (from 297 more to 359 more) | ⨁◯◯◯ Very low | IMPORTANT |
| **All-cause ICU mortality in the study population with abdominal surgery patients accounted for 15% (follow-up: range 20 days to 90+ days; assessed with: medical records)** | | | | | | | | | | | | |
| 2 | observational studies | not serious | not serious | not serious | not serious | publication bias strongly suspected^i^ | 61/323 (18.9%) | 50/376 (13.3%) | **OR 2.18** (1.41 to 3.37) | **118 more per 1,000** (from 45 more to 208 more) | ⨁◯◯◯ Very low | IMPORTANT |
| **All-cause ICU mortality in the study population with digestive diseases patients accounted for <10% (follow-up: range 20 days to 90+ days; assessed with: medical records)** | | | | | | | | | | | | |
| 2 | observational studies | not serious | not serious | not serious | not serious | publication bias strongly suspected^i^ | 61/323 (18.9%) | 50/376 (13.3%) | **OR 2.18** (1.41 to 3.37) | **118 more per 1,000** (from 45 more to 208 more) | ⨁◯◯◯ Very low | IMPORTANT |
| **All-cause ICU mortality in the study population with sepsis patients accounted for 100% (follow-up: range 20 days to 90+ days; assessed with: medical records)** | | | | | | | | | | | | |
| 4 | observational studies | serious^k^ | not serious | not serious | not serious | publication bias strongly suspected^i^ | 47/183 (25.7%) | 44/310 (14.2%) | **OR 2.58** (1.22 to 5.50) | **157 more per 1,000** (from 26 more to 334 more) | ⨁◯◯◯ Very low | IMPORTANT |
| **All-cause ICU mortality in the study population with sepsis patients accounted for <25% (follow-up: range 20 days to 90+ days; assessed with: medical records)** | | | | | | | | | | | | |
| 12 | observational studies | not serious | not serious | not serious | not serious | none | 452/1693 (26.7%) | 1809/10840 (16.7%) | **OR 2.19** (1.94 to 2.46) | **138 more per 1,000** (from 113 more to 163 more) | ⨁⨁◯◯ Low | IMPORTANT |
| **All-cause ICU mortality in the study population with sepsis patients accounted for 25-55% (follow-up: range 20 days to 90+ days; assessed with: medical records)** | | | | | | | | | | | | |
| 2 | observational studies | not serious | serious^l^ | not serious | extremely serious^n^ | publication bias strongly suspected^i^ | 72/213 (33.8%) | 83/324 (25.6%) | **OR 0.94** (0.00 to 14053.02) | **12 fewer per 1,000** (from -- to 744 more) | ⨁◯◯◯ Very low | IMPORTANT |
| **All-cause ICU mortality in the study population with mechanical ventilation patients accounted for 75-95% (follow-up: range 20 days to 90+ days; assessed with: medical records)** | | | | | | | | | | | | |
| 20 | observational studies | not serious | not serious | not serious | not serious | none | 1756/9195 (19.1%) | 2351/22234 (10.6%) | **OR 2.65** (2.27 to 3.10) | **133 more per 1,000** (from 106 more to 162 more) | ⨁⨁◯◯ Low | IMPORTANT |
| **All-cause ICU mortality in the study population with mechanical ventilation patients accounted for 100% (follow-up: range 20 days to 90+ days; assessed with: medical records)** | | | | | | | | | | | | |
| 2 | observational studies | serious^k^ | not serious | not serious | very serious^o^ | publication bias strongly suspected^i^ | 25/76 (32.9%) | 17/103 (16.5%) | **OR 3.02** (0.18 to 51.89) | **209 more per 1,000** (from 131 fewer to 746 more) | ⨁◯◯◯ Very low | IMPORTANT |
| **All-cause ICU mortality in the study population with mechanical ventilation patients accounted for <30% (follow-up: range 20 days to 90+ days; assessed with: medical records)** | | | | | | | | | | | | |
| 11 | observational studies | not serious | not serious | not serious | not serious | none | 420/1531 (27.4%) | 1780/10547 (16.9%) | **OR 2.18** (1.91 to 2.49) | **138 more per 1,000** (from 111 more to 167 more) | ⨁⨁◯◯ Low | IMPORTANT |
| **All-cause ICU mortality in the study population with average SOFA score of <8 (follow-up: range 20 days to 90+ days; assessed with: medical records)** | | | | | | | | | | | | |
| 12 | observational studies | not serious | not serious | not serious | not serious | none | 430/1547 (27.8%) | 1787/10577 (16.9%) | **OR 2.20** (1.92 to 2.53) | **140 more per 1,000** (from 112 more to 171 more) | ⨁⨁◯◯ Low | IMPORTANT |
| **All-cause ICU mortality in the study population with average SOFA score of ≥8 (follow-up: range 20 days to 90+ days; assessed with: medical records)** | | | | | | | | | | | | |
| 21 | observational studies | not serious | not serious | not serious | not serious | none | 1792/9251 (19.4%) | 2367/22234 (10.6%) | **OR 2.62** (2.21 to 3.10) | **131 more per 1,000** (from 102 more to 163 more) | ⨁⨁◯◯ Low | IMPORTANT |
| **All-cause ICU mortality in the study population with average APACHE II score of <20 (follow-up: range 20 days to 90+ days; assessed with: medical records)** | | | | | | | | | | | | |
| 35^d^ | observational studies | not serious | not serious | not serious | not serious | none | 2268/10934 (20.7%) | 4231/33152 (12.8%) | **OR 2.48** (2.20 to 2.79) | **139 more per 1,000** (from 116 more to 162 more) | ⨁⨁◯◯ Low | IMPORTANT |
| **All-cause ICU mortality in the study population with average APACHE II score of ≥20 (follow-up: range 20 days to 90+ days; assessed with: medical records)** | | | | | | | | | | | | |
| 1 | observational studies | serious^k^ | not serious | not serious | not serious | publication bias strongly suspected^i^ | 20/46 (43.5%) | 16/74 (21.6%) | **OR 2.79** (1.25 to 6.23) | **219 more per 1,000** (from 40 more to 416 more) | ⨁◯◯◯ Very low | IMPORTANT |
| **All-cause hospital mortality after redefining exposed cohort by defining FI according to GI symptoms cluster (follow-up: range 13 days to 150+ days; assessed with: medical records)** | | | | | | | | | | | | |
| 11 | observational studies | not serious | serious^l^ | not serious | not serious | none | 1590/5298 (30.0%) | 3267/13905 (23.5%) | **OR 1.48** (0.88 to 2.50) | **78 more per 1,000** (from 22 fewer to 199 more) | ⨁◯◯◯ Very low | CRITICAL |
| **All-cause hospital mortality after redefining exposed cohort by defining FI according to large-GRV-containing GI symptoms cluster (follow-up: range 13 days to 150+ days; assessed with: medical records)** | | | | | | | | | | | | |
| 6 | observational studies | not serious | not serious | not serious | serious^m^ | publication bias strongly suspected^i^ | 1497/4939 (30.3%) | 3207/13524 (23.7%) | **OR 1.42** (1.10 to 1.83) | **69 more per 1,000** (from 18 more to 125 more) | ⨁◯◯◯ Very low | CRITICAL |
| **All-cause hospital mortality after redefining exposed cohort by defining FI according to only large GRV (follow-up: range 13 days to 150+ days; assessed with: medical records)** | | | | | | | | | | | | |
| 4 | observational studies | not serious | serious^l^ | not serious | serious^h^ | publication bias strongly suspected^i^ | 32/179 (17.9%) | 37/238 (15.5%) | **OR 1.07** (0.08 to 15.20) | **9 more per 1,000** (from 141 fewer to 581 more) | ⨁◯◯◯ Very low | CRITICAL |
| **All-cause hospital mortality after redefining exposed cohort by defining FI according to EF insufficiency (follow-up: range 13 days to 150+ days; assessed with: medical records)** | | | | | | | | | | | | |
| 5 | observational studies | not serious | not serious | not serious | serious^m^ | publication bias strongly suspected^i^ | 209/510 (41.0%) | 223/843 (26.5%) | **OR 1.90** (1.03 to 3.50) | **141 more per 1,000** (from 6 more to 293 more) | ⨁◯◯◯ Very low | CRITICAL |
| **All-cause hospital mortality after redefining exposed cohort by defining FI according to GI symptoms cluster without large GRV (follow-up: range 13 days to 150+ days; assessed with: medical records)** | | | | | | | | | | | | |
| 1 | observational studies | not serious | not serious | not serious | not serious | publication bias strongly suspected^i^ | 61/180 (33.9%) | 23/143 (16.1%) | **OR 2.67** (1.55 to 4.60) | **178 more per 1,000** (from 68 more to 308 more) | ⨁◯◯◯ Very low | CRITICAL |
| **All-cause hospital mortality after redefining exposed cohort by defining FI according to the number of GI symptoms ≥1 (no limit to the total number of candidate symptoms, which can range from 1-6) (follow-up: range 13 days to 150+ days; assessed with: medical records)** | | | | | | | | | | | | |
| 5 | observational studies | not serious | not serious | not serious | serious^m^ | publication bias strongly suspected^i^ | 1493/4880 (30.6%) | 3193/13436 (23.8%) | **OR 1.44** (1.19 to 1.74) | **72 more per 1,000** (from 33 more to 114 more) | ⨁◯◯◯ Very low | IMPORTANT |
| **All-cause hospital mortality after redefining exposed cohort by defining FI according to the number of GI symptoms ≥2 (no limit to the total number of candidate symptoms, which can range from 1-6) (follow-up: range 13 days to 150+ days; assessed with: medical records)** | | | | | | | | | | | | |
| 1 | observational studies | serious^k^ | not serious | not serious | not serious | publication bias strongly suspected^i^ | 4/59 (6.8%) | 14/88 (15.9%) | **OR 0.38** (0.12 to 1.23) | **92 fewer per 1,000** (from 137 fewer to 30 more) | ⨁◯◯◯ Very low | IMPORTANT |
| **All-cause hospital mortality after redefining exposed cohort by defining FI according to the number of GI symptoms ≥1 (limit the total number of candidate symptoms to 4) (follow-up: range 13 days to 150+ days; assessed with: medical records)** | | | | | | | | | | | | |
| 2 | observational studies | not serious | not serious | not serious | serious^m^ | publication bias strongly suspected^i^ | 1445/4669 (30.9%) | 3350/14165 (23.6%) | **OR 1.45** (1.15 to 1.82) | **73 more per 1,000** (from 26 more to 124 more) | ⨁◯◯◯ Very low | IMPORTANT |
| **All-cause hospital mortality after redefining exposed cohort by defining FI according to the number of GI symptoms ≥1 (limit the total number of candidate symptoms to 5) (follow-up: range 13 days to 150+ days; assessed with: medical records)** | | | | | | | | | | | | |
| 7 | observational studies | not serious | not serious | not serious | not serious | publication bias strongly suspected^i^ | 573/1799 (31.9%) | 821/3224 (25.5%) | **OR 1.78** (1.22 to 2.60) | **124 more per 1,000** (from 40 more to 216 more) | ⨁◯◯◯ Very low | IMPORTANT |
| **All-cause hospital mortality after redefining exposed cohort by defining FI according to the number of GI symptoms ≥2 (limit the total number of candidate symptoms to 5) (follow-up: range 13 days to 150+ days; assessed with: medical records)** | | | | | | | | | | | | |
| 1 | observational studies | serious^k^ | not serious | not serious | not serious | publication bias strongly suspected^i^ | 4/59 (6.8%) | 14/88 (15.9%) | **OR 0.38** (0.12 to 1.23) | **92 fewer per 1,000** (from 137 fewer to 30 more) | ⨁◯◯◯ Very low | IMPORTANT |
| **All-cause hospital mortality after redefining exposed cohort by defining FI according to large GRV (as one of the symptoms) of 500±50 ml (follow-up: range 13 days to 150+ days; assessed with: medical records)** | | | | | | | | | | | | |
| 3 | observational studies | not serious | not serious | not serious | not serious | publication bias strongly suspected^i^ | 66/183 (36.1%) | 61/237 (25.7%) | **OR 1.65** (0.27 to 9.98) | **106 more per 1,000** (from 172 fewer to 518 more) | ⨁◯◯◯ Very low | IMPORTANT |
| **All-cause hospital mortality after redefining exposed cohort by defining FI according to large GRV (as one of the symptoms) of 75±50 ml (follow-up: range 13 days to 150+ days; assessed with: medical records)** | | | | | | | | | | | | |
| 1 | observational studies | serious^k^ | not serious | not serious | not serious | publication bias strongly suspected^i^ | 6/29 (20.7%) | 12/32 (37.5%) | **OR 0.43** (0.14 to 1.37) | **170 fewer per 1,000** (from 298 fewer to 76 more) | ⨁◯◯◯ Very low | IMPORTANT |
| **All-cause hospital mortality after redefining exposed cohort by defining FI according to large GRV (as one of the symptoms) of 250±50 ml (follow-up: range 13 days to 150+ days; assessed with: medical records)** | | | | | | | | | | | | |
| 10 | observational studies | not serious | not serious | not serious | not serious | none | 618/1960 (31.5%) | 1057/4372 (24.2%) | **OR 1.90** (1.40 to 2.57) | **136 more per 1,000** (from 67 more to 209 more) | ⨁⨁◯◯ Low | IMPORTANT |
| **All-cause hospital mortality after redefining exposed cohort by defining FI according to large GRV (as the only symptom) of 75±50 ml (follow-up: range 13 days to 150+ days; assessed with: medical records)** | | | | | | | | | | | | |
| 1 | observational studies | serious^k^ | not serious | not serious | not serious | publication bias strongly suspected^i^ | 6/29 (20.7%) | 12/32 (37.5%) | **OR 0.43** (0.14 to 1.37) | **170 fewer per 1,000** (from 298 fewer to 76 more) | ⨁◯◯◯ Very low | IMPORTANT |
| **All-cause hospital mortality after redefining exposed cohort by defining FI according to large GRV (as the only symptom) of 500±50 ml (follow-up: range 13 days to 150+ days; assessed with: medical records)** | | | | | | | | | | | | |
| 1 | observational studies | serious^k^ | not serious | not serious | not serious | publication bias strongly suspected^i^ | 7/67 (10.4%) | 11/80 (13.8%) | **OR 0.73** (0.27 to 2.01) | **33 fewer per 1,000** (from 96 fewer to 105 more) | ⨁◯◯◯ Very low | IMPORTANT |
| **All-cause hospital mortality after redefining exposed cohort by defining FI according to large GRV (as the only symptom) of 250±50 ml (follow-up: range 13 days to 150+ days; assessed with: medical records)** | | | | | | | | | | | | |
| 2 | observational studies | not serious | not serious | not serious | not serious | publication bias strongly suspected^i^ | 19/83 (22.9%) | 14/126 (11.1%) | **OR 3.31** (1.49 to 7.35) | **182 more per 1,000** (from 46 more to 368 more) | ⨁◯◯◯ Very low | IMPORTANT |
| **All-cause hospital mortality after redefining exposed cohort by defining FI according to the measured interval of GRV (as one of the symptoms) of 4 hours (follow-up: range 13 days to 150+ days; assessed with: medical records)** | | | | | | | | | | | | |
| 7 | observational studies | not serious | not serious | not serious | not serious | publication bias strongly suspected^i^ | 265/659 (40.2%) | 267/986 (27.1%) | **OR 171.00** (0.93 to 3.15) | **714 more per 1,000** (from 14 fewer to 268 more) | ⨁◯◯◯ Very low | IMPORTANT |
| **All-cause hospital mortality after redefining exposed cohort by defining FI according to the measured interval of GRV (as one of the symptoms) of 6 hours (follow-up: range 13 days to 150+ days; assessed with: medical records)** | | | | | | | | | | | | |
| 1 | observational studies | serious^k^ | not serious | not serious | not serious | publication bias strongly suspected^i^ | 9/21 (42.9%) | 14/51 (27.5%) | **OR 1.98** (0.69 to 5.73) | **154 more per 1,000** (from 67 fewer to 410 more) | ⨁◯◯◯ Very low | IMPORTANT |
| **All-cause hospital mortality after redefining exposed cohort by defining FI according to the measured interval of GRV (as the only symptom) of 4 hours (follow-up: range 13 days to 150+ days; assessed with: medical records)** | | | | | | | | | | | | |
| 3 | observational studies | not serious | serious^l^ | not serious | serious^h^ | publication bias strongly suspected^i^ | 32/149 (21.5%) | 37/209 (17.7%) | **OR 1.07** (0.08 to 15.20) | **10 more per 1,000** (from 160 fewer to 589 more) | ⨁◯◯◯ Very low | IMPORTANT |
| **All-cause hospital mortality after redefining exposed cohort by defining FI according to EF percentage of 80% (follow-up: range 13 days to 150+ days; assessed with: medical records)** | | | | | | | | | | | | |
| 5 | observational studies | not serious | not serious | not serious | serious^m^ | publication bias strongly suspected^i^ | 209/510 (41.0%) | 223/843 (26.5%) | **OR 1.90** (1.03 to 3.50) | **141 more per 1,000** (from 6 more to 293 more) | ⨁◯◯◯ Very low | IMPORTANT |
| **All-cause hospital mortality in the study population with surgical patients accounted for >25% (follow-up: range 13 days to 150+ days; assessed with: medical records)** | | | | | | | | | | | | |
| 4 | observational studies | not serious | not serious | not serious | not serious | publication bias strongly suspected^i^ | 1303/4279 (30.5%) | 2847/12101 (23.5%) | **OR 1.46** (1.36 to 1.58) | **75 more per 1,000** (from 60 more to 92 more) | ⨁◯◯◯ Very low | IMPORTANT |
| **All-cause hospital mortality in the study population with surgical patients accounted for 0% (follow-up: range 13 days to 150+ days; assessed with: medical records)** | | | | | | | | | | | | |
| 8 | observational studies | not serious | serious^l^ | not serious | not serious | publication bias strongly suspected^i^ | 171/605 (28.3%) | 105/646 (16.3%) | **OR 1.24** (0.49 to 3.10) | **31 more per 1,000** (from 76 fewer to 213 more) | ⨁◯◯◯ Very low | IMPORTANT |
| **All-cause hospital mortality in the study population with surgical patients accounted for 15-20% (follow-up: range 13 days to 150+ days; assessed with: medical records)** | | | | | | | | | | | | |
| 2 | observational studies | not serious | not serious | not serious | not serious | publication bias strongly suspected^i^ | 256/772 (33.2%) | 430/1615 (26.6%) | **OR 1.40** (0.21 to 9.43) | **71 more per 1,000** (from 195 fewer to 508 more) | ⨁◯◯◯ Very low | IMPORTANT |
| **All-cause hospital mortality in the study population with male patients accounted for >65% (follow-up: range 13 days to 150+ days; assessed with: medical records)** | | | | | | | | | | | | |
| 7 | observational studies | not serious | serious^l^ | not serious | not serious | publication bias strongly suspected^i^ | 1573/5113 (30.8%) | 3230/13676 (23.6%) | **OR 1.89** (1.28 to 2.80) | **133 more per 1,000** (from 47 more to 228 more) | ⨁◯◯◯ Very low | IMPORTANT |
| **All-cause hospital mortality in the study population with male patients accounted for ≤65% (follow-up: range 13 days to 150+ days; assessed with: medical records)** | | | | | | | | | | | | |
| 9 | observational studies | not serious | not serious | not serious | not serious | publication bias strongly suspected^i^ | 226/695 (32.5%) | 260/1072 (24.3%) | **OR 1.20** (0.61 to 2.35) | **35 more per 1,000** (from 79 fewer to 187 more) | ⨁◯◯◯ Very low | IMPORTANT |
| **All-cause hospital mortality in the study population with an average age of >55 (follow-up: range 13 days to 150+ days; assessed with: medical records)** | | | | | | | | | | | | |
| 10 | observational studies | not serious | not serious | not serious | not serious | none | 1772/5422 (32.7%) | 3439/14336 (24.0%) | **OR 1.92** (1.47 to 2.51) | **137 more per 1,000** (from 77 more to 202 more) | ⨁⨁◯◯ Low | IMPORTANT |
| **All-cause hospital mortality in the study population with an average age of ≤55 (follow-up: range 13 days to 150+ days; assessed with: medical records)** | | | | | | | | | | | | |
| 6 | observational studies | serious^k^ | not serious | not serious | not serious | publication bias strongly suspected^i^ | 27/386 (7.0%) | 51/412 (12.4%) | **OR 0.60** (0.29 to 1.24) | **46 fewer per 1,000** (from 84 fewer to 25 more) | ⨁◯◯◯ Very low | IMPORTANT |
| **All-cause hospital mortality in the study population with trauma patients accounted for <25% (follow-up: range 13 days to 150+ days; assessed with: medical records)** | | | | | | | | | | | | |
| 3 | observational studies | not serious | not serious | not serious | not serious | publication bias strongly suspected^i^ | 233/699 (33.3%) | 392/1492 (26.3%) | **OR 1.69** (0.52 to 5.53) | **113 more per 1,000** (from 106 fewer to 401 more) | ⨁◯◯◯ Very low | IMPORTANT |
| **All-cause hospital mortality in the study population with trauma patients accounted for 40-60% (follow-up: range 13 days to 150+ days; assessed with: medical records)** | | | | | | | | | | | | |
| 1 | observational studies | serious^k^ | not serious | not serious | not serious | publication bias strongly suspected^i^ | 9/21 (42.9%) | 14/51 (27.5%) | **OR 1.98** (0.69 to 5.73) | **154 more per 1,000** (from 67 fewer to 410 more) | ⨁◯◯◯ Very low | IMPORTANT |
| **All-cause hospital mortality in the study population with trauma patients accounted for 100% (follow-up: range 13 days to 150+ days; assessed with: medical records)** | | | | | | | | | | | | |
| 3 | observational studies | serious^k^ | not serious | not serious | not serious | publication bias strongly suspected^i^ | 15/175 (8.6%) | 38/266 (14.3%) | **OR 0.56** (0.25 to 1.27) | **57 fewer per 1,000** (from 103 fewer to 32 more) | ⨁◯◯◯ Very low | IMPORTANT |
| **All-cause hospital mortality in the study population with abdominal surgery patients accounted for 15% (follow-up: range 13 days to 150+ days; assessed with: medical records)** | | | | | | | | | | | | |
| 2 | observational studies | not serious | not serious | not serious | not serious | publication bias strongly suspected^i^ | 46/91 (50.5%) | 48/134 (35.8%) | **OR 1.71** (0.54 to 5.39) | **130 more per 1,000** (from 127 fewer to 392 more) | ⨁◯◯◯ Very low | IMPORTANT |
| **All-cause hospital mortality in the study population with abdominal surgery patients accounted for 5% (follow-up: range 13 days to 150+ days; assessed with: medical records)** | | | | | | | | | | | | |
| 1 | observational studies | not serious | not serious | not serious | serious^m^ | publication bias strongly suspected^i^ | 177/576 (30.7%) | 344/1312 (26.2%) | **OR 1.25** (1.01 to 1.55) | **45 more per 1,000** (from 2 more to 93 more) | ⨁◯◯◯ Very low | IMPORTANT |
| **All-cause hospital mortality in the study population with digestive diseases patients accounted for <10% (follow-up: range 13 days to 150+ days; assessed with: medical records)** | | | | | | | | | | | | |
| 2 | observational studies | not serious | not serious | not serious | not serious | publication bias strongly suspected^i^ | 61/232 (26.3%) | 50/376 (13.3%) | **OR 2.18** (1.41 to 3.37) | **118 more per 1,000** (from 45 more to 208 more) | ⨁◯◯◯ Very low | IMPORTANT |
| **All-cause hospital mortality in the study population with sepsis patients accounted for <25% (follow-up: range 13 days to 150+ days; assessed with: medical records)** | | | | | | | | | | | | |
| 3 | observational studies | not serious | not serious | not serious | not serious | publication bias strongly suspected^i^ | 205/650 (31.5%) | 372/1460 (25.5%) | **OR 1.83** (0.51 to 6.54) | **130 more per 1,000** (from 106 fewer to 436 more) | ⨁◯◯◯ Very low | IMPORTANT |
| **All-cause hospital mortality in the study population with sepsis patients accounted for 100% (follow-up: range 13 days to 150+ days; assessed with: medical records)** | | | | | | | | | | | | |
| 1 | observational studies | serious^k^ | not serious | not serious | not serious | publication bias strongly suspected^i^ | 22/46 (47.8%) | 16/74 (21.6%) | **OR 3.32** (1.49 to 7.40) | **262 more per 1,000** (from 75 more to 455 more) | ⨁◯◯◯ Very low | IMPORTANT |
| **All-cause hospital mortality in the study population with sepsis patients accounted for 25-55% (follow-up: range 13 days to 150+ days; assessed with: medical records)** | | | | | | | | | | | | |
| 1 | observational studies | not serious | not serious | not serious | serious^m^ | publication bias strongly suspected^i^ | 79/196 (40.3%) | 86/303 (28.4%) | **OR 1.70** (1.17 to 2.49) | **119 more per 1,000** (from 33 more to 213 more) | ⨁◯◯◯ Very low | IMPORTANT |
| **All-cause hospital mortality in the study population with mechanical ventilation patients accounted for 75-95% (follow-up: range 13 days to 150+ days; assessed with: medical records)** | | | | | | | | | | | | |
| 2 | observational studies | not serious | not serious | not serious | not serious | publication bias strongly suspected^i^ | 56/127 (44.1%) | 106/363 (29.2%) | **OR 1.92** (1.60 to 2.30) | **150 more per 1,000** (from 106 more to 195 more) | ⨁◯◯◯ Very low | IMPORTANT |
| **All-cause hospital mortality in the study population with mechanical ventilation patients accounted for 100% (follow-up: range 13 days to 150+ days; assessed with: medical records)** | | | | | | | | | | | | |
| 5 | observational studies | not serious | not serious | not serious | not serious | publication bias strongly suspected^i^ | 1469/4741 (31.0%) | 3172/13394 (23.7%) | **OR 1.84** (0.83 to 4.07) | **127 more per 1,000** (from 32 fewer to 321 more) | ⨁◯◯◯ Very low | IMPORTANT |
| **All-cause hospital mortality in the study population with average SOFA score of <8 (follow-up: range 13 days to 150+ days; assessed with: medical records)** | | | | | | | | | | | | |
| 3 | observational studies | not serious | not serious | not serious | not serious | publication bias strongly suspected^i^ | 140/339 (41.3%) | 55/273 (20.1%) | **OR 2.84** (1.62 to 5.00) | **216 more per 1,000** (from 89 more to 356 more) | ⨁◯◯◯ Very low | IMPORTANT |
| **All-cause hospital mortality in the study population with average SOFA score of ≥8 (follow-up: range 13 days to 150+ days; assessed with: medical records)** | | | | | | | | | | | | |
| 3 | observational studies | not serious | not serious | not serious | not serious | publication bias strongly suspected^i^ | 104/401 (25.9%) | 101/485 (20.8%) | **OR 2.20** (0.82 to 5.95) | **158 more per 1,000** (from 31 fewer to 402 more) | ⨁◯◯◯ Very low | IMPORTANT |
| **All-cause hospital mortality in the study population with average APACHE II score of <20 (follow-up: range 13 days to 150+ days; assessed with: medical records)** | | | | | | | | | | | | |
| 4 | observational studies | not serious | not serious | not serious | not serious | publication bias strongly suspected^i^ | 135/353 (38.2%) | 192/695 (27.6%) | **OR 1.80** (1.50 to 2.15) | **131 more per 1,000** (from 88 more to 174 more) | ⨁◯◯◯ Very low | IMPORTANT |
| **All-cause hospital mortality in the study population with average APACHE II score of ≥20 (follow-up: range 13 days to 150+ days; assessed with: medical records)** | | | | | | | | | | | | |
| 4 | observational studies | not serious | not serious | not serious | not serious | publication bias strongly suspected^i^ | 1469/4711 (31.2%) | 3172/13365 (23.7%) | **OR 1.84** (0.83 to 4.07) | **127 more per 1,000** (from 32 fewer to 321 more) | ⨁◯◯◯ Very low | IMPORTANT |
| **All-cause long-term mortality after redefining exposed cohort by defining FI according to GI symptoms cluster (follow-up: range 90 days to 150+ days; assessed with: follow-up records)** | | | | | | | | | | | | |
| 3 | observational studies | not serious | not serious | not serious | not serious | publication bias strongly suspected^i^ | 684/2094 (32.7%) | 801/3042 (26.3%) | **OR 1.36** (0.60 to 3.08) | **64 more per 1,000** (from 87 fewer to 261 more) | ⨁◯◯◯ Very low | CRITICAL |
| **All-cause long-term mortality after redefining exposed cohort by defining FI according to only large GRV (follow-up: range 90 days to 150+ days; assessed with: follow-up records)** | | | | | | | | | | | | |
| 1 | observational studies | not serious | not serious | not serious | not serious | publication bias strongly suspected^i^ | 237/690 (34.3%) | 258/1022 (25.2%) | **OR 1.55** (1.25 to 1.91) | **91 more per 1,000** (from 44 more to 140 more) | ⨁◯◯◯ Very low | CRITICAL |
| **All-cause long-term mortality after redefining exposed cohort by defining FI according to EF insufficiency (follow-up: range 90 days to 150+ days; assessed with: follow-up records)** | | | | | | | | | | | | |
| 2 | observational studies | not serious | not serious | not serious | extremely serious^n^ | publication bias strongly suspected^i^ | 350/1035 (33.8%) | 51/224 (22.8%) | **OR 2.34** (0.01 to 383.22) | **181 more per 1,000** (from 225 fewer to 764 more) | ⨁◯◯◯ Very low | CRITICAL |
| **All-cause long-term mortality after redefining exposed cohort by defining FI according to large-GRV-containing GI symptoms cluster (follow-up: range 90 days to 150+ days; assessed with: follow-up records)** | | | | | | | | | | | | |
| 1 | observational studies | not serious | not serious | not serious | not serious | publication bias strongly suspected^i^ | 327/972 (33.6%) | 168/740 (22.7%) | **OR 1.73** (1.39 to 2.15) | **110 more per 1,000** (from 63 more to 160 more) | ⨁◯◯◯ Very low | CRITICAL |
| **All-cause long-term mortality after redefining exposed cohort by defining FI according to GI symptoms cluster without large GRV (follow-up: range 90 days to 150+ days; assessed with: follow-up records)** | | | | | | | | | | | | |
| 1 | observational studies | not serious | not serious | not serious | not serious | publication bias strongly suspected^i^ | 120/432 (27.8%) | 375/1280 (29.3%) | **OR 0.93** (0.73 to 1.18) | **15 fewer per 1,000** (from 61 fewer to 35 more) | ⨁◯◯◯ Very low | CRITICAL |
| **All-cause long-term mortality after redefining exposed cohort by defining FI according to the number of GI symptoms ≥1 (no limit to the total number of candidate symptoms, which can range from 1-6) (follow-up: range 90 days to 150+ days; assessed with: follow-up records)** | | | | | | | | | | | | |
| 1 | observational studies | not serious | not serious | not serious | not serious | publication bias strongly suspected^i^ | 327/972 (33.6%) | 168/740 (22.7%) | **OR 1.73** (1.39 to 2.15) | **110 more per 1,000** (from 63 more to 160 more) | ⨁◯◯◯ Very low | IMPORTANT |
| **All-cause long-term mortality after redefining exposed cohort by defining FI according to the number of GI symptoms ≥2 (no limit to the total number of candidate symptoms, which can range from 1-6) (follow-up: range 90 days to 150+ days; assessed with: follow-up records)** | | | | | | | | | | | | |
| 1 | observational studies | not serious | not serious | not serious | not serious | publication bias strongly suspected^i^ | 197/520 (37.9%) | 298/1192 (25.0%) | **OR 1.83** (1.47 to 2.28) | **129 more per 1,000** (from 79 more to 182 more) | ⨁◯◯◯ Very low | IMPORTANT |
| **All-cause long-term mortality after redefining exposed cohort by defining FI according to the number of GI symptoms ≥3 (no limit to the total number of candidate symptoms, which can range from 1-6) (follow-up: range 90 days to 150+ days; assessed with: follow-up records)** | | | | | | | | | | | | |
| 1 | observational studies | not serious | not serious | not serious | not serious | publication bias strongly suspected^i^ | 74/146 (50.7%) | 421/1566 (26.9%) | **OR 2.80** (1.98 to 3.94) | **238 more per 1,000** (from 152 more to 323 more) | ⨁◯◯◯ Very low | IMPORTANT |
| **All-cause long-term mortality after redefining exposed cohort by defining FI according to the number of GI symptoms ≥1 (limit the total number of candidate symptoms to 4) (follow-up: range 90 days to 150+ days; assessed with: follow-up records)** | | | | | | | | | | | | |
| 1 | observational studies | not serious | not serious | not serious | not serious | publication bias strongly suspected^i^ | 298/848 (35.1%) | 197/864 (22.8%) | **OR 1.83** (1.48 to 2.27) | **123 more per 1,000** (from 76 more to 173 more) | ⨁◯◯◯ Very low | IMPORTANT |
| **All-cause long-term mortality after redefining exposed cohort by defining FI according to the number of GI symptoms ≥2 (limit the total number of candidate symptoms to 4) (follow-up: range 90 days to 150+ days; assessed with: follow-up records)** | | | | | | | | | | | | |
| 1 | observational studies | not serious | not serious | not serious | not serious | publication bias strongly suspected^i^ | 147/391 (37.6%) | 348/1321 (26.3%) | **OR 1.68** (1.33 to 2.14) | **112 more per 1,000** (from 59 more to 170 more) | ⨁◯◯◯ Very low | IMPORTANT |
| **All-cause long-term mortality after redefining exposed cohort by defining FI according to the number of GI symptoms ≥3 (limit the total number of candidate symptoms to 4) (follow-up: range 90 days to 150+ days; assessed with: follow-up records)** | | | | | | | | | | | | |
| 1 | observational studies | not serious | not serious | not serious | not serious | publication bias strongly suspected^i^ | 37/79 (46.8%) | 458/1633 (28.0%) | **OR 2.26** (1.43 to 3.56) | **188 more per 1,000** (from 77 more to 301 more) | ⨁◯◯◯ Very low | IMPORTANT |
| **All-cause long-term mortality after redefining exposed cohort by defining FI according to the number of GI symptoms ≥1 (limit the total number of candidate symptoms to 5) (follow-up: range 90 days to 150+ days; assessed with: follow-up records)** | | | | | | | | | | | | |
| 3 | observational studies | not serious | not serious | not serious | not serious | publication bias strongly suspected^i^ | 477/1248 (38.2%) | 207/898 (23.1%) | **OR 2.65** (0.86 to 8.11) | **212 more per 1,000** (from 26 fewer to 478 more) | ⨁◯◯◯ Very low | IMPORTANT |
| **All-cause long-term mortality after redefining exposed cohort by defining FI according to the number of GI symptoms ≥2 (limit the total number of candidate symptoms to 5) (follow-up: range 90 days to 150+ days; assessed with: follow-up records)** | | | | | | | | | | | | |
| 1 | observational studies | not serious | not serious | not serious | not serious | publication bias strongly suspected^i^ | 197/520 (37.9%) | 298/1192 (25.0%) | **OR 1.83** (1.47 to 2.28) | **129 more per 1,000** (from 79 more to 182 more) | ⨁◯◯◯ Very low | IMPORTANT |
| **All-cause long-term mortality after redefining exposed cohort by defining FI according to the number of GI symptoms ≥3 (limit the total number of candidate symptoms to 5) (follow-up: range 90 days to 150+ days; assessed with: follow-up records)** | | | | | | | | | | | | |
| 1 | observational studies | not serious | not serious | not serious | not serious | publication bias strongly suspected^i^ | 74/146 (50.7%) | 421/1566 (26.9%) | **OR 2.80** (1.98 to 3.94) | **238 more per 1,000** (from 152 more to 323 more) | ⨁◯◯◯ Very low | IMPORTANT |
| **All-cause long-term mortality after redefining exposed cohort by defining FI according to symptom of vomiting (follow-up: range 90 days to 150+ days; assessed with: follow-up records)** | | | | | | | | | | | | |
| 1 | observational studies | not serious | not serious | not serious | not serious | publication bias strongly suspected^i^ | 120/432 (27.8%) | 375/1280 (29.3%) | **OR 0.93** (0.73 to 1.18) | **15 fewer per 1,000** (from 61 fewer to 35 more) | ⨁◯◯◯ Very low | IMPORTANT |
| **All-cause long-term mortality after redefining exposed cohort by defining FI according to symptom of absent bowel sounds (follow-up: range 90 days to 150+ days; assessed with: follow-up records)** | | | | | | | | | | | | |
| 1 | observational studies | not serious | not serious | not serious | not serious | publication bias strongly suspected^i^ | 137/370 (37.0%) | 358/1342 (26.7%) | **OR 1.62** (1.27 to 2.06) | **104 more per 1,000** (from 49 more to 162 more) | ⨁◯◯◯ Very low | IMPORTANT |
| **All-cause long-term mortality after redefining exposed cohort by defining FI according to symptom of abdominal distention (follow-up: range 90 days to 150+ days; assessed with: follow-up records)** | | | | | | | | | | | | |
| 1 | observational studies | not serious | not serious | not serious | not serious | publication bias strongly suspected^i^ | 37/67 (55.2%) | 458/1645 (27.8%) | **OR 3.20** (1.95 to 5.24) | **274 more per 1,000** (from 151 more to 391 more) | ⨁◯◯◯ Very low | IMPORTANT |
| **All-cause long-term mortality after redefining exposed cohort by defining FI according to symptom of diarrhea (follow-up: range 90 days to 150+ days; assessed with: follow-up records)** | | | | | | | | | | | | |
| 1 | observational studies | not serious | not serious | not serious | not serious | publication bias strongly suspected^i^ | 31/56 (55.4%) | 464/1656 (28.0%) | **OR 3.19** (1.86 to 5.45) | **274 more per 1,000** (from 140 more to 399 more) | ⨁◯◯◯ Very low | IMPORTANT |
| **All-cause long-term mortality after redefining exposed cohort by defining FI according to large GRV (as one of the symptoms) of 250±50 ml (follow-up: range 90 days to 150+ days; assessed with: follow-up records)** | | | | | | | | | | | | |
| 2 | observational studies | not serious | not serious | not serious | extremely serious^n^ | publication bias strongly suspected^i^ | 313/828 (37.8%) | 278/1101 (25.2%) | **OR 2.25** (0.01 to 473.08) | **179 more per 1,000** (from 249 fewer to 741 more) | ⨁◯◯◯ Very low | IMPORTANT |
| **All-cause long-term mortality after redefining exposed cohort by defining FI according to large GRV (as one of the symptoms) of 1000 ml (follow-up: range 90 days to 150+ days; assessed with: follow-up records)** | | | | | | | | | | | | |
| 1 | observational studies | not serious | not serious | not serious | not serious | publication bias strongly suspected^i^ | 55/128 (43.0%) | 440/1584 (27.8%) | **OR 1.96** (1.36 to 2.83) | **152 more per 1,000** (from 66 more to 243 more) | ⨁◯◯◯ Very low | IMPORTANT |
| **All-cause long-term mortality after redefining exposed cohort by defining FI according to large GRV (as one of the symptoms) of 500±50 ml (follow-up: range 90 days to 150+ days; assessed with: follow-up records)** | | | | | | | | | | | | |
| 2 | observational studies | not serious | not serious | not serious | serious^m^ | publication bias strongly suspected^i^ | 467/1353 (34.5%) | 523/2071 (25.3%) | **OR 1.67** (1.02 to 2.72) | **108 more per 1,000** (from 4 more to 226 more) | ⨁◯◯◯ Very low | IMPORTANT |
| **All-cause long-term mortality after redefining exposed cohort by defining FI according to large GRV (as the only symptom) of 250±50 ml (follow-up: range 90 days to 150+ days; assessed with: follow-up records)** | | | | | | | | | | | | |
| 1 | observational studies | not serious | not serious | not serious | not serious | publication bias strongly suspected^i^ | 237/690 (34.3%) | 258/1022 (25.2%) | **OR 1.55** (1.25 to 1.91) | **91 more per 1,000** (from 44 more to 140 more) | ⨁◯◯◯ Very low | IMPORTANT |
| **All-cause long-term mortality after redefining exposed cohort by defining FI according to large GRV (as the only symptom) of 1000 ml (follow-up: range 90 days to 150+ days; assessed with: follow-up records)** | | | | | | | | | | | | |
| 1 | observational studies | not serious | not serious | not serious | not serious | publication bias strongly suspected^i^ | 55/128 (43.0%) | 440/1584 (27.8%) | **OR 1.96** (1.36 to 2.83) | **152 more per 1,000** (from 66 more to 243 more) | ⨁◯◯◯ Very low | IMPORTANT |
| **All-cause long-term mortality after redefining exposed cohort by defining FI according to large GRV (as the only symptom) of 500±50 ml (follow-up: range 90 days to 150+ days; assessed with: follow-up records)** | | | | | | | | | | | | |
| 1 | observational studies | not serious | not serious | not serious | not serious | publication bias strongly suspected^i^ | 140/381 (36.7%) | 355/1331 (26.7%) | **OR 1.60** (1.25 to 2.03) | **101 more per 1,000** (from 46 more to 158 more) | ⨁◯◯◯ Very low | IMPORTANT |
| **All-cause long-term mortality after redefining exposed cohort by defining FI according to the measured interval of GRV (as one of the symptoms) of 24 hours (follow-up: range 90 days to 150+ days; assessed with: follow-up records)** | | | | | | | | | | | | |
| 2 | observational studies | not serious | not serious | not serious | not serious | publication bias strongly suspected^i^ | 564/1662 (33.9%) | 426/1762 (24.2%) | **OR 1.63** (0.82 to 3.24) | **100 more per 1,000** (from 34 fewer to 266 more) | ⨁◯◯◯ Very low | IMPORTANT |
| **All-cause long-term mortality after redefining exposed cohort by defining FI according to the measured interval of GRV (as one of the symptoms) of 4 hours (follow-up: range 90 days to 150+ days; assessed with: follow-up records)** | | | | | | | | | | | | |
| 1 | observational studies | serious^k^ | not serious | not serious | not serious | publication bias strongly suspected^i^ | 76/138 (55.1%) | 20/79 (25.3%) | **OR 3.62** (1.97 to 6.64) | **298 more per 1,000** (from 147 more to 439 more) | ⨁◯◯◯ Very low | IMPORTANT |
| **All-cause long-term mortality after redefining exposed cohort by defining FI according to the measured interval of GRV (as the only symptom) of 24 hours (follow-up: range 90 days to 150+ days; assessed with: follow-up records)** | | | | | | | | | | | | |
| 3 | observational studies | not serious | not serious | not serious | not serious | publication bias strongly suspected^i^ | 432/1199 (36.0%) | 1053/3937 (26.7%) | **OR 1.63** (1.27 to 2.09) | **106 more per 1,000** (from 49 more to 165 more) | ⨁◯◯◯ Very low | IMPORTANT |
| **All-cause long-term mortality after redefining exposed cohort by defining FI according to EF percentage of 50% (follow-up: range 90 days to 150+ days; assessed with: follow-up records)** | | | | | | | | | | | | |
| 1 | observational studies | not serious | not serious | not serious | not serious | publication bias strongly suspected^i^ | 456/1459 (31.3%) | 39/253 (15.4%) | **OR 2.49** (1.74 to 3.57) | **158 more per 1,000** (from 87 more to 240 more) | ⨁◯◯◯ Very low | IMPORTANT |
| **All-cause long-term mortality after redefining exposed cohort by defining FI according to EF percentage of 20% (follow-up: range 90 days to 150+ days; assessed with: follow-up records)** | | | | | | | | | | | | |
| 1 | observational studies | not serious | not serious | not serious | not serious | publication bias strongly suspected^i^ | 372/989 (37.6%) | 123/723 (17.0%) | **OR 2.94** (2.33 to 3.71) | **206 more per 1,000** (from 153 more to 262 more) | ⨁◯◯◯ Very low | IMPORTANT |
| **All-cause long-term mortality after redefining exposed cohort by defining FI according to EF percentage of 80% (follow-up: range 90 days to 150+ days; assessed with: follow-up records)** | | | | | | | | | | | | |
| 2 | observational studies | not serious | not serious | not serious | extremely serious^n^ | publication bias strongly suspected^i^ | 350/1035 (33.8%) | 51/224 (22.8%) | **OR 2.34** (0.01 to 383.22) | **181 more per 1,000** (from 225 fewer to 764 more) | ⨁◯◯◯ Very low | IMPORTANT |
| **All-cause long-term mortality in the study population with surgical patients accounted for 0% (follow-up: range 90 days to 150+ days; assessed with: follow-up records)** | | | | | | | | | | | | |
| 1 | observational studies | serious^k^ | not serious | not serious | not serious | publication bias strongly suspected^i^ | 76/138 (55.1%) | 20/79 (25.3%) | **OR 3.62** (1.97 to 6.64) | **298 more per 1,000** (from 147 more to 439 more) | ⨁◯◯◯ Very low | IMPORTANT |
| **All-cause long-term mortality in the study population with male patients accounted for ≤65% (follow-up: range 90 days to 150+ days; assessed with: medical records)** | | | | | | | | | | | | |
| 3 | observational studies | serious^k^ | not serious | not serious | not serious | publication bias strongly suspected^i^ | 838/2559 (32.7%) | 457/1907 (24.0%) | **OR 1.63** (1.40 to 1.90) | **100 more per 1,000** (from 67 more to 135 more) | ⨁◯◯◯ Very low | IMPORTANT |
| **All-cause long-term mortality in the study population with male patients accounted for >65% (follow-up: range 90 days to 150+ days; assessed with: follow-up records)** | | | | | | | | | | | | |
| 1 | observational studies | serious^k^ | not serious | not serious | not serious | publication bias strongly suspected^i^ | 76/138 (55.1%) | 20/79 (25.3%) | **OR 3.62** (1.97 to 6.64) | **298 more per 1,000** (from 147 more to 439 more) | ⨁◯◯◯ Very low | IMPORTANT |
| **All-cause long-term mortality in the study population with an average age of >55 (follow-up: range 90 days to 150+ days; assessed with: follow-up records)** | | | | | | | | | | | | |
| 4 | observational studies | not serious | not serious | not serious | not serious | publication bias strongly suspected^i^ | 914/2697 (33.9%) | 477/1986 (24.0%) | **OR 1.71** (1.20 to 2.43) | **111 more per 1,000** (from 35 more to 194 more) | ⨁◯◯◯ Very low | IMPORTANT |
| **All-cause long-term mortality in the study population with mechanical ventilation patients accounted for 75-95% (follow-up: range 90 days to 150+ days; assessed with: follow-up records)** | | | | | | | | | | | | |
| 18 | observational studies | not serious | not serious | not serious | not serious | none | 3214/9003 (35.7%) | 5696/21813 (26.1%) | **OR 1.92** (1.64 to 2.24) | **143 more per 1,000** (from 106 more to 181 more) | ⨁⨁◯◯ Low | IMPORTANT |
| **All-cause long-term mortality in the study population with average SOFA score of ≥8 (follow-up: range 90 days to 150+ days; assessed with: follow-up records)** | | | | | | | | | | | | |
| 18 | observational studies | not serious | not serious | not serious | not serious | none | 3214/9003 (35.7%) | 5696/21813 (26.1%) | **OR 1.92** (1.64 to 2.24) | **143 more per 1,000** (from 106 more to 181 more) | ⨁⨁◯◯ Low | IMPORTANT |
| **All-cause long-term mortality in the study population with average SOFA score of <8 (follow-up: range 90 days to 150+ days; assessed with: follow-up records)** | | | | | | | | | | | | |
| 2 | observational studies | serious^k^ | not serious | not serious | not serious | publication bias strongly suspected^i^ | 150/276 (54.3%) | 39/158 (24.7%) | **OR 3.63** (3.42 to 3.86) | **296 more per 1,000** (from 282 more to 312 more) | ⨁◯◯◯ Very low | IMPORTANT |
| **All-cause long-term mortality in the study population with average APACHE II score of <20 (follow-up: range 90 days to 150+ days; assessed with: follow-up records)** | | | | | | | | | | | | |
| 18 | observational studies | not serious | not serious | not serious | not serious | none | 3214/9003 (35.7%) | 5696/21813 (26.1%) | **OR 1.92** (1.64 to 2.24) | **143 more per 1,000** (from 106 more to 181 more) | ⨁⨁◯◯ Low | IMPORTANT |
| **All-cause mortality after redefining exposed cohort by defining FI according to GI symptoms cluster (follow-up: range 13 days to 150+ days; assessed with: medical records)** | | | | | | | | | | | | |
| 22 | observational studies | not serious | not serious | not serious | not serious | none | 2529/8441 (30.0%) | 4543/20058 (22.6%) | **OR 1.63** (1.28 to 2.08) | **97 more per 1,000** (from 46 more to 152 more) | ⨁⨁◯◯ Low | IMPORTANT |
| **All-cause mortality after redefining exposed cohort by defining FI according to large-GRV-containing GI symptoms cluster (follow-up: range 13 days to 150+ days; assessed with: medical records)** | | | | | | | | | | | | |
| 12 | observational studies | not serious | not serious | not serious | not serious | none | 1989/6623 (30.0%) | 3526/15478 (22.8%) | **OR 1.71** (1.32 to 2.21) | **108 more per 1,000** (from 52 more to 167 more) | ⨁⨁◯◯ Low | IMPORTANT |
| **All-cause mortality after redefining exposed cohort by defining FI according to only large GRV (follow-up: range 13 days to 150+ days; assessed with: medical records)** | | | | | | | | | | | | |
| 6 | observational studies | not serious | serious^l^ | not serious | not serious | publication bias strongly suspected^i^ | 275/872 (31.5%) | 495/2355 (21.0%) | **OR 1.45** (0.59 to 3.59) | **68 more per 1,000** (from 74 fewer to 278 more) | ⨁◯◯◯ Very low | IMPORTANT |
| **All-cause mortality after redefining exposed cohort by defining FI according to EF insufficiency (follow-up: range 13 days to 150+ days; assessed with: medical records)** | | | | | | | | | | | | |
| 6 | observational studies | not serious | not serious | not serious | not serious | publication bias strongly suspected^i^ | 489/1407 (34.8%) | 256/988 (25.9%) | **OR 1.85** (1.21 to 2.82) | **134 more per 1,000** (from 38 more to 237 more) | ⨁◯◯◯ Very low | IMPORTANT |
| **All-cause mortality after redefining exposed cohort by defining FI according to GI symptoms cluster without large GRV (follow-up: range 13 days to 150+ days; assessed with: medical records)** | | | | | | | | | | | | |
| 4 | observational studies | not serious | serious^l^ | not serious | not serious | publication bias strongly suspected^i^ | 265/946 (28.0%) | 522/2225 (23.5%) | **OR 1.41** (0.44 to 4.54) | **67 more per 1,000** (from 116 fewer to 347 more) | ⨁◯◯◯ Very low | IMPORTANT |
| **All-cause mortality after redefining exposed cohort by defining FI according to the number of GI symptoms ≥1 (no limit to the total number of candidate symptoms, which can range from 1-6) (follow-up: range 13 days to 150+ days; assessed with: medical records)** | | | | | | | | | | | | |
| 11 | observational studies | not serious | not serious | not serious | not serious | none | 1985/6564 (30.2%) | 3512/15390 (22.8%) | **OR 1.77** (1.42 to 2.20) | **115 more per 1,000** (from 68 more to 166 more) | ⨁⨁◯◯ Low | IMPORTANT |
| **All-cause mortality after redefining exposed cohort by defining FI according to the number of GI symptoms ≥2 (no limit to the total number of candidate symptoms, which can range from 1-6) (follow-up: range 13 days to 150+ days; assessed with: medical records)** | | | | | | | | | | | | |
| 3 | observational studies | not serious | not serious | not serious | serious^h^ | publication bias strongly suspected^i^ | 234/682 (34.3%) | 479/2275 (21.1%) | **OR 1.36** (0.15 to 12.59) | **56 more per 1,000** (from 172 fewer to 560 more) | ⨁◯◯◯ Very low | IMPORTANT |
| **All-cause mortality after redefining exposed cohort by defining FI according to the number of GI symptoms ≥3 (no limit to the total number of candidate symptoms, which can range from 1-6) (follow-up: range 13 days to 150+ days; assessed with: medical records)** | | | | | | | | | | | | |
| 2 | observational studies | not serious | not serious | not serious | not serious | publication bias strongly suspected^i^ | 77/153 (50.3%) | 618/2657 (23.3%) | **OR 2.82** (1.64 to 4.85) | **228 more per 1,000** (from 99 more to 363 more) | ⨁◯◯◯ Very low | IMPORTANT |
| **All-cause mortality after redefining exposed cohort by defining FI according to the number of GI symptoms ≥1 (limit the total number of candidate symptoms to 4) (follow-up: range 13 days to 150+ days; assessed with: medical records)** | | | | | | | | | | | | |
| 7 | observational studies | not serious | not serious | not serious | not serious | publication bias strongly suspected^i^ | 2029/6934 (29.3%) | 3751/16915 (22.2%) | **OR 1.76** (1.33 to 2.33) | **112 more per 1,000** (from 53 more to 177 more) | ⨁◯◯◯ Very low | IMPORTANT |
| **All-cause mortality after redefining exposed cohort by defining FI according to the number of GI symptoms ≥2 (limit the total number of candidate symptoms to 4) (follow-up: range 13 days to 150+ days; assessed with: medical records)** | | | | | | | | | | | | |
| 3 | observational studies | not serious | not serious | not serious | serious^m^ | publication bias strongly suspected^i^ | 266/871 (30.5%) | 650/3651 (17.8%) | **OR 2.21** (1.12 to 4.37) | **146 more per 1,000** (from 17 more to 308 more) | ⨁◯◯◯ Very low | IMPORTANT |
| **All-cause mortality after redefining exposed cohort by defining FI according to the number of GI symptoms ≥3 (limit the total number of candidate symptoms to 4) (follow-up: range 13 days to 150+ days; assessed with: medical records)** | | | | | | | | | | | | |
| 3 | observational studies | not serious | not serious | not serious | not serious | publication bias strongly suspected^i^ | 63/164 (38.4%) | 853/4358 (19.6%) | **OR 2.62** (1.57 to 4.39) | **194 more per 1,000** (from 81 more to 321 more) | ⨁◯◯◯ Very low | IMPORTANT |
| **All-cause mortality after redefining exposed cohort by defining FI according to the number of GI symptoms ≥1 (limit the total number of candidate symptoms to 5) (follow-up: range 13 days to 150+ days; assessed with: medical records)** | | | | | | | | | | | | |
| 15 | observational studies | not serious | not serious | not serious | not serious | none | 1436/4820 (29.8%) | 1339/6271 (21.4%) | **OR 1.96** (1.60 to 2.39) | **134 more per 1,000** (from 89 more to 180 more) | ⨁⨁◯◯ Low | IMPORTANT |
| **All-cause mortality after redefining exposed cohort by defining FI according to the number of GI symptoms ≥2 (limit the total number of candidate symptoms to 5) (follow-up: range 13 days to 150+ days; assessed with: medical records)** | | | | | | | | | | | | |
| 4 | observational studies | not serious | not serious | not serious | not serious | publication bias strongly suspected^i^ | 346/1202 (28.8%) | 588/3467 (17.0%) | **OR 1.75** (0.53 to 5.71) | **94 more per 1,000** (from 72 fewer to 369 more) | ⨁◯◯◯ Very low | IMPORTANT |
| **All-cause mortality after redefining exposed cohort by defining FI according to the number of GI symptoms ≥3 (limit the total number of candidate symptoms to 5) (follow-up: range 13 days to 150+ days; assessed with: medical records)** | | | | | | | | | | | | |
| 3 | observational studies | not serious | not serious | not serious | not serious | publication bias strongly suspected^i^ | 129/299 (43.1%) | 787/4223 (18.6%) | **OR 3.54** (1.72 to 7.29) | **261 more per 1,000** (from 96 more to 439 more) | ⨁◯◯◯ Very low | IMPORTANT |
| **All-cause mortality after redefining exposed cohort by defining FI according to symptom of vomiting (follow-up: range 13 days to 150+ days; assessed with: medical records)** | | | | | | | | | | | | |
| 3 | observational studies | not serious | not serious | not serious | not serious | publication bias strongly suspected^i^ | 201/926 (21.7%) | 715/3596 (19.9%) | **OR 1.24** (0.47 to 3.26) | **36 more per 1,000** (from 94 fewer to 248 more) | ⨁◯◯◯ Very low | IMPORTANT |
| **All-cause mortality after redefining exposed cohort by defining FI according to symptom of absent bowel sounds (follow-up: range 13 days to 150+ days; assessed with: medical records)** | | | | | | | | | | | | |
| 3 | observational studies | not serious | not serious | not serious | not serious | publication bias strongly suspected^i^ | 224/778 (28.8%) | 692/3744 (18.5%) | **OR 1.69** (0.55 to 5.22) | **92 more per 1,000** (from 74 fewer to 357 more) | ⨁◯◯◯ Very low | IMPORTANT |
| **All-cause mortality after redefining exposed cohort by defining FI according to symptom of abdominal distention (follow-up: range 13 days to 150+ days; assessed with: medical records)** | | | | | | | | | | | | |
| 3 | observational studies | not serious | not serious | not serious | serious^m^ | publication bias strongly suspected^i^ | 144/451 (31.9%) | 772/4071 (19.0%) | **OR 2.92** (1.04 to 8.17) | **216 more per 1,000** (from 6 more to 467 more) | ⨁◯◯◯ Very low | IMPORTANT |
| **All-cause mortality after redefining exposed cohort by defining FI according to symptom of diarrhea (follow-up: range 13 days to 150+ days; assessed with: medical records)** | | | | | | | | | | | | |
| 3 | observational studies | not serious | not serious | not serious | not serious | publication bias strongly suspected^i^ | 82/215 (38.1%) | 834/4307 (19.4%) | **OR 2.82** (1.94 to 4.10) | **210 more per 1,000** (from 124 more to 302 more) | ⨁◯◯◯ Very low | IMPORTANT |
| **All-cause mortality after redefining exposed cohort by defining FI according to large GRV (as one of the symptoms) of 500±50 ml (follow-up: range 13 days to 150+ days; assessed with: medical records)** | | | | | | | | | | | | |
| 7 | observational studies | serious^k^ | not serious | not serious | serious^m^ | publication bias strongly suspected^i^ | 223/669 (33.3%) | 628/2802 (22.4%) | **OR 1.71** (1.15 to 2.54) | **107 more per 1,000** (from 25 more to 199 more) | ⨁◯◯◯ Very low | IMPORTANT |
| **All-cause mortality after redefining exposed cohort by defining FI according to large GRV (as one of the symptoms) of 75±50 ml (follow-up: range 13 days to 150+ days; assessed with: medical records)** | | | | | | | | | | | | |
| 1 | observational studies | not serious | not serious | not serious | not serious | publication bias strongly suspected^i^ | 6/29 (20.7%) | 12/32 (37.5%) | **OR 0.43** (0.14 to 1.37) | **170 fewer per 1,000** (from 298 fewer to 76 more) | ⨁◯◯◯ Very low | IMPORTANT |
| **All-cause mortality after redefining exposed cohort by defining FI according to large GRV (as one of the symptoms) of 250±50 ml (follow-up: range 13 days to 150+ days; assessed with: medical records)** | | | | | | | | | | | | |
| 13 | observational studies | not serious | not serious | not serious | not serious | none | 909/2847 (31.9%) | 1362/5784 (23.5%) | **OR 1.89** (1.51 to 2.36) | **132 more per 1,000** (from 82 more to 185 more) | ⨁⨁◯◯ Low | IMPORTANT |
| **All-cause mortality after redefining exposed cohort by defining FI according to large GRV (as one of the symptoms) of 1000 ml (follow-up: range 13 days to 150+ days; assessed with: medical records)** | | | | | | | | | | | | |
| 1 | observational studies | not serious | not serious | not serious | not serious | publication bias strongly suspected^i^ | 55/128 (43.0%) | 440/1584 (27.8%) | **OR 1.96** (1.36 to 2.83) | **152 more per 1,000** (from 66 more to 243 more) | ⨁◯◯◯ Very low | IMPORTANT |
| **All-cause mortality after redefining exposed cohort by defining FI according to large GRV (as the only symptom) of 75±50 ml (follow-up: range 13 days to 150+ days)** | | | | | | | | | | | | |
| 1 | observational studies | serious^k^ | not serious | not serious | not serious | publication bias strongly suspected^i^ | 6/29 (20.7%) | 12/32 (37.5%) | **OR 0.43** (0.14 to 1.37) | **170 fewer per 1,000** (from 298 fewer to 76 more) | ⨁◯◯◯ Very low | IMPORTANT |
| **All-cause mortality after redefining exposed cohort by defining FI according to large GRV (as the only symptom) of 250±50 ml (follow-up: range 13 days to 150+ days; assessed with: medical records)** | | | | | | | | | | | | |
| 3 | observational studies | not serious | not serious | not serious | not serious | publication bias strongly suspected^i^ | 261/773 (33.8%) | 273/1148 (23.8%) | **OR 2.23** (0.57 to 8.75) | **172 more per 1,000** (from 87 fewer to 494 more) | ⨁◯◯◯ Very low | IMPORTANT |
| **All-cause mortality after redefining exposed cohort by defining FI according to large GRV (as the only symptom) of 1000 ml (follow-up: range 13 days to 150+ days; assessed with: medical records)** | | | | | | | | | | | | |
| 1 | observational studies | not serious | not serious | not serious | not serious | publication bias strongly suspected^i^ | 55/128 (43.0%) | 440/1584 (27.8%) | **OR 1.96** (1.36 to 2.83) | **152 more per 1,000** (from 66 more to 243 more) | ⨁◯◯◯ Very low | IMPORTANT |
| **All-cause mortality after redefining exposed cohort by defining FI according to large GRV (as the only symptom) of 500±50 ml (follow-up: range 13 days to 150+ days; assessed with: medical records)** | | | | | | | | | | | | |
| 3 | observational studies | not serious | not serious | not serious | not serious | publication bias strongly suspected^i^ | 148/451 (32.8%) | 565/2506 (22.5%) | **OR 1.37** (0.49 to 3.84) | **60 more per 1,000** (from 101 fewer to 302 more) | ⨁◯◯◯ Very low | IMPORTANT |
| **All-cause mortality after redefining exposed cohort by defining FI according to the measured interval of GRV (as one of the symptoms) of 4 hours (follow-up: range 13 days to 150+ days; assessed with: medical records)** | | | | | | | | | | | | |
| 7 | observational studies | not serious | not serious | not serious | not serious | publication bias strongly suspected^i^ | 271/659 (41.1%) | 269/986 (27.3%) | **OR 1.71** (0.92 to 3.20) | **118 more per 1,000** (from 16 fewer to 273 more) | ⨁◯◯◯ Very low | IMPORTANT |
| **All-cause mortality after redefining exposed cohort by defining FI according to the measured interval of GRV (as one of the symptoms) of 6 hours (follow-up: range 13 days to 150+ days; assessed with: medical records)** | | | | | | | | | | | | |
| 2 | observational studies | not serious | not serious | not serious | serious^m^ | publication bias strongly suspected^i^ | 41/183 (22.4%) | 43/344 (12.5%) | **OR 2.18** (1.16 to 4.11) | **112 more per 1,000** (from 17 more to 245 more) | ⨁◯◯◯ Very low | IMPORTANT |
| **All-cause mortality after redefining exposed cohort by defining FI according to the measured interval of GRV (as one of the symptoms) of 24 hours (follow-up: range 13 days to 150+ days; assessed with: medical records)** | | | | | | | | | | | | |
| 3 | observational studies | not serious | not serious | not serious | not serious | publication bias strongly suspected^i^ | 244/779 (31.3%) | 463/2226 (20.8%) | **OR 1.54** (1.34 to 1.78) | **80 more per 1,000** (from 52 more to 111 more) | ⨁◯◯◯ Very low | IMPORTANT |
| **All-cause mortality after redefining exposed cohort by defining FI according to the measured interval of GRV (as the only symptom) of 4 hours (follow-up: range 13 days to 150+ days; assessed with: medical records)** | | | | | | | | | | | | |
| 3 | observational studies | not serious | serious^l^ | not serious | serious^h^ | publication bias strongly suspected^i^ | 32/149 (21.5%) | 37/209 (17.7%) | **OR 1.07** (0.08 to 15.20) | **10 more per 1,000** (from 160 fewer to 589 more) | ⨁◯◯◯ Very low | IMPORTANT |
| **All-cause mortality after redefining exposed cohort by defining FI according to the measured interval of GRV (as the only symptom) of 24 hours (follow-up: range 13 days to 150+ days; assessed with: medical records)** | | | | | | | | | | | | |
| 2 | observational studies | not serious | not serious | not serious | serious^m^ | publication bias strongly suspected^i^ | 238/693 (34.3%) | 457/2117 (21.6%) | **OR 1.55** (1.03 to 2.35) | **83 more per 1,000** (from 5 more to 177 more) | ⨁◯◯◯ Very low | IMPORTANT |
| **All-cause mortality after redefining exposed cohort by defining FI according to EF percentage of 80% (follow-up: range 13 days to 150+ days; assessed with: medical records)** | | | | | | | | | | | | |
| 6 | observational studies | not serious | not serious | not serious | not serious | publication bias strongly suspected^i^ | 489/1407 (34.8%) | 256/988 (25.9%) | **OR 1.85** (1.21 to 2.82) | **134 more per 1,000** (from 38 more to 237 more) | ⨁◯◯◯ Very low | IMPORTANT |
| **All-cause mortality after redefining exposed cohort by defining FI according to EF percentage of 50% (follow-up: range 13 days to 150+ days; assessed with: medical records)** | | | | | | | | | | | | |
| 1 | observational studies | not serious | not serious | not serious | not serious | publication bias strongly suspected^i^ | 456/1459 (31.3%) | 39/253 (15.4%) | **OR 2.49** (1.74 to 3.57) | **158 more per 1,000** (from 87 more to 240 more) | ⨁◯◯◯ Very low | IMPORTANT |
| **All-cause mortality after redefining exposed cohort by defining FI according to EF percentage of 20% (follow-up: range 13 days to 150+ days; assessed with: medical records)** | | | | | | | | | | | | |
| 1 | observational studies | not serious | not serious | not serious | not serious | publication bias strongly suspected^i^ | 372/989 (37.6%) | 123/723 (17.0%) | **OR 2.94** (2.33 to 3.71) | **206 more per 1,000** (from 153 more to 262 more) | ⨁◯◯◯ Very low | IMPORTANT |
| **All-cause mortality in the study population with surgical patients accounted for >25% (follow-up: range 13 days to 150+ days; assessed with: medical records)** | | | | | | | | | | | | |
| 5 | observational studies | not serious | not serious | not serious | not serious | publication bias strongly suspected^i^ | 1335/4441 (30.1%) | 2876/12394 (23.2%) | **OR 1.61** (1.26 to 2.06) | **95 more per 1,000** (from 44 more to 152 more) | ⨁◯◯◯ Very low | IMPORTANT |
| **All-cause mortality in the study population with surgical patients accounted for 0% (follow-up: range 13 days to 150+ days; assessed with: medical records)** | | | | | | | | | | | | |
| 11 | observational studies | not serious | serious^l^ | not serious | not serious | none | 201/743 (27.1%) | 135/873 (15.5%) | **OR 1.34** (0.70 to 2.58) | **42 more per 1,000** (from 41 fewer to 166 more) | ⨁◯◯◯ Very low | IMPORTANT |
| **All-cause mortality in the study population with surgical patients accounted for 15-20% (follow-up: range 13 days to 150+ days; assessed with: medical records)** | | | | | | | | | | | | |
| 2 | observational studies | not serious | not serious | not serious | not serious | publication bias strongly suspected^i^ | 256/772 (33.2%) | 430/1615 (26.6%) | **OR 1.40** (0.21 to 9.43) | **71 more per 1,000** (from 195 fewer to 508 more) | ⨁◯◯◯ Very low | IMPORTANT |
| **All-cause mortality in the study population with male patients accounted for ≤65% (follow-up: range 13 days to 150+ days; assessed with: medical records)** | | | | | | | | | | | | |
| 12 | observational studies | not serious | not serious | not serious | not serious | none | 2445/7851 (31.1%) | 3722/15897 (23.4%) | **OR 1.65** (1.36 to 2.00) | **101 more per 1,000** (from 60 more to 145 more) | ⨁⨁◯◯ Low | IMPORTANT |
| **All-cause mortality in the study population with male patients accounted for >65% (follow-up: range 13 days to 150+ days; assessed with: medical records)** | | | | | | | | | | | | |
| 14 | observational studies | not serious | serious^l^ | not serious | not serious | none | 366/1248 (29.3%) | 583/3088 (18.9%) | **OR 1.56** (0.99 to 2.45) | **78 more per 1,000** (from 2 fewer to 174 more) | ⨁◯◯◯ Very low | IMPORTANT |
| **All-cause mortality in the study population with an average age of >55 (follow-up: range 13 days to 150+ days; assessed with: medical records)** | | | | | | | | | | | | |
| 19 | observational studies | not serious | not serious | not serious | not serious | none | 2774/8697 (31.9%) | 4247/18543 (22.9%) | **OR 1.79** (1.54 to 2.09) | **118 more per 1,000** (from 85 more to 154 more) | ⨁⨁◯◯ Low | IMPORTANT |
| **All-cause mortality in the study population with an average age of ≤55 (follow-up: range 13 days to 150+ days; assessed with: medical records)** | | | | | | | | | | | | |
| 7 | observational studies | serious^k^ | serious^l^ | not serious | not serious | publication bias strongly suspected^i^ | 37/402 (9.2%) | 58/442 (13.1%) | **OR 0.92** (0.30 to 2.89) | **9 fewer per 1,000** (from 88 fewer to 173 more) | ⨁◯◯◯ Very low | IMPORTANT |
| **All-cause mortality in the study population with trauma patients accounted for <25% (follow-up: range 13 days to 150+ days; assessed with: medical records)** | | | | | | | | | | | | |
| 5 | observational studies | not serious | not serious | not serious | not serious | publication bias strongly suspected^i^ | 264/786 (33.6%) | 419/1596 (26.3%) | **OR 1.60** (0.81 to 3.16) | **100 more per 1,000** (from 39 fewer to 267 more) | ⨁◯◯◯ Very low | IMPORTANT |
| **All-cause mortality in the study population with trauma patients accounted for 40-60% (follow-up: range 13 days to 150+ days; assessed with: medical records)** | | | | | | | | | | | | |
| 1 | observational studies | serious^k^ | not serious | not serious | not serious | publication bias strongly suspected^i^ | 9/21 (42.9%) | 14/51 (27.5%) | **OR 1.98** (0.69 to 5.73) | **154 more per 1,000** (from 67 fewer to 410 more) | ⨁◯◯◯ Very low | IMPORTANT |
| **All-cause mortality in the study population with trauma patients accounted for 100% (follow-up: range 13 days to 150+ days; assessed with: medical records)** | | | | | | | | | | | | |
| 3 | observational studies | serious^k^ | not serious | not serious | not serious | publication bias strongly suspected^i^ | 15/175 (8.6%) | 38/266 (14.3%) | **OR 0.56** (0.25 to 1.27) | **57 fewer per 1,000** (from 103 fewer to 32 more) | ⨁◯◯◯ Very low | IMPORTANT |
| **All-cause mortality in the study population with burn patients accounted for 100% (follow-up: range 13 days to 150+ days; assessed with: medical records)** | | | | | | | | | | | | |
| 3 | observational studies | serious^k^ | not serious | not serious | not serious | publication bias strongly suspected^i^ | 15/76 (19.7%) | 8/88 (9.1%) | **OR 5.51** (4.86 to 6.24) | **264 more per 1,000** (from 236 more to 293 more) | ⨁◯◯◯ Very low | IMPORTANT |
| **All-cause mortality in the study population with abdominal surgery patients accounted for 15% (follow-up: range 13 days to 150+ days; assessed with: medical records)** | | | | | | | | | | | | |
| 4 | observational studies | not serious | not serious | not serious | not serious | publication bias strongly suspected^i^ | 107/323 (33.1%) | 98/510 (19.2%) | **OR 1.99** (1.55 to 2.54) | **129 more per 1,000** (from 77 more to 184 more) | ⨁◯◯◯ Very low | IMPORTANT |
| **All-cause mortality in the study population with abdominal surgery patients accounted for 5% (follow-up: range 13 days to 150+ days; assessed with: medical records)** | | | | | | | | | | | | |
| 1 | observational studies | not serious | not serious | not serious | serious^m^ | publication bias strongly suspected^i^ | 177/576 (30.7%) | 344/1312 (26.2%) | **OR 1.25** (1.01 to 1.55) | **45 more per 1,000** (from 2 more to 93 more) | ⨁◯◯◯ Very low | IMPORTANT |
| **All-cause mortality in the study population with digestive diseases patients accounted for <10% (follow-up: range 13 days to 150+ days; assessed with: medical records)** | | | | | | | | | | | | |
| 5 | observational studies | not serious | not serious | not serious | serious^m^ | publication bias strongly suspected^i^ | 284/899 (31.6%) | 442/1822 (24.3%) | **OR 1.63** (1.15 to 2.30) | **100 more per 1,000** (from 27 more to 182 more) | ⨁◯◯◯ Very low | IMPORTANT |
| **All-cause mortality in the study population with sepsis patients accounted for <25% (follow-up: range 13 days to 150+ days; assessed with: medical records)** | | | | | | | | | | | | |
| 15 | observational studies | not serious | not serious | not serious | not serious | none | 657/2343 (28.0%) | 2181/12300 (17.7%) | **OR 2.04** (1.72 to 2.42) | **128 more per 1,000** (from 93 more to 165 more) | ⨁⨁◯◯ Low | IMPORTANT |
| **All-cause mortality in the study population with sepsis patients accounted for 100% (follow-up: range 13 days to 150+ days; assessed with: medical records)** | | | | | | | | | | | | |
| 5 | observational studies | serious^k^ | not serious | not serious | not serious | publication bias strongly suspected^i^ | 69/229 (30.1%) | 60/384 (15.6%) | **OR 2.77** (1.66 to 4.61) | **183 more per 1,000** (from 79 more to 304 more) | ⨁◯◯◯ Very low | IMPORTANT |
| **All-cause mortality in the study population with sepsis patients accounted for 25-55% (follow-up: range 13 days to 150+ days; assessed with: medical records)** | | | | | | | | | | | | |
| 3 | observational studies | not serious | not serious | not serious | not serious | publication bias strongly suspected^i^ | 151/409 (36.9%) | 169/627 (27.0%) | **OR 1.61** (0.76 to 3.38) | **103 more per 1,000** (from 51 fewer to 285 more) | ⨁◯◯◯ Very low | IMPORTANT |
| **All-cause mortality in the study population with mechanical ventilation patients accounted for 75-95% (follow-up: range 13 days to 150+ days; assessed with: medical records)** | | | | | | | | | | | | |
| 40^d^ | observational studies | not serious | not serious | not serious | not serious | none | 5026/18325 (27.4%) | 8153/44410 (18.4%) | **OR 2.25** (2.01 to 2.52) | **152 more per 1,000** (from 128 more to 178 more) | ⨁⨁◯◯ Low | IMPORTANT |
| **All-cause mortality in the study population with mechanical ventilation patients accounted for 100% (follow-up: range 13 days to 150+ days; assessed with: medical records)** | | | | | | | | | | | | |
| 7 | observational studies | not serious | not serious | not serious | not serious | publication bias strongly suspected^i^ | 1494/4817 (31.0%) | 3189/13497 (23.6%) | **OR 2.02** (1.20 to 3.43) | **148 more per 1,000** (from 34 more to 279 more) | ⨁◯◯◯ Very low | IMPORTANT |
| **All-cause mortality in the study population with mechanical ventilation patients accounted for <30% (follow-up: range 13 days to 150+ days; assessed with: medical records)** | | | | | | | | | | | | |
| 11 | observational studies | not serious | not serious | not serious | not serious | none | 420/1531 (27.4%) | 1780/10547 (16.9%) | **OR 2.18** (1.91 to 2.49) | **138 more per 1,000** (from 111 more to 167 more) | ⨁⨁◯◯ Low | IMPORTANT |
| **All-cause mortality in the study population with average SOFA score of <8 (follow-up: range 13 days to 150+ days; assessed with: medical records)** | | | | | | | | | | | | |
| 17 | observational studies | not serious | not serious | not serious | not serious | none | 720/2162 (33.3%) | 1881/11008 (17.1%) | **OR 2.36** (2.07 to 2.70) | **156 more per 1,000** (from 128 more to 187 more) | ⨁⨁◯◯ Low | IMPORTANT |
| **All-cause mortality in the study population with average SOFA score of ≥8 (follow-up: range 13 days to 150+ days; assessed with: medical records)** | | | | | | | | | | | | |
| 42^d^ | observational studies | not serious | not serious | not serious | not serious | none | 5110/18655 (27.4%) | 8164/44532 (18.3%) | **OR 2.24** (2.00 to 2.52) | **151 more per 1,000** (from 127 more to 178 more) | ⨁⨁◯◯ Low | IMPORTANT |
| **All-cause mortality in the study population with average APACHE II score of <20 (follow-up: range 13 days to 150+ days; assessed with: medical records)** | | | | | | | | | | | | |
| 57^d^ | observational studies | not serious | not serious | not serious | not serious | none | 5617/20290 (27.7%) | 10119/55660 (18.2%) | **OR 2.21** (2.01 to 2.43) | **148 more per 1,000** (from 127 more to 169 more) | ⨁⨁◯◯ Low | IMPORTANT |
| **All-cause mortality in the study population with average APACHE II score of ≥20 (follow-up: range 13 days to 150+ days; assessed with: medical records)** | | | | | | | | | | | | |
| 5 | observational studies | not serious | not serious | not serious | serious^m^ | none | 1489/4757 (31.3%) | 3188/13439 (23.7%) | **OR 1.95** (1.08 to 3.50) | **140 more per 1,000** (from 14 more to 284 more) | ⨁◯◯◯ Very low | IMPORTANT |
| **Incidence of pneumonia after redefining exposed cohort by defining FI according to GI symptoms cluster (follow-up: range 13 days to 90+ days; assessed with: medical records)** | | | | | | | | | | | | |
| 4 | observational studies | serious^k^ | not serious | not serious | not serious | publication bias strongly suspected^i^ | 55/315 (17.5%) | 48/502 (9.6%) | **OR 1.86** (1.23 to 2.83) | **69 more per 1,000** (from 19 more to 135 more) | ⨁◯◯◯ Very low | IMPORTANT |
| **Incidence of pneumonia after redefining exposed cohort by defining FI according to large-GRV-containing GI symptoms cluster (follow-up: range 13 days to 90+ days; assessed with: medical records)** | | | | | | | | | | | | |
| 2 | observational studies | serious^k^ | not serious | not serious | not serious | publication bias strongly suspected^i^ | 32/232 (13.8%) | 22/376 (5.9%) | **OR 2.30** (0.81 to 6.48) | **67 more per 1,000** (from 11 fewer to 229 more) | ⨁◯◯◯ Very low | IMPORTANT |
| **Incidence of pneumonia after redefining exposed cohort by defining FI according to only large GRV (follow-up: range 13 days to 90+ days; assessed with: medical records)** | | | | | | | | | | | | |
| 2 | observational studies | serious^k^ | not serious | not serious | not serious | publication bias strongly suspected^i^ | 23/83 (27.7%) | 26/126 (20.6%) | **OR 1.51** (0.49 to 4.58) | **76 more per 1,000** (from 93 fewer to 337 more) | ⨁◯◯◯ Very low | IMPORTANT |
| **Incidence of pneumonia after redefining exposed cohort by defining FI according to the number of GI symptoms ≥1 (no limit to the total number of candidate symptoms, which can range from 1-6) (follow-up: range 13 days to 90+ days; assessed with: medical records)** | | | | | | | | | | | | |
| 2 | observational studies | serious^k^ | not serious | not serious | not serious | publication bias strongly suspected^i^ | 32/232 (13.8%) | 22/376 (5.9%) | **OR 2.30** (0.81 to 6.48) | **67 more per 1,000** (from 11 fewer to 229 more) | ⨁◯◯◯ Very low | IMPORTANT |
| **Incidence of pneumonia after redefining exposed cohort by defining FI according to the number of GI symptoms ≥1 (limit the total number of candidate symptoms to 4) (follow-up: range 13 days to 90+ days; assessed with: medical records)** | | | | | | | | | | | | |
| 1 | observational studies | serious^k^ | not serious | not serious | serious^h^ | publication bias strongly suspected^i^ | 2/162 (1.2%) | 2/293 (0.7%) | **OR 1.82** (0.25 to 13.03) | **6 more per 1,000** (from 5 fewer to 75 more) | ⨁◯◯◯ Very low | IMPORTANT |
| **Incidence of pneumonia after redefining exposed cohort by defining FI according to large GRV (as one of the symptoms) of 500±50 ml (follow-up: range 13 days to 90+ days; assessed with: medical records)** | | | | | | | | | | | | |
| 1 | observational studies | serious^k^ | not serious | not serious | serious^m^ | publication bias strongly suspected^i^ | 30/70 (42.9%) | 20/83 (24.1%) | **OR 2.36** (1.18 to 4.71) | **187 more per 1,000** (from 32 more to 358 more) | ⨁◯◯◯ Very low | IMPORTANT |
| **Incidence of pneumonia after redefining exposed cohort by defining FI according to large GRV (as one of the symptoms) of 250±50 ml (follow-up: range 13 days to 90+ days; assessed with: medical records)** | | | | | | | | | | | | |
| 3 | observational studies | serious^k^ | not serious | not serious | serious^m^ | publication bias strongly suspected^i^ | 25/245 (10.2%) | 28/419 (6.7%) | **OR 1.54** (1.13 to 2.09) | **33 more per 1,000** (from 8 more to 63 more) | ⨁◯◯◯ Very low | IMPORTANT |
| **Incidence of pneumonia after redefining exposed cohort by defining FI according to large GRV (as the only symptom) of 250±50 ml (follow-up: range 13 days to 90+ days; assessed with: medical records)** | | | | | | | | | | | | |
| 2 | observational studies | serious^k^ | not serious | not serious | not serious | publication bias strongly suspected^i^ | 23/83 (27.7%) | 26/126 (20.6%) | **OR 1.51** (0.49 to 4.68) | **76 more per 1,000** (from 93 fewer to 343 more) | ⨁◯◯◯ Very low | IMPORTANT |
| **Incidence of pneumonia after redefining exposed cohort by defining FI according to the measured interval of GRV (as one of the symptoms) of 4 hours (follow-up: range 13 days to 90+ days; assessed with: medical records)** | | | | | | | | | | | | |
| 2 | observational studies | serious^k^ | not serious | not serious | serious^h^ | publication bias strongly suspected^i^ | 45/123 (36.6%) | 41/180 (22.8%) | **OR 1.89** (0.08 to 45.27) | **130 more per 1,000** (from 205 fewer to 703 more) | ⨁◯◯◯ Very low | IMPORTANT |
| **Incidence of pneumonia after redefining exposed cohort by defining FI according to the measured interval of GRV (as one of the symptoms) of 6 hours (follow-up: range 13 days to 90+ days; assessed with: medical records)** | | | | | | | | | | | | |
| 1 | observational studies | serious^k^ | not serious | not serious | serious^h^ | publication bias strongly suspected^i^ | 2/162 (1.2%) | 2/293 (0.7%) | **OR 1.82** (0.25 to 13.03) | **6 more per 1,000** (from 5 fewer to 75 more) | ⨁◯◯◯ Very low | IMPORTANT |
| **Incidence of pneumonia after redefining exposed cohort by defining FI according to the measured interval of GRV (as the only symptom) of 4 hours (follow-up: range 13 days to 90+ days; assessed with: medical records)** | | | | | | | | | | | | |
| 1 | observational studies | serious^k^ | not serious | not serious | not serious | publication bias strongly suspected^i^ | 15/53 (28.3%) | 21/97 (21.6%) | **OR 1.43** (0.66 to 3.08) | **67 more per 1,000** (from 62 fewer to 243 more) | ⨁◯◯◯ Very low | IMPORTANT |
| **Incidence of pneumonia in the study population with surgical patients accounted for >25% (follow-up: range 13 days to 90+ days; assessed with: medical records)** | | | | | | | | | | | | |
| 2 | observational studies | serious^k^ | not serious | not serious | not serious | publication bias strongly suspected^i^ | 32/232 (13.8%) | 22/376 (5.9%) | **OR 2.30** (0.81 to 6.48) | **67 more per 1,000** (from 11 fewer to 229 more) | ⨁◯◯◯ Very low | IMPORTANT |
| **Incidence of pneumonia in the study population with surgical patients accounted for 0% (follow-up: range 13 days to 90+ days; assessed with: medical records)** | | | | | | | | | | | | |
| 2 | observational studies | serious^k^ | not serious | not serious | not serious | publication bias strongly suspected^i^ | 23/83 (27.7%) | 26/126 (20.6%) | **OR 1.51** (0.49 to 4.68) | **76 more per 1,000** (from 93 fewer to 343 more) | ⨁◯◯◯ Very low | IMPORTANT |
| **Incidence of pneumonia in the study population with male patients accounted for ≤65% (follow-up: range 13 days to 90+ days; assessed with: medical records)** | | | | | | | | | | | | |
| 3 | observational studies | serious^k^ | not serious | not serious | not serious | publication bias strongly suspected^i^ | 47/285 (16.5%) | 43/473 (9.1%) | **OR 1.88** (0.90 to 3.93) | **67 more per 1,000** (from 8 fewer to 191 more) | ⨁◯◯◯ Very low | IMPORTANT |
| **Incidence of pneumonia in the study population with male patients accounted for >65% (follow-up: range 13 days to 90+ days; assessed with: medical records)** | | | | | | | | | | | | |
| 1 | observational studies | serious^k^ | not serious | not serious | not serious | publication bias strongly suspected^i^ | 8/30 (26.7%) | 5/29 (17.2%) | **OR 1.75** (0.50 to 6.14) | **95 more per 1,000** (from 78 fewer to 389 more) | ⨁◯◯◯ Very low | IMPORTANT |
| **Incidence of pneumonia in the study population with an average age of >55 (follow-up: range 13 days to 90+ days; assessed with: medical records)** | | | | | | | | | | | | |
| 3 | observational studies | serious^k^ | not serious | not serious | not serious | publication bias strongly suspected^i^ | 47/285 (16.5%) | 43/473 (9.1%) | **OR 1.88** (0.90 to 3.93) | **67 more per 1,000** (from 8 fewer to 191 more) | ⨁◯◯◯ Very low | IMPORTANT |
| **Incidence of pneumonia in the study population with an average age of ≤55 (follow-up: range 13 days to 90+ days; assessed with: medical records)** | | | | | | | | | | | | |
| 1 | observational studies | serious^k^ | not serious | not serious | not serious | publication bias strongly suspected^i^ | 8/30 (26.7%) | 5/29 (17.2%) | **OR 1.75** (0.50 to 6.14) | **95 more per 1,000** (from 78 fewer to 389 more) | ⨁◯◯◯ Very low | IMPORTANT |
| **Incidence of pneumonia in the study population with trauma patients accounted for <25% (follow-up: range 13 days to 90+ days; assessed with: medical records)** | | | | | | | | | | | | |
| 2 | observational studies | serious^k^ | not serious | not serious | serious^h^ | publication bias strongly suspected^i^ | 45/123 (36.6%) | 41/180 (22.8%) | **OR 1.89** (0.08 to 45.27) | **130 more per 1,000** (from 205 fewer to 703 more) | ⨁◯◯◯ Very low | IMPORTANT |
| **Incidence of pneumonia in the study population with burn patients accounted for 100% (follow-up: range 13 days to 90+ days; assessed with: medical records)** | | | | | | | | | | | | |
| 1 | observational studies | serious^k^ | not serious | not serious | not serious | publication bias strongly suspected^i^ | 8/30 (26.7%) | 5/29 (17.2%) | **OR 1.75** (0.50 to 6.14) | **95 more per 1,000** (from 78 fewer to 389 more) | ⨁◯◯◯ Very low | IMPORTANT |
| **Incidence of pneumonia in the study population with abdominal surgery patients accounted for 15% (follow-up: range 13 days to 90+ days; assessed with: medical records)** | | | | | | | | | | | | |
| 2 | observational studies | serious^k^ | not serious | not serious | not serious | publication bias strongly suspected^i^ | 32/232 (13.8%) | 22/376 (5.9%) | **OR 2.30** (0.81 to 6.48) | **67 more per 1,000** (from 11 fewer to 229 more) | ⨁◯◯◯ Very low | IMPORTANT |
| **Incidence of pneumonia in the study population with digestive diseases patients accounted for <10% (follow-up: range 13 days to 90+ days; assessed with: medical records)** | | | | | | | | | | | | |
| 2 | observational studies | serious^k^ | not serious | not serious | not serious | publication bias strongly suspected^i^ | 32/232 (13.8%) | 22/376 (5.9%) | **OR 2.30** (0.81 to 6.48) | **67 more per 1,000** (from 11 fewer to 229 more) | ⨁◯◯◯ Very low | IMPORTANT |
| **Incidence of pneumonia in the study population with sepsis patients accounted for <25% (follow-up: range 13 days to 90+ days; assessed with: medical records)** | | | | | | | | | | | | |
| 2 | observational studies | serious^k^ | not serious | not serious | not serious | publication bias strongly suspected^i^ | 17/215 (7.9%) | 23/390 (5.9%) | **OR 1.47** (0.52 to 4.17) | **25 more per 1,000** (from 27 fewer to 148 more) | ⨁◯◯◯ Very low | IMPORTANT |
| **Incidence of pneumonia in the study population with mechanical ventilation patients accounted for 100% (follow-up: range 13 days to 90+ days; assessed with: medical records)** | | | | | | | | | | | | |
| 2 | observational studies | serious^k^ | not serious | not serious | not serious | publication bias strongly suspected^i^ | 23/83 (27.7%) | 26/126 (20.6%) | **OR 1.51** (1.49 to 4.68) | **76 more per 1,000** (from 73 more to 343 more) | ⨁◯◯◯ Very low | IMPORTANT |
| **Incidence of pneumonia in the study population with average SOFA score of ≥8 (follow-up: range 13 days to 90+ days; assessed with: medical records)** | | | | | | | | | | | | |
| 1 | observational studies | serious^k^ | not serious | not serious | not serious | publication bias strongly suspected^i^ | 15/53 (28.3%) | 21/97 (21.6%) | **OR 1.43** (0.66 to 3.08) | **67 more per 1,000** (from 62 fewer to 243 more) | ⨁◯◯◯ Very low | IMPORTANT |
| **Incidence of pneumonia in the study population with average APACHE II score of ≥20 (follow-up: range 13 days to 90+ days; assessed with: medical records)** | | | | | | | | | | | | |
| 1 | observational studies | serious^k^ | not serious | not serious | not serious | publication bias strongly suspected^i^ | 15/53 (28.3%) | 21/97 (21.6%) | **OR 1.43** (0.66 to 3.08) | **67 more per 1,000** (from 62 fewer to 243 more) | ⨁◯◯◯ Very low | IMPORTANT |
| **Incidence of pneumonia in the study population with average APACHE II score of <20 (follow-up: range 13 days to 90+ days; assessed with: medical records)** | | | | | | | | | | | | |
| 1 | observational studies | serious^k^ | not serious | not serious | not serious | publication bias strongly suspected^i^ | 8/30 (26.7%) | 5/29 (17.2%) | **OR 1.75** (0.50 to 6.14) | **95 more per 1,000** (from 78 fewer to 389 more) | ⨁◯◯◯ Very low | IMPORTANT |
| **Length of ICU stay after redefining exposed cohort by defining FI according to GI symptoms cluster (follow-up: range 13 days to 60 days; assessed with: medical records; Scale from: 10 to 30)** | | | | | | | | | | | | |
| 19 | observational studies | serious^k^ | not serious | not serious | not serious | none | 9893 | 28270 | - | MD **4.23 days more** (2.92 more to 5.54 more) | ⨁◯◯◯ Very low | IMPORTANT |
| **Length of ICU stay after redefining exposed cohort by defining FI according to large-GRV-containing GI symptoms cluster (follow-up: range 13 days to 60 days; assessed with: medical records; Scale from: 10 to 30)** | | | | | | | | | | | | |
| 15 | observational studies | serious^k^ | not serious | not serious | not serious | none | 9564 | 27918 | - | MD **4.21 days more** (2.52 more to 5.9 more) | ⨁◯◯◯ Very low | IMPORTANT |
| **Length of ICU stay after redefining exposed cohort by defining FI according to only large GRV (follow-up: range 13 days to 60 days; assessed with: medical records; Scale from: 10 to 30)** | | | | | | | | | | | | |
| 3 | observational studies | serious^k^ | not serious | not serious | not serious | publication bias strongly suspected^i^ | 149 | 209 | - | MD **3.34 days more** (0.26 fewer to 6.95 more) | ⨁◯◯◯ Very low | IMPORTANT |
| **Length of ICU stay after redefining exposed cohort by defining FI according to EF insufficiency (follow-up: range 13 days to 60 days; assessed with: medical records; Scale from: 10 to 30)** | | | | | | | | | | | | |
| 3 | observational studies | not serious | not serious | not serious | serious^h^ | publication bias strongly suspected^i^ | 351 | 713 | - | MD **1.37 days more** (7.82 fewer to 10.55 more) | ⨁◯◯◯ Very low | IMPORTANT |
| **Length of ICU stay after redefining exposed cohort by defining FI according to GI symptoms cluster without large GRV (follow-up: range 20 days to 60 days; assessed with: medical records; Scale from: 13 to 30)** | | | | | | | | | | | | |
| 1 | observational studies | serious^k^ | not serious | not serious | not serious | publication bias strongly suspected^i^ | 180 | 143 | - | MD **6.4 days more** (4.11 more to 8.69 more) | ⨁◯◯◯ Very low | IMPORTANT |
| **Length of ICU stay after redefining exposed cohort by defining FI according to the number of GI symptoms ≥1 (no limit to the total number of candidate symptoms, which can range from 1-6) (follow-up: range 13 days to 60 days; assessed with: medical records; Scale from: 10 to 30)** | | | | | | | | | | | | |
| 13 | observational studies | serious^k^ | not serious | not serious | not serious | none | 9343 | 27747 | - | MD **4.04 days more** (2.12 more to 5.95 more) | ⨁◯◯◯ Very low | IMPORTANT |
| **Length of ICU stay after redefining exposed cohort by defining FI according to the number of GI symptoms ≥2 (no limit to the total number of candidate symptoms, which can range from 1-6) (follow-up: range 13 days to 60 days; assessed with: medical records; Scale from: 10 to 30)** | | | | | | | | | | | | |
| 2 | observational studies | serious^k^ | not serious | not serious | very serious^o^ | publication bias strongly suspected^i^ | 221 | 171 | - | MD **4.94 days more** (24.03 fewer to 33.91 more) | ⨁◯◯◯ Very low | IMPORTANT |
| **Length of ICU stay after redefining exposed cohort by defining FI according to the number of GI symptoms ≥1 (limit the total number of candidate symptoms to 4) (follow-up: range 13 days to 60 days; assessed with: medical records; Scale from: 10 to 30)** | | | | | | | | | | | | |
| 4 | observational studies | not serious | not serious | not serious | not serious | publication bias strongly suspected^i^ | 7893 | 24980 | - | MD **4.8 days more** (2.98 more to 6.63 more) | ⨁◯◯◯ Very low | IMPORTANT |
| **Length of ICU stay after redefining exposed cohort by defining FI according to the number of GI symptoms ≥1 (limit the total number of candidate symptoms to 5) (follow-up: range 13 days to 60 days; assessed with: medical records; Scale from: 10 to 30)** | | | | | | | | | | | | |
| 6 | observational studies | not serious | not serious | not serious | not serious | none | 1515 | 3074 | - | MD **1.58 days more** (2.59 fewer to 5.74 more) | ⨁⨁◯◯ Low | IMPORTANT |
| **Length of ICU stay after redefining exposed cohort by defining FI according to the number of GI symptoms ≥2 (limit the total number of candidate symptoms to 5) (follow-up: range 13 days to 60 days; assessed with: medical records; Scale from: 10 to 30)** | | | | | | | | | | | | |
| 2 | observational studies | serious^k^ | not serious | not serious | very serious^o^ | publication bias strongly suspected^i^ | 221 | 171 | - | MD **4.94 days more** (24.03 fewer to 33.91 more) | ⨁◯◯◯ Very low | IMPORTANT |
| **Length of ICU stay after redefining exposed cohort by defining FI according to large GRV (as one of the symptoms) of 500±50 ml (follow-up: range 13 days to 60 days; assessed with: medical records; Scale from: 10 to 30)** | | | | | | | | | | | | |
| 5 | observational studies | serious^k^ | not serious | not serious | not serious | none | 258 | 355 | - | MD **4.38 days more** (0.09 fewer to 8.86 more) | ⨁◯◯◯ Very low | IMPORTANT |
| **Length of ICU stay after redefining exposed cohort by defining FI according to large GRV (as one of the symptoms) of 75±50 ml (follow-up: range 13 days to 60 days; assessed with: medical records; Scale from: 10 to 30)** | | | | | | | | | | | | |
| 2 | observational studies | serious^k^ | not serious | not serious | extremely serious^o^ | publication bias strongly suspected^i^ | 35 | 46 | - | MD **2.67 days fewer** (52.84 fewer to 47.49 more) | ⨁◯◯◯ Very low | IMPORTANT |
| **Length of ICU stay after redefining exposed cohort by defining FI according to large GRV (as one of the symptoms) of 250±50 ml (follow-up: range 13 days to 60 days; Scale from: 10 to 30)** | | | | | | | | | | | | |
| 11 | observational studies | serious^k^ | not serious | not serious | serious^m^ | none | 2505 | 6206 | - | MD **3.17 days more** (1.11 more to 5.24 more) | ⨁◯◯◯ Very low | IMPORTANT |
| **Length of ICU stay after redefining exposed cohort by defining FI according to large GRV (as the only symptom) of 75±50 ml (follow-up: range 13 days to 60 days; assessed with: medical records; Scale from: 10 to 30)** | | | | | | | | | | | | |
| 1 | observational studies | serious^k^ | not serious | not serious | serious^h^ | publication bias strongly suspected^i^ | 29 | 32 | - | MD **1.4 days more** (6.76 fewer to 9.56 more) | ⨁◯◯◯ Very low | IMPORTANT |
| **Length of ICU stay after redefining exposed cohort by defining FI according to large GRV (as the only symptom) of 500±50 ml (follow-up: range 13 days to 60 days; assessed with: medical records; Scale from: 10 to 30)** | | | | | | | | | | | | |
| 1 | observational studies | serious^k^ | not serious | not serious | not serious | publication bias strongly suspected^i^ | 67 | 80 | - | MD **4.4 days more** (4.03 more to 4.77 more) | ⨁◯◯◯ Very low | IMPORTANT |
| **Length of ICU stay after redefining exposed cohort by defining FI according to large GRV (as the only symptom) of 250±50 ml (follow-up: range 13 days to 60 days; assessed with: medical records; Scale from: 10 to 30)** | | | | | | | | | | | | |
| 1 | observational studies | serious^k^ | not serious | not serious | serious^m^ | publication bias strongly suspected^i^ | 53 | 97 | - | MD **2.13 days more** (0.35 more to 3.91 more) | ⨁◯◯◯ Very low | IMPORTANT |
| **Length of ICU stay after redefining exposed cohort by defining FI according to the measured interval of GRV (as one of the symptoms) of 4 hours (follow-up: range 13 days to 60 days; assessed with: medical records; Scale from: 10 to 30)** | | | | | | | | | | | | |
| 7 | observational studies | serious^k^ | not serious | not serious | not serious | publication bias strongly suspected^i^ | 580 | 995 | - | MD **2.8 days more** (0.36 fewer to 5.96 more) | ⨁◯◯◯ Very low | IMPORTANT |
| **Length of ICU stay after redefining exposed cohort by defining FI according to the measured interval of GRV (as one of the symptoms) of 6 hours (follow-up: range 13 days to 60 days; assessed with: medical records; Scale from: 10 to 30)** | | | | | | | | | | | | |
| 3 | observational studies | serious^k^ | not serious | not serious | serious^h^ | publication bias strongly suspected^i^ | 412 | 250 | - | MD **6.09 days more** (0.36 fewer to 12.55 more) | ⨁◯◯◯ Very low | IMPORTANT |
| **Length of ICU stay after redefining exposed cohort by defining FI according to the measured interval of GRV (as the only symptom) of 4 hours (follow-up: range 13 days to 60 days; assessed with: medical records; Scale from: 10 to 30)** | | | | | | | | | | | | |
| 3 | observational studies | serious^k^ | not serious | not serious | not serious | publication bias strongly suspected^i^ | 149 | 209 | - | MD **3.34 days more** (0.26 fewer to 6.95 more) | ⨁◯◯◯ Very low | IMPORTANT |
| **Length of ICU stay after redefining exposed cohort by defining FI according to EF percentage of 80% (follow-up: range 13 days to 60 days; assessed with: medical records; Scale from: 10 to 30)** | | | | | | | | | | | | |
| 3 | observational studies | not serious | not serious | not serious | serious^h^ | publication bias strongly suspected^i^ | 351 | 713 | - | MD **1.37 days more** (7.82 fewer to 10.55 more) | ⨁◯◯◯ Very low | IMPORTANT |
| **Length of ICU stay in the study population with surgical patients accounted for >25% (follow-up: range 13 days to 60 days; assessed with: medical records; Scale from: 10 to 30)** | | | | | | | | | | | | |
| 3 | observational studies | serious^k^ | not serious | not serious | serious^m^ | publication bias strongly suspected^i^ | 4261 | 12082 | - | MD **5.42 days more** (1.73 more to 9.1 more) | ⨁◯◯◯ Very low | IMPORTANT |
| **Length of ICU stay in the study population with surgical patients accounted for 0% (follow-up: range 13 days to 60 days; assessed with: medical records; Scale from: 10 to 30)** | | | | | | | | | | | | |
| 11 | observational studies | not serious | not serious | not serious | serious^m^ | none | 702 | 836 | - | MD **3.09 days more** (0.66 more to 5.52 more) | ⨁◯◯◯ Very low | IMPORTANT |
| **Length of ICU stay in the study population with surgical patients accounted for 15-20% (follow-up: range 13 days to 60 days; assessed with: medical records; Scale from: 10 to 30)** | | | | | | | | | | | | |
| 4 | observational studies | serious^k^ | not serious | not serious | serious^h^ | publication bias strongly suspected^i^ | 973 | 1977 | - | MD **2.17 days more** (3.84 fewer to 8.18 more) | ⨁◯◯◯ Very low | IMPORTANT |
| **Length of ICU stay in the study population with male patients accounted for ≤65% (follow-up: range 13 days to 60 days; assessed with: medical records; Scale from: 10 to 30)** | | | | | | | | | | | | |
| 13 | observational studies | not serious | not serious | not serious | not serious | none | 9681 | 27929 | - | MD **4.39 days more** (2.99 more to 5.78 more) | ⨁⨁◯◯ Low | IMPORTANT |
| **Length of ICU stay in the study population with male patients accounted for >65% (follow-up: range 13 days to 60 days; assessed with: medical records; Scale from: 10 to 30)** | | | | | | | | | | | | |
| 9 | observational studies | serious^k^ | serious^l^ | not serious | not serious | publication bias strongly suspected^i^ | 563 | 1054 | - | MD **2.02 days more** (1.12 fewer to 5.15 more) | ⨁◯◯◯ Very low | IMPORTANT |
| **Length of ICU stay in the study population with an average age of >55 (follow-up: range 13 days to 60 days; assessed with: medical records; Scale from: 10 to 30)** | | | | | | | | | | | | |
| 14 | observational studies | not serious | not serious | not serious | serious^m^ | none | 9768 | 28474 | - | MD **2.96 days more** (1.32 more to 4.59 more) | ⨁◯◯◯ Very low | IMPORTANT |
| **Length of ICU stay in the study population with an average age of ≤55 (follow-up: range 13 days to 60 days; assessed with: medical records; Scale from: 10 to 30)** | | | | | | | | | | | | |
| 8 | observational studies | serious^k^ | not serious | not serious | not serious | publication bias strongly suspected^i^ | 476 | 509 | - | MD **5.52 days more** (3.48 more to 7.56 more) | ⨁◯◯◯ Very low | IMPORTANT |
| **Length of ICU stay in the study population with trauma patients accounted for <25% (follow-up: range 13 days to 60 days; assessed with: medical records; Scale from: 10 to 30)** | | | | | | | | | | | | |
| 4 | observational studies | serious^k^ | not serious | not serious | not serious | publication bias strongly suspected^i^ | 854 | 1609 | - | MD **3.8 days more** (0.25 fewer to 7.86 more) | ⨁◯◯◯ Very low | IMPORTANT |
| **Length of ICU stay in the study population with trauma patients accounted for 100% (follow-up: range 13 days to 60 days; assessed with: medical records; Scale from: 10 to 30)** | | | | | | | | | | | | |
| 4 | observational studies | serious^k^ | not serious | not serious | serious^h^ | publication bias strongly suspected^i^ | 181 | 280 | - | MD **3.9 days more** (3.73 fewer to 11.53 more) | ⨁◯◯◯ Very low | IMPORTANT |
| **Length of ICU stay in the study population with trauma patients accounted for 40-60% (follow-up: range 13 days to 60 days; assessed with: medical records; Scale from: 10 to 30)** | | | | | | | | | | | | |
| 1 | observational studies | not serious | not serious | not serious | not serious | publication bias strongly suspected^i^ | 95 | 50 | - | MD **7 days more** (6.51 more to 7.49 more) | ⨁◯◯◯ Very low | IMPORTANT |
| **Length of ICU stay in the study population with burn patients accounted for 5-10% (follow-up: range 13 days to 60 days; assessed with: medical records; Scale from: 10 to 30)** | | | | | | | | | | | | |
| 2 | observational studies | serious^k^ | not serious | not serious | not serious | publication bias strongly suspected^i^ | 250 | 167 | - | MD **7 days more** (6.35 more to 7.66 more) | ⨁◯◯◯ Very low | IMPORTANT |
| **Length of ICU stay in the study population with burn patients accounted for 100% (follow-up: range 13 days to 60 days; assessed with: medical records; Scale from: 10 to 30)** | | | | | | | | | | | | |
| 1 | observational studies | serious^k^ | not serious | not serious | serious^h^ | publication bias strongly suspected^i^ | 16 | 30 | - | MD **6 days more** (7.75 fewer to 19.75 more) | ⨁◯◯◯ Very low | IMPORTANT |
| **Length of ICU stay in the study population with abdominal surgery patients accounted for 15% (follow-up: range 13 days to 60 days; assessed with: medical records; Scale from: 10 to 30)** | | | | | | | | | | | | |
| 1 | observational studies | serious^k^ | not serious | not serious | not serious | publication bias strongly suspected^i^ | 70 | 83 | - | MD **8 days more** (2 more to 14 more) | ⨁◯◯◯ Very low | IMPORTANT |
| **Length of ICU stay in the study population with abdominal surgery patients accounted for 5% (follow-up: range 13 days to 60 days; assessed with: medical records; Scale from: 10 to 30)** | | | | | | | | | | | | |
| 1 | observational studies | not serious | not serious | not serious | serious^m^ | publication bias strongly suspected^i^ | 576 | 1312 | - | MD **3.1 days more** (1.57 more to 4.63 more) | ⨁◯◯◯ Very low | IMPORTANT |
| **Length of ICU stay in the study population with digestive diseases patients accounted for <10% (follow-up: range 13 days to 60 days; assessed with: medical records; Scale from: 10 to 30)** | | | | | | | | | | | | |
| 3 | observational studies | serious^k^ | not serious | not serious | not serious | publication bias strongly suspected^i^ | 808 | 1478 | - | MD **3.28 days more** (0.37 fewer to 6.94 more) | ⨁◯◯◯ Very low | IMPORTANT |
| **Length of ICU stay in the study population with sepsis patients accounted for 25-55% (follow-up: range 13 days to 60 days; assessed with: medical records; Scale from: 10 to 30)** | | | | | | | | | | | | |
| 2 | observational studies | not serious | serious^l^ | not serious | very serious^o^ | publication bias strongly suspected^i^ | 291 | 353 | - | MD **3.52 days more** (40.95 fewer to 47.99 more) | ⨁◯◯◯ Very low | IMPORTANT |
| **Length of ICU stay in the study population with sepsis patients accounted for 100% (follow-up: range 13 days to 60 days; assessed with: medical records; Scale from: 10 to 30)** | | | | | | | | | | | | |
| 3 | observational studies | serious^k^ | not serious | not serious | not serious | publication bias strongly suspected^k^ | 97 | 201 | - | MD **0.83 days fewer** (5.22 fewer to 3.55 more) | ⨁◯◯◯ Very low | IMPORTANT |
| **Length of ICU stay in the study population with sepsis patients accounted for <25% (follow-up: range 13 days to 60 days; assessed with: medical records; Scale from: 10 to 30)** | | | | | | | | | | | | |
| 3 | observational studies | serious^k^ | not serious | not serious | serious^m^ | publication bias strongly suspected^i^ | 791 | 1492 | - | MD **2.65 days more** (1.12 more to 4.18 more) | ⨁◯◯◯ Very low | IMPORTANT |
| **Length of ICU stay in the study population with mechanical ventilation patients accounted for 100% (follow-up: range 13 days to 60 days; assessed with: medical records; Scale from: 10 to 30)** | | | | | | | | | | | | |
| 6 | observational studies | not serious | not serious | not serious | not serious | publication bias strongly suspected^i^ | 4812 | 13429 | - | MD **2.53 days more** (1.76 fewer to 6.83 more) | ⨁◯◯◯ Very low | IMPORTANT |
| **Length of ICU stay in the study population with mechanical ventilation patients accounted for 75-95% (follow-up: range 13 days to 60 days; assessed with: medical records; Scale from: 10 to 30)** | | | | | | | | | | | | |
| 1 | observational studies | not serious | not serious | not serious | serious^m^ | publication bias strongly suspected^i^ | 106 | 312 | - | MD **1.5 days fewer** (2.77 fewer to 0.23 fewer) | ⨁◯◯◯ Very low | IMPORTANT |
| **Length of ICU stay in the study population with average SOFA score of <8 (follow-up: range 13 days to 60 days; assessed with: medical records; Scale from: 10 to 30)** | | | | | | | | | | | | |
| 2 | observational studies | serious^k^ | not serious | not serious | not serious | publication bias strongly suspected^i^ | 196 | 173 | - | MD **6.39 days more** (5.57 more to 7.21 more) | ⨁◯◯◯ Very low | IMPORTANT |
| **Length of ICU stay in the study population with average SOFA score of ≥8 (follow-up: range 13 days to 60 days; assessed with: medical records; Scale from: 10 to 30)** | | | | | | | | | | | | |
| 4 | observational studies | serious^k^ | not serious | not serious | not serious | publication bias strongly suspected^i^ | 446 | 580 | - | MD **0.86 days more** (1.21 fewer to 2.94 more) | ⨁◯◯◯ Very low | IMPORTANT |
| **Length of ICU stay in the study population with average APACHE II score of <20 (follow-up: range 13 days to 60 days; assessed with: medical records; Scale from: 10 to 30)** | | | | | | | | | | | | |
| 7 | observational studies | serious^k^ | not serious | not serious | not serious | publication bias strongly suspected^i^ | 676 | 956 | - | MD **0.3 days more** (3.27 fewer to 3.88 more) | ⨁◯◯◯ Very low | IMPORTANT |
| **Length of ICU stay in the study population with average APACHE II score of ≥20 (follow-up: range 13 days to 60 days; assessed with: medical records; Scale from: 10 to 30)** | | | | | | | | | | | | |
| 5 | observational studies | not serious | not serious | not serious | not serious | publication bias strongly suspected^i^ | 4806 | 13415 | - | MD **3.45 days more** (0.2 fewer to 7.09 more) | ⨁◯◯◯ Very low | IMPORTANT |
| **Length of hospital stay after redefining exposed cohort by defining FI according to GI symptoms cluster (follow-up: range 20 days to 60 days; assessed with: medical records; Scale from: 10 to 50)** | | | | | | | | | | | | |
| 11 | observational studies | serious^k^ | not serious | not serious | not serious | none | 5764 | 14783 | - | MD **5.03 days more** (2.06 more to 8 more) | ⨁◯◯◯ Very low | IMPORTANT |
| **Length of hospital stay after redefining exposed cohort by defining FI according to large-GRV-containing GI symptoms cluster (follow-up: range 20 days to 60 days; assessed with: medical records; Scale from: 10 to 50)** | | | | | | | | | | | | |
| 8 | observational studies | serious^k^ | not serious | not serious | serious^m^ | publication bias strongly suspected^i^ | 5488 | 14528 | - | MD **4.26 days more** (0.36 more to 8.15 more) | ⨁◯◯◯ Very low | IMPORTANT |
| **Length of hospital stay after redefining exposed cohort by defining FI according to only large GRV (follow-up: range 20 days to 60 days; assessed with: medical records; Scale from: 10 to 50)** | | | | | | | | | | | | |
| 2 | observational studies | serious^k^ | not serious | not serious | serious^h^ | publication bias strongly suspected^i^ | 96 | 112 | - | MD **7.68 days more** (10.7 fewer to 26.06 more) | ⨁◯◯◯ Very low | IMPORTANT |
| **Length of hospital stay after redefining exposed cohort by defining FI according to EF insufficiency (follow-up: range 20 days to 60 days; assessed with: medical records; Scale from: 10 to 50)** | | | | | | | | | | | | |
| 2 | observational studies | serious^k^ | not serious | not serious | serious^h^ | publication bias strongly suspected^i^ | 233 | 482 | - | MD **5.87 days more** (0.52 fewer to 12.27 more) | ⨁◯◯◯ Very low | IMPORTANT |
| **Length of hospital stay after redefining exposed cohort by defining FI according to GI symptoms cluster without large GRV (follow-up: range 20 days to 60 days; assessed with: medical records; Scale from: 10 to 50)** | | | | | | | | | | | | |
| 1 | observational studies | serious^k^ | not serious | not serious | serious^m^ | publication bias strongly suspected^i^ | 180 | 143 | - | MD **4.5 days more** (1.05 more to 7.95 more) | ⨁◯◯◯ Very low | IMPORTANT |
| **Length of hospital stay after redefining exposed cohort by defining FI according to the number of GI symptoms ≥1 (no limit to the total number of candidate symptoms, which can range from 1-6) (follow-up: range 20 days to 60 days; assessed with: medical records; Scale from: 10 to 50)** | | | | | | | | | | | | |
| 6 | observational studies | serious^k^ | not serious | not serious | not serious | publication bias strongly suspected^i^ | 5267 | 14357 | - | MD **3.79 days more** (2.59 more to 4.99 more) | ⨁◯◯◯ Very low | IMPORTANT |
| **Length of hospital stay after redefining exposed cohort by defining FI according to the number of GI symptoms ≥2 (no limit to the total number of candidate symptoms, which can range from 1-6) (follow-up: range 20 days to 60 days; assessed with: medical records; Scale from: 10 to 50)** | | | | | | | | | | | | |
| 2 | observational studies | serious^k^ | not serious | not serious | extremely serious^n^ | publication bias strongly suspected^n^ | 221 | 171 | - | MD **7.77 days more** (64 fewer to 79.54 more) | ⨁◯◯◯ Very low | IMPORTANT |
| **Length of hospital stay after redefining exposed cohort by defining FI according to the number of GI symptoms ≥1 (limit the total number of candidate symptoms to 4) (follow-up: range 20 days to 60 days; assessed with: medical records; Scale from: 10 to 50)** | | | | | | | | | | | | |
| 1 | observational studies | not serious | not serious | not serious | not serious | publication bias strongly suspected^i^ | 4036 | 11882 | - | MD **3.8 days more** (2.73 more to 4.87 more) | ⨁◯◯◯ Very low | IMPORTANT |
| **Length of hospital stay after redefining exposed cohort by defining FI according to the number of GI symptoms ≥1 (limit the total number of candidate symptoms to 5) (follow-up: range 20 days to 60 days; assessed with: medical records; Scale from: 10 to 50)** | | | | | | | | | | | | |
| 5 | observational studies | serious^k^ | not serious | not serious | not serious | publication bias strongly suspected^i^ | 1365 | 2544 | - | MD **4.01 days more** (2.65 more to 5.38 more) | ⨁◯◯◯ Very low | IMPORTANT |
| **Length of hospital stay after redefining exposed cohort by defining FI according to the number of GI symptoms ≥2 (limit the total number of candidate symptoms to 5) (follow-up: range 20 days to 60 days; assessed with: medical records; Scale from: 10 to 50)** | | | | | | | | | | | | |
| 2 | observational studies | serious^k^ | not serious | not serious | extremely serious^n^ | publication bias strongly suspected^i^ | 221 | 171 | - | MD **7.77 days more** (64 fewer to 79.54 more) | ⨁◯◯◯ Very low | IMPORTANT |
| **Length of hospital stay after redefining exposed cohort by defining FI according to large GRV (as one of the symptoms) of 75±50 ml (follow-up: range 20 days to 60 days; assessed with: medical records; Scale from: 10 to 50)** | | | | | | | | | | | | |
| 2 | observational studies | serious^k^ | serious^l^ | not serious | extremely serious^n^ | publication bias strongly suspected^i^ | 35 | 46 | - | MD **6.39 days more** (102.2 fewer to 114.98 more) | ⨁◯◯◯ Very low | IMPORTANT |
| **Length of hospital stay after redefining exposed cohort by defining FI according to large GRV (as one of the symptoms) of 250±50 ml (follow-up: range 20 days to 60 days; assessed with: medical records; Scale from: 10 to 50)** | | | | | | | | | | | | |
| 4 | observational studies | serious^k^ | not serious | not serious | not serious | publication bias strongly suspected^i^ | 945 | 1301 | - | MD **4.2 days more** (2.08 more to 6.32 more) | ⨁◯◯◯ Very low | IMPORTANT |
| **Length of hospital stay after redefining exposed cohort by defining FI according to large GRV (as one of the symptoms) of 500±50 ml (follow-up: range 20 days to 60 days; assessed with: medical records; Scale from: 10 to 50)** | | | | | | | | | | | | |
| 3 | observational studies | serious^k^ | serious^l^ | not serious | serious^h^ | publication bias strongly suspected^i^ | 172 | 242 | - | MD **6.45 days more** (12.39 fewer to 25.28 more) | ⨁◯◯◯ Very low | IMPORTANT |
| **Length of hospital stay after redefining exposed cohort by defining FI according to large GRV (as the only symptom) of 75±50 ml (follow-up: range 20 days to 60 days; assessed with: medical records; Scale from: 10 to 50)** | | | | | | | | | | | | |
| 1 | observational studies | serious^k^ | not serious | not serious | serious^m^ | publication bias strongly suspected^i^ | 29 | 32 | - | MD **15.2 days more** (0.45 more to 29.95 more) | ⨁◯◯◯ Very low | IMPORTANT |
| **Length of hospital stay after redefining exposed cohort by defining FI according to large GRV (as the only symptom) of 500±50 ml (follow-up: range 20 days to 60 days; assessed with: medical records; Scale from: 10 to 50)** | | | | | | | | | | | | |
| 1 | observational studies | serious^k^ | not serious | not serious | not serious | publication bias strongly suspected^i^ | 67 | 80 | - | MD **7.4 days more** (6.73 more to 8.07 more) | ⨁◯◯◯ Very low | IMPORTANT |
| **Length of hospital stay after redefining exposed cohort by defining FI according to the measured interval of GRV (as one of the symptoms) of 4 hours (follow-up: range 20 days to 60 days; assessed with: medical records; Scale from: 10 to 50)** | | | | | | | | | | | | |
| 3 | observational studies | serious^k^ | not serious | not serious | serious^m^ | publication bias strongly suspected^i^ | 155 | 200 | - | MD **10.63 days more** (1.43 more to 19.84 more) | ⨁◯◯◯ Very low | IMPORTANT |
| **Length of hospital stay after redefining exposed cohort by defining FI according to the measured interval of GRV (as one of the symptoms) of 6 hours (follow-up: range 20 days to 60 days; assessed with: medical records; Scale from: 10 to 50)** | | | | | | | | | | | | |
| 1 | observational studies | serious^k^ | not serious | not serious | serious^h^ | publication bias strongly suspected^i^ | 162 | 83 | - | MD **1.67 days more** (4.52 fewer to 7.86 more) | ⨁◯◯◯ Very low | IMPORTANT |
| **Length of hospital stay after redefining exposed cohort by defining FI according to the measured interval of GRV (as the only symptom) of 4 hours (follow-up: range 20 days to 60 days; assessed with: medical records; Scale from: 10 to 50)** | | | | | | | | | | | | |
| 2 | observational studies | serious^k^ | not serious | not serious | serious^h^ | publication bias strongly suspected^i^ | 96 | 112 | - | MD **7.68 days more** (10.7 fewer to 26.06 more) | ⨁◯◯◯ Very low | IMPORTANT |
| **Length of hospital stay after redefining exposed cohort by defining FI according to EF percentage of 80% (follow-up: range 20 days to 60 days; assessed with: medical records; Scale from: 10 to 50)** | | | | | | | | | | | | |
| 2 | observational studies | serious^k^ | not serious | not serious | serious^h^ | publication bias strongly suspected^i^ | 233 | 482 | - | MD **5.87 days more** (0.52 fewer to 12.27 more) | ⨁◯◯◯ Very low | IMPORTANT |
| **Length of hospital stay in the study population with surgical patients accounted for 0% (follow-up: range 20 days to 60 days; assessed with: medical records; Scale from: 10 to 50)** | | | | | | | | | | | | |
| 9 | observational studies | serious^k^ | not serious | not serious | not serious | publication bias strongly suspected^i^ | 782 | 996 | - | MD **5.88 days more** (2.02 more to 9.74 more) | ⨁◯◯◯ Very low | IMPORTANT |
| **Length of hospital stay in the study population with surgical patients accounted for 15-20% (follow-up: range 20 days to 60 days; assessed with: medical records; Scale from: 10 to 50)** | | | | | | | | | | | | |
| 1 | observational studies | not serious | not serious | not serious | not serious | publication bias strongly suspected^i^ | 576 | 1312 | - | MD **3.6 days more** (2.12 more to 5.08 more) | ⨁◯◯◯ Very low | IMPORTANT |
| **Length of hospital stay in the study population with surgical patients accounted for >25% (follow-up: range 20 days to 60 days; assessed with: medical records; Scale from: 10 to 50)** | | | | | | | | | | | | |
| 2 | observational studies | serious^k^ | not serious | not serious | serious^m^ | publication bias strongly suspected^i^ | 4188 | 11967 | - | MD **3.66 days more** (0.81 more to 6.51 more) | ⨁◯◯◯ Very low | IMPORTANT |
| **Length of hospital stay in the study population with male patients accounted for >65% (follow-up: range 20 days to 60 days; assessed with: medical records; Scale from: 10 to 50 )** | | | | | | | | | | | | |
| 5 | observational studies | serious^k^ | not serious | not serious | not serious | publication bias strongly suspected^i^ | 210 | 312 | - | MD **8.33 days more** (2.3 more to 14.36 more) | ⨁◯◯◯ Very low | IMPORTANT |
| **Length of hospital stay in the study population with male patients accounted for ≤65% (follow-up: range 20 days to 60 days; assessed with: medical records; Scale from: 10 to 50)** | | | | | | | | | | | | |
| 8 | observational studies | serious^k^ | not serious | not serious | not serious | publication bias strongly suspected^i^ | 5787 | 14953 | - | MD **4.17 days more** (2.58 more to 5.76 more) | ⨁◯◯◯ Very low | IMPORTANT |
| **Length of hospital stay in the study population with an average age of ≤55 (follow-up: range 20 days to 60 days; assessed with: medical records; Scale from: 10 to 50)** | | | | | | | | | | | | |
| 7 | observational studies | serious^k^ | not serious | not serious | not serious | publication bias strongly suspected^i^ | 546 | 781 | - | MD **7.17 days more** (3.28 more to 11.06 more) | ⨁◯◯◯ Very low | IMPORTANT |
| **Length of hospital stay in the study population with an average age of >55 (follow-up: range 20 days to 60 days; assessed with: medical records; Scale from: 10 to 50)** | | | | | | | | | | | | |
| 6 | observational studies | not serious | not serious | not serious | not serious | publication bias strongly suspected^i^ | 5451 | 14484 | - | MD **3.9 days more** (2.67 more to 5.14 more) | ⨁◯◯◯ Very low | IMPORTANT |
| **Length of hospital stay in the study population with trauma patients accounted for 100% (follow-up: range 20 days to 60 days; assessed with: medical records; Scale from: 10 to 50)** | | | | | | | | | | | | |
| 4 | observational studies | serious^k^ | not serious | not serious | serious^m^ | publication bias strongly suspected^i^ | 181 | 280 | - | MD **7.83 days more** (0.2 more to 15.46 more) | ⨁◯◯◯ Very low | IMPORTANT |
| **Length of hospital stay in the study population with trauma patients accounted for <25% (follow-up: range 20 days to 60 days; assessed with: medical records; Scale from: 10 to 50)** | | | | | | | | | | | | |
| 1 | observational studies | not serious | not serious | not serious | not serious | publication bias strongly suspected^i^ | 576 | 1312 | - | MD **3.6 days more** (2.12 more to 5.08 more) | ⨁◯◯◯ Very low | IMPORTANT |
| **Length of hospital stay in the study population with abdominal surgery patients accounted for 5% (follow-up: range 20 days to 60 days; assessed with: medical records; Scale from: 10 to 50)** | | | | | | | | | | | | |
| 1 | observational studies | not serious | not serious | not serious | not serious | publication bias strongly suspected^i^ | 576 | 1312 | - | MD **3.6 days more** (2.12 more to 5.08 more) | ⨁◯◯◯ Very low | IMPORTANT |
| **Length of hospital stay in the study population with digestive diseases patients accounted for <10% (follow-up: range 20 days to 60 days; assessed with: medical records; Scale from: 10 to 50)** | | | | | | | | | | | | |
| 2 | observational studies | serious^k^ | not serious | not serious | serious^h^ | publication bias strongly suspected^i^ | 738 | 1395 | - | MD **3.5 days more** (2.04 fewer to 9.03 more) | ⨁◯◯◯ Very low | IMPORTANT |
| **Length of hospital stay in the study population with digestive diseases patients accounted for 100% (follow-up: range 20 days to 60 days; assessed with: medical records; Scale from: 10 to 50)** | | | | | | | | | | | | |
| 1 | observational studies | serious^k^ | not serious | not serious | not serious | publication bias strongly suspected^i^ | 184 | 384 | - | MD **6.8 days more** (4.92 more to 8.68 more) | ⨁◯◯◯ Very low | IMPORTANT |
| **Length of hospital stay in the study population with sepsis patients accounted for <25% (follow-up: range 20 days to 60 days; assessed with: medical records; Scale from: 10 to 50)** | | | | | | | | | | | | |
| 2 | observational studies | serious^k^ | not serious | not serious | serious^h^ | publication bias strongly suspected^i^ | 738 | 1395 | - | MD **3.5 days more** (2.04 fewer to 9.03 more) | ⨁◯◯◯ Very low | IMPORTANT |
| **Length of hospital stay in the study population with sepsis patients accounted for 100% (follow-up: range 20 days to 60 days; assessed with: medical records; Scale from: 10 to 50)** | | | | | | | | | | | | |
| 1 | observational studies | serious^k^ | not serious | not serious | serious^h^ | publication bias strongly suspected^i^ | 46 | 74 | - | MD **2.3 days fewer** (8.12 fewer to 3.52 more) | ⨁◯◯◯ Very low | IMPORTANT |
| **Length of hospital stay in the study population with mechanical ventilation patients accounted for 100% (follow-up: range 20 days to 60 days; assessed with: medical records; Scale from: 10 to 50)** | | | | | | | | | | | | |
| 4 | observational studies | serious^k^ | not serious | not serious | serious^m^ | publication bias strongly suspected^i^ | 4664 | 13282 | - | MD **3.58 days more** (1.84 more to 5.32 more) | ⨁◯◯◯ Very low | IMPORTANT |
| **Length of hospital stay in the study population with average SOFA score of ≥8 (follow-up: range 20 days to 60 days; assessed with: medical records; Scale from: 10 to 50)** | | | | | | | | | | | | |
| 2 | observational studies | serious^k^ | not serious | not serious | serious^h^ | publication bias strongly suspected^i^ | 314 | 168 | - | MD **3.18 days more** (2.14 fewer to 8.51 more) | ⨁◯◯◯ Very low | IMPORTANT |
| **Length of hospital stay in the study population with average SOFA score of <8 (follow-up: range 20 days to 60 days; assessed with: medical records; Scale from: 10 to 50)** | | | | | | | | | | | | |
| 1 | observational studies | serious^k^ | not serious | not serious | serious^m^ | publication bias strongly suspected^i^ | 180 | 143 | - | MD **4.5 days more** (1.05 more to 7.95 more) | ⨁◯◯◯ Very low | IMPORTANT |
| **Length of hospital stay in the study population with average APACHE II score of <20 (follow-up: range 20 days to 60 days; assessed with: medical records; Scale from: 10 to 50)** | | | | | | | | | | | | |
| 3 | observational studies | serious^k^ | not serious | not serious | serious^h^ | publication bias strongly suspected^i^ | 352 | 481 | - | MD **4.32 days more** (5.19 fewer to 13.83 more) | ⨁◯◯◯ Very low | IMPORTANT |
| **Length of hospital stay in the study population with average APACHE II score of ≥20 (follow-up: range 20 days to 60 days; assessed with: medical records; Scale from: 10 to 50)** | | | | | | | | | | | | |
| 3 | observational studies | not serious | not serious | not serious | serious^m^ | publication bias strongly suspected^i^ | 4658 | 13268 | - | MD **3.6 days more** (0.92 more to 6.28 more) | ⨁◯◯◯ Very low | IMPORTANT |
| **Mechanical ventilation days after redefining exposed cohort by defining FI according to GI symptoms cluster (follow-up: range 13 days to 90+ days; assessed with: medical records; Scale from: 7 to 26)** | | | | | | | | | | | | |
| 7 | observational studies | serious^k^ | serious^l^ | not serious | not serious | publication bias strongly suspected^i^ | 349 | 442 | - | MD **3.01 days more** (1.21 fewer to 7.23 more) | ⨁◯◯◯ Very low | IMPORTANT |
| **Mechanical ventilation days after redefining exposed cohort by defining FI according to large-GRV-containing GI symptoms cluster (follow-up: range 13 days to 90+ days; assessed with: medical records; Scale from: 7 to 26)** | | | | | | | | | | | | |
| 3 | observational studies | serious^k^ | not serious | not serious | serious^h^ | publication bias strongly suspected^i^ | 57 | 141 | - | MD **3.24 days more** (8.97 fewer to 15.44 more) | ⨁◯◯◯ Very low | IMPORTANT |
| **Mechanical ventilation days after redefining exposed cohort by defining FI according to only large GRV (follow-up: range 13 days to 90+ days; assessed with: medical records; Scale from: 7 to 26)** | | | | | | | | | | | | |
| 3 | observational studies | serious^k^ | not serious | not serious | not serious | publication bias strongly suspected^i^ | 112 | 158 | - | MD **1.67 days more** (0.94 fewer to 4.28 more) | ⨁◯◯◯ Very low | IMPORTANT |
| **Mechanical ventilation days after redefining exposed cohort by defining FI according to EF insufficiency (follow-up: range 13 days to 90+ days; assessed with: medical records; Scale from: 7 to 26)** | | | | | | | | | | | | |
| 1 | observational studies | not serious | not serious | not serious | not serious | publication bias strongly suspected^i^ | 196 | 303 | - | MD **0.8 days more** (0.28 fewer to 1.88 more) | ⨁◯◯◯ Very low | IMPORTANT |
| **Mechanical ventilation days after redefining exposed cohort by defining FI according to GI symptoms cluster without large GRV (follow-up: range 13 days to 90+ days; assessed with: medical records; Scale from: 7 to 26)** | | | | | | | | | | | | |
| 1 | observational studies | serious^k^ | not serious | not serious | not serious | publication bias strongly suspected^i^ | 180 | 143 | - | MD **9.3 days more** (6.92 more to 11.68 more) | ⨁◯◯◯ Very low | IMPORTANT |
| **Mechanical ventilation days after redefining exposed cohort by defining FI according to the number of GI symptoms ≥1 (no limit to the total number of candidate symptoms, which can range from 1-6) (follow-up: range 13 days to 90+ days; assessed with: medical records; Scale from: 7 to 26)** | | | | | | | | | | | | |
| 3 | observational studies | serious^k^ | not serious | not serious | serious^h^ | publication bias strongly suspected^i^ | 57 | 141 | - | MD **3.24 days more** (8.97 fewer to 15.44 more) | ⨁◯◯◯ Very low | IMPORTANT |
| **Mechanical ventilation days after redefining exposed cohort by defining FI according to the number of GI symptoms ≥1 (limit the total number of candidate symptoms to 5) (follow-up: range 13 days to 90+ days; assessed with: medical records; Scale from: 7 to 26)** | | | | | | | | | | | | |
| 3 | observational studies | serious^k^ | serious^l^ | not serious | serious^h^ | publication bias strongly suspected^i^ | 382 | 460 | - | MD **3.52 days more** (11.34 fewer to 18.38 more) | ⨁◯◯◯ Very low | IMPORTANT |
| **Mechanical ventilation days after redefining exposed cohort by defining FI according to large GRV (as one of the symptoms) of 75±50 ml (follow-up: range 13 days to 90+ days; assessed with: medical records; Scale from: 7 to 26)** | | | | | | | | | | | | |
| 2 | observational studies | serious^k^ | not serious | not serious | not serious | publication bias strongly suspected^i^ | 35 | 46 | - | MD **2.87 days fewer** (3.44 fewer to 2.31 fewer) | ⨁◯◯◯ Very low | IMPORTANT |
| **Mechanical ventilation days after redefining exposed cohort by defining FI according to large GRV (as one of the symptoms) of 500±50 ml (follow-up: range 13 days to 90+ days; assessed with: medical records; Scale from: 7 to 26)** | | | | | | | | | | | | |
| 1 | observational studies | serious^k^ | not serious | not serious | serious^m^ | publication bias strongly suspected^i^ | 16 | 30 | - | MD **7.7 days more** (1.53 more to 13.87 more) | ⨁◯◯◯ Very low | IMPORTANT |
| **Mechanical ventilation days after redefining exposed cohort by defining FI according to large GRV (as one of the symptoms) of 250±50 ml (follow-up: range 13 days to 90+ days; assessed with: medical records; Scale from: 7 to 26)** | | | | | | | | | | | | |
| 5 | observational studies | serious^k^ | not serious | not serious | not serious | publication bias strongly suspected^i^ | 494 | 669 | - | MD **2.95 days more** (1.69 fewer to 7.6 more) | ⨁◯◯◯ Very low | IMPORTANT |
| **Mechanical ventilation days after redefining exposed cohort by defining FI according to large GRV (as the only symptom) of 75±50 ml (follow-up: range 13 days to 90+ days; assessed with: medical records; Scale from: 7 to 26)** | | | | | | | | | | | | |
| 1 | observational studies | serious^k^ | not serious | not serious | serious^h^ | publication bias strongly suspected^i^ | 29 | 32 | - | MD **2.9 days fewer** (10.19 fewer to 4.39 more) | ⨁◯◯◯ Very low | IMPORTANT |
| **Mechanical ventilation days after redefining exposed cohort by defining FI according to large GRV (as the only symptom) of 250±50 ml (follow-up: range 13 days to 90+ days; assessed with: medical records; Scale from: 7 to 26)** | | | | | | | | | | | | |
| 2 | observational studies | serious^k^ | not serious | not serious | not serious | publication bias strongly suspected^i^ | 83 | 126 | - | MD **1.8 days more** (2.71 fewer to 6.31 more) | ⨁◯◯◯ Very low | IMPORTANT |
| **Mechanical ventilation days after redefining exposed cohort by defining FI according to the measured interval of GRV (as one of the symptoms) of 4 hours (follow-up: range 13 days to 90+ days; assessed with: medical records; Scale from: 7 to 26)** | | | | | | | | | | | | |
| 3 | observational studies | serious^k^ | not serious | not serious | not serious | publication bias strongly suspected^k^ | 278 | 432 | - | MD **1.21 days more** (1.29 fewer to 3.71 more) | ⨁◯◯◯ Very low | IMPORTANT |
| **Mechanical ventilation days after redefining exposed cohort by defining FI according to the measured interval of GRV (as the only symptom) of 4 hours (follow-up: range 13 days to 90+ days; assessed with: medical records; Scale from: 7 to 26)** | | | | | | | | | | | | |
| 2 | observational studies | serious^k^ | not serious | not serious | very serious^o^ | publication bias strongly suspected^i^ | 82 | 129 | - | MD **0.9 days more** (24.26 fewer to 26.05 more) | ⨁◯◯◯ Very low | IMPORTANT |
| **Mechanical ventilation days after redefining exposed cohort by defining FI according to EF percentage of 80% (follow-up: range 13 days to 90+ days; assessed with: medical records; Scale from: 7 to 26)** | | | | | | | | | | | | |
| 1 | observational studies | not serious | not serious | not serious | not serious | publication bias strongly suspected^i^ | 196 | 303 | - | MD **0.8 days more** (0.28 fewer to 1.88 more) | ⨁◯◯◯ Very low | IMPORTANT |
| **Mechanical ventilation days in the study population with surgical patients accounted for 0% (follow-up: range 13 days to 90+ days; assessed with: medical records; Scale from: 7 to 26)** | | | | | | | | | | | | |
| 7 | observational studies | serious^k^ | not serious | not serious | not serious | publication bias strongly suspected^i^ | 349 | 442 | - | MD **3.01 days more** (1.21 fewer to 7.23 more) | ⨁◯◯◯ Very low | IMPORTANT |
| **Mechanical ventilation days in the study population with surgical patients accounted for 15-20% (follow-up: range 13 days to 90+ days; assessed with: medical records; Scale from: 7 to 26)** | | | | | | | | | | | | |
| 1 | observational studies | not serious | not serious | not serious | not serious | publication bias strongly suspected^i^ | 196 | 303 | - | MD **0.8 days more** (0.28 fewer to 1.88 more) | ⨁◯◯◯ Very low | IMPORTANT |
| **Mechanical ventilation days in the study population with male patients accounted for >65% (follow-up: range 13 days to 90+ days; assessed with: medical records; Scale from: 7 to 26)** | | | | | | | | | | | | |
| 6 | observational studies | serious^k^ | not serious | not serious | not serious | publication bias strongly suspected^i^ | 312 | 505 | - | MD **0.9 days more** (0.55 fewer to 2.35 more) | ⨁◯◯◯ Very low | IMPORTANT |
| **Mechanical ventilation days in the study population with male patients accounted for ≤65% (follow-up: range 13 days to 90+ days; assessed with: medical records; Scale from: 7 to 26)** | | | | | | | | | | | | |
| 2 | observational studies | serious^k^ | not serious | not serious | very serious^o^ | publication bias strongly suspected^i^ | 233 | 240 | - | MD **5.55 days more** (41.27 fewer to 52.36 more) | ⨁◯◯◯ Very low | IMPORTANT |
| **Mechanical ventilation days in the study population with an average age of ≤55 (follow-up: range 13 days to 90+ days; assessed with: medical records; Scale from: 7 to 26)** | | | | | | | | | | | | |
| 4 | observational studies | serious^k^ | not serious | not serious | serious^h^ | publication bias strongly suspected^i^ | 81 | 105 | - | MD **1.39 days more** (6.24 fewer to 9.02 more) | ⨁◯◯◯ Very low | IMPORTANT |
| **Mechanical ventilation days in the study population with an average age of >55 (follow-up: range 13 days to 90+ days; assessed with: medical records; Scale from: 7 to 26)** | | | | | | | | | | | | |
| 4 | observational studies | serious^k^ | not serious | not serious | serious^h^ | publication bias strongly suspected^i^ | 464 | 640 | - | MD **3.44 days more** (3.04 fewer to 9.92 more) | ⨁◯◯◯ Very low | IMPORTANT |
| **Mechanical ventilation days in the study population with trauma patients accounted for 100% (follow-up: range 13 days to 90+ days; assessed with: medical records; Scale from: 7 to 26)** | | | | | | | | | | | | |
| 1 | observational studies | serious^k^ | not serious | not serious | serious^h^ | publication bias strongly suspected^i^ | 6 | 14 | - | MD **2.8 days fewer** (14.78 fewer to 9.18 more) | ⨁◯◯◯ Very low | IMPORTANT |
| **Mechanical ventilation days in the study population with trauma patients accounted for <25% (follow-up: range 13 days to 90+ days; assessed with: medical records; Scale from: 7 to 26)** | | | | | | | | | | | | |
| 1 | observational studies | serious^k^ | not serious | not serious | serious^m^ | publication bias strongly suspected^i^ | 53 | 97 | - | MD **1.93 days more** (0.6 more to 3.26 more) | ⨁◯◯◯ Very low | IMPORTANT |
| **Mechanical ventilation days in the study population with burn patients accounted for 100% (follow-up: range 13 days to 90+ days; Scale from: 7 to 26)** | | | | | | | | | | | | |
| 2 | observational studies | serious^k^ | not serious | not serious | very serious^o^ | publication bias strongly suspected^i^ | 46 | 59 | - | MD **3.8 days more** (39.66 fewer to 47.26 more) | ⨁◯◯◯ Very low | IMPORTANT |
| **Mechanical ventilation days in the study population with sepsis patients accounted for 100% (follow-up: range 13 days to 90+ days; assessed with: medical records; Scale from: 7 to 26)** | | | | | | | | | | | | |
| 2 | observational studies | serious^k^ | not serious | not serious | very serious^o^ | publication bias strongly suspected^i^ | 51 | 127 | - | MD **4.5 days more** (36.16 fewer to 45.16 more) | ⨁◯◯◯ Very low | IMPORTANT |
| **Mechanical ventilation days in the study population with sepsis patients accounted for <25% (follow-up: range 13 days to 90+ days; assessed with: medical records; Scale from: 7 to 26)** | | | | | | | | | | | | |
| 1 | observational studies | serious^k^ | not serious | not serious | serious^m^ | publication bias strongly suspected^i^ | 53 | 97 | - | MD **1.93 days more** (0.6 more to 3.26 more) | ⨁◯◯◯ Very low | IMPORTANT |
| **Mechanical ventilation days in the study population with sepsis patients accounted for 25-55% (follow-up: range 13 days to 90+ days; assessed with: medical records; Scale from: 7 to 26)** | | | | | | | | | | | | |
| 1 | observational studies | not serious | not serious | not serious | not serious | publication bias strongly suspected^i^ | 196 | 303 | - | MD **0.8 days more** (0.28 fewer to 1.88 more) | ⨁◯◯◯ Very low | IMPORTANT |
| **Mechanical ventilation days in the study population with mechanical ventilation patients accounted for 100% (follow-up: range 13 days to 90+ days; assessed with: medical records; Scale from: 7 to 26)** | | | | | | | | | | | | |
| 3 | observational studies | serious^k^ | not serious | not serious | not serious | publication bias strongly suspected^i^ | 89 | 140 | - | MD **1.76 days more** (0.05 fewer to 3.56 more) | ⨁◯◯◯ Very low | IMPORTANT |
| **Mechanical ventilation days in the study population with average SOFA score of <8 (follow-up: range 13 days to 90+ days; assessed with: medical records; Scale from: 7 to 26)** | | | | | | | | | | | | |
| 2 | observational studies | serious^k^ | not serious | not serious | not serious | publication bias strongly suspected^i^ | 196 | 173 | - | MD **9.09 days more** (2.26 more to 15.92 more) | ⨁◯◯◯ Very low | IMPORTANT |
| **Mechanical ventilation days in the study population with average SOFA score of ≥8 (follow-up: range 13 days to 90+ days; assessed with: medical records; Scale from: 7 to 26)** | | | | | | | | | | | | |
| 3 | observational studies | serious^k^ | not serious | not serious | not serious | publication bias strongly suspected^i^ | 284 | 497 | - | MD **1.29 days more** (0.39 fewer to 2.97 more) | ⨁◯◯◯ Very low | IMPORTANT |
| **Mechanical ventilation days in the study population with average APACHE II score of <20 (follow-up: range 13 days to 90+ days; assessed with: medical records; Scale from: 7 to 26)** | | | | | | | | | | | | |
| 5 | observational studies | serious^k^ | not serious | not serious | not serious | publication bias strongly suspected^i^ | 283 | 473 | - | MD **0.97 days more** (0.64 fewer to 2.58 more) | ⨁◯◯◯ Very low | IMPORTANT |
| **Mechanical ventilation days in the study population with average APACHE II score of ≥20 (follow-up: range 13 days to 90+ days; assessed with: medical records; Scale from: 7 to 26)** | | | | | | | | | | | | |
| 1 | observational studies | serious^k^ | not serious | not serious | serious^m^ | publication bias strongly suspected^i^ | 53 | 97 | - | MD **1.93 days more** (0.6 more to 3.26 more) | ⨁◯◯◯ Very low | IMPORTANT |

**CI:** confidence interval; **MD:** mean difference; **OR:** odds ratio

**Explanations**

a. FI, feeding intolerance

b. including 28- and 30-day mortality

c. ICU, intensive care unit

d. the number of cohorts exceeds the total number of included studies because some studies provided one more different FI definitions-based independent cohorts of eligible data for pooling

e. when multiple non-independent mortalities were provided in the same study, select the one with the longest follow-up time or with the largest total number of deaths

f. including 50- and 60-day mortality

g. including 90- or more day mortality

h. with 95% CI containing zero effect value and a wide range (serious imprecision, 95% CI range exceeds 10 times the adverse risk/more days but not reaches 50)

i. with a number of studies of less than 10

j. including aspiration pneumonia and ventilator-associated pneumonia

k. with a mean score of risk of bias no more than 6 using the Newcastle-Ottawa Scale

l. with high heterogeneity and obvious directional differences between studies

m. with marginal significant results regarding the clinical significance (serious imprecision, judged by a small effect threshold with 0.8 or 1.2 times the adverse risk, or 2 fewer or 2 more days rather than the usual thresholds of 1 time the adverse risk or 0 days)

n. with 95% CI containing zero effect value and a huge wide range (extremely serious imprecision, 95% CI range exceeds 100 times the adverse risk/more days)

o. with 95% CI containing zero effect value and a considerable wide range (very serious imprecision, 95% CI range exceeds 50 times the adverse risk/more days but not reaches 100)
