# Supplementary material for: Different definitions of feeding intolerance and their associations with outcomes of critically ill adults receiving enteral nutrition: a systematic review and meta-analysis
Source: J Intensive Care. 2023 Jul 5;11:29. doi: 10.1186/s40560-023-00674-3 (PMC10320932; doi:10.1186/s40560-023-00674-3)
Supplement: Supplementary file 4 — Additional file 4. Fig S1: Subgroup analyses for hospital/long-term mortality, and incidence of pneumonia, as well as length of hospital stay, and mechanical ventilation days according to characteristic levels of the study patients regardless of the kinds of FI definitions. [file 40560_2023_674_MOESM4_ESM.docx]

# Fig S1: Subgroup analyses for hospital/long-term mortality, and incidence of pneumonia, as well as length of hospital stay, and mechanical ventilation days according to characteristic levels of the study patients regardless of the kinds of FI definitions


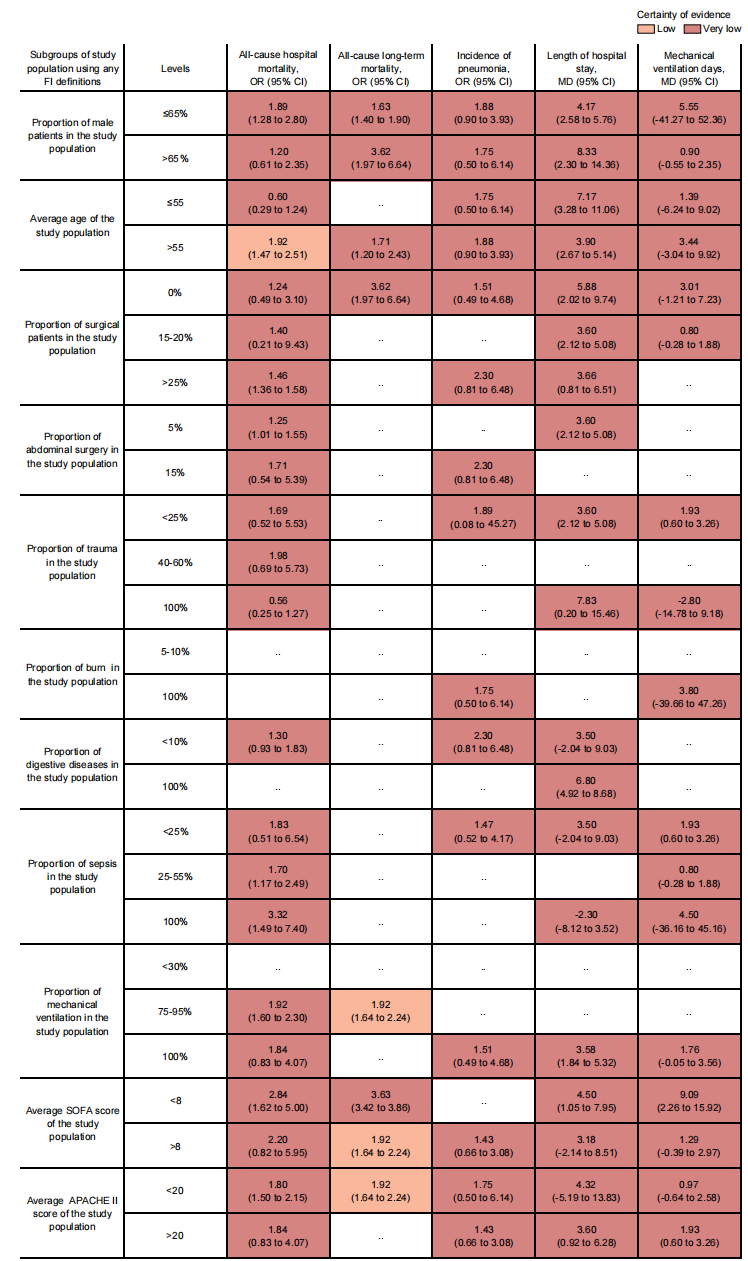


The certainty of the evidence was rated by the Grading of Recommendations Assessment, Development, and Evaluation criteria. FI=feeding intolerance, SOFA=Sequential Organ Failure Assessment, APACHE II=Acute Physiology, and Chronic Health Evaluation II, OR=odds ratio, MD=mean difference, CI=confidence interval.
