# Supplementary material for: Different definitions of feeding intolerance and their associations with outcomes of critically ill adults receiving enteral nutrition: a systematic review and meta-analysis
Source: J Intensive Care. 2023 Jul 5;11:29. doi: 10.1186/s40560-023-00674-3 (PMC10320932; doi:10.1186/s40560-023-00674-3)
Supplement: Supplementary file 5 — Additional file 5. Table S4: Egger’s test results for main outcomes. [file 40560_2023_674_MOESM5_ESM.docx]

# Table S4: Egger’s test results for main outcomes

| **Serial number** | **Outcomes** | **Number of studies** | **Intercept** | **t (95% CI)** | ***p* value** |
| --- | --- | --- | --- | --- | --- |
| 1 | All-cause hospital mortality | 16 | 0.32 | 0.56(-0.79 to 1.42) | 0.59 |
| 2 | All-cause long-term mortality | 5 | 2.99 | 0.89(-3.67 to 9.65) | 0.45 |
| 3 | All-cause ICU mortality | 17 | 0.29 | 0.49(-0.87 to 1.45) | 0.64 |
| 4 | All-cause mortality | 28 | 0.49 | 1.13(-0.37 to 1.34) | 0.28 |
| 5 | Pneumonia rate | 4 | -0.27 | -0.28(-2.13 to 1.6) | 0.81 |
| 6 | Length of ICU stay | 22 | -1.98 | -1.65(-4.34 to 0.39) | 0.12 |
| 7 | Length of hospital stay | 13 | -3.07 | -1.26(-7.87 to 1.74) | 0.24 |
| 8 | Mechanical ventilation days | 8 | 0.68 | 0.44(-2.37 to 3.71) | 0.68 |
