# Supplementary figures and images for: Different definitions of feeding intolerance and their associations with outcomes of critically ill adults receiving enteral nutrition: a systematic review and meta-analysis
Source: J Intensive Care. 2023 Jul 5;11:29. doi: 10.1186/s40560-023-00674-3 (PMC10320932; doi:10.1186/s40560-023-00674-3)

# Fig S2: Funnel plots for main outcome indicators


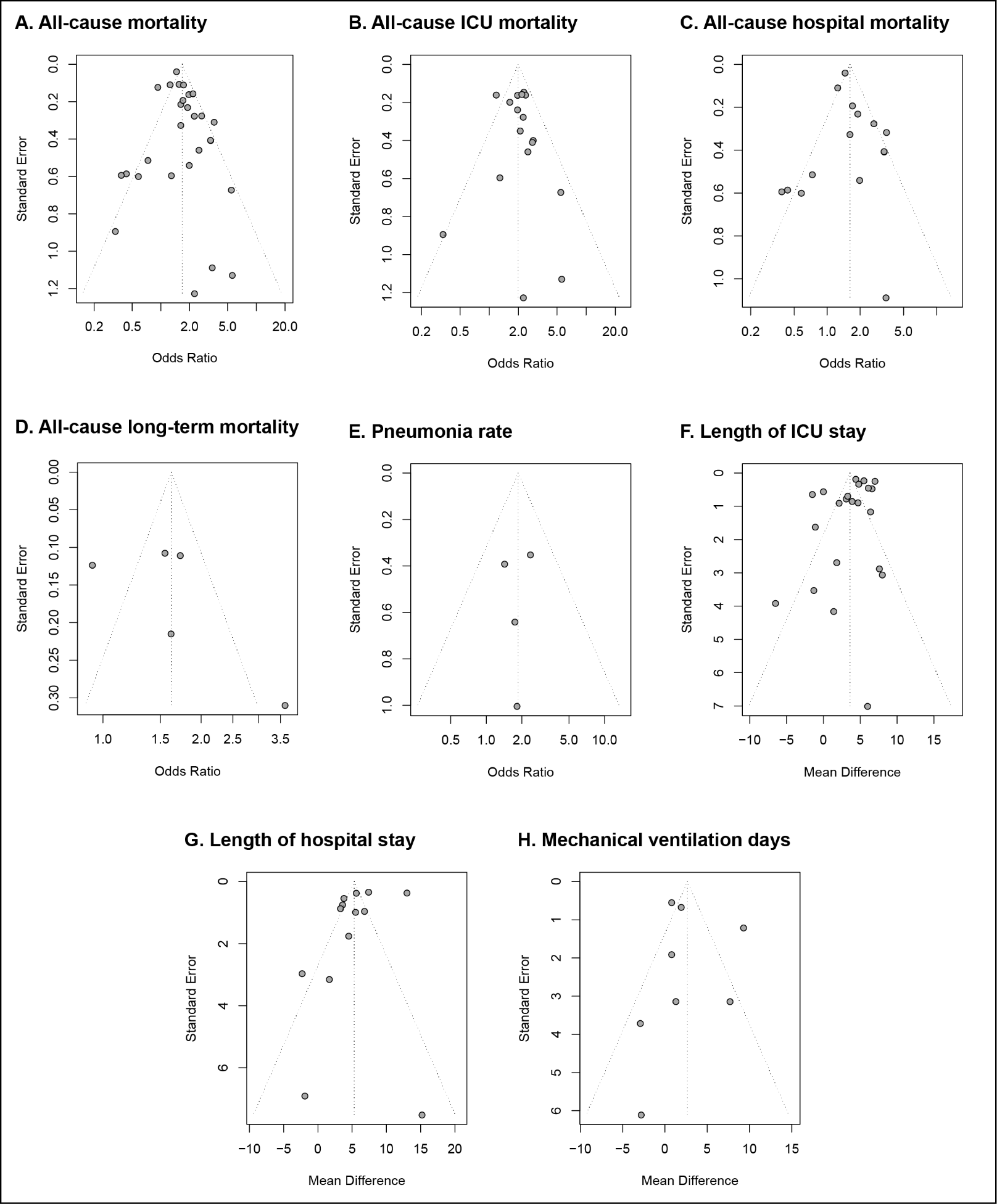

Supplement: Supplementary file 6 — Additional file 6. Fig S2: Funnel plots for main outcome indicators. [file 40560_2023_674_MOESM6_ESM.docx]
