# Supplementary material for: Different definitions of feeding intolerance and their associations with outcomes of critically ill adults receiving enteral nutrition: a systematic review and meta-analysis
Source: J Intensive Care. 2023 Jul 5;11:29. doi: 10.1186/s40560-023-00674-3 (PMC10320932; doi:10.1186/s40560-023-00674-3)
Supplement: Supplementary file 7 — Additional file 7. Table S5: Risk of bias of included studies according to pooled indicators [file 40560_2023_674_MOESM7_ESM.docx]

# Table S5: Risk of bias of included studies according to pooled indicators

| **Author, Year** | **Outcome** | | **Risk of bias** | | | | | | | | | | | | | | | | | |
| --- | --- | --- | --- | --- | --- | --- | --- | --- | --- | --- | --- | --- | --- | --- | --- | --- | --- | --- | --- | --- |
|  | **Definition of the exposed cohort** | **Outcome indicators** | **Representativeness of the exposed cohort** | | **Selection of the non-exposed cohort** | | **Ascertainment of exposure** | | **Demonstration that the outcome of interest was not present at the start of the study** | | **Comparability of cohorts based on the design or analysis** | | **Assessment of outcome** | | **Was follow-up long enough for outcomes to occur? (follow-up days)** | | **Adequacy of follow-up of cohorts (follow-up rates)** | | **Total score** | **Risk level** |
|  |  |  | **Score** | **Explanation** | **Score** | **Explanation** | **Score** | **Explanation** | **Score** | **Explanation** | **Score** | **Explanation** | **Score** | **Explanation** | **Score** | **Explanation** | **Score** | **Explanation** |  |  |
| Mentec et al, 2001 | Large-GRV-containing GI symptoms cluster | Pneumonia | 1 | Somewhat representative of the average prevalence of critically ill patients | 1 | Drawn from the same wards as the exposed cohort | 1 | Nursing and medical records | 0 | No clear description or uncertain | 1 | Control for simplified acute physiology score II | 1 | Reliable medical records | 0 | >=20 | 1 | 1.0 | 6 | Some concerns |
|  |  | All-cause ICU mortality | 1 |  | 1 |  | 1 |  | 1 | No mortality at the start of the study | 1 |  | 1 |  | 0 |  | 1 |  | 7 | Some concerns |
|  |  | All-cause hospital mortality | 1 |  | 1 |  | 1 |  | 1 |  | 1 |  | 1 |  | 0 |  | 1 |  | 7 | Some concerns |
|  |  | Length of ICU stay, Days | 1 |  | 1 |  | 1 |  | 0 | No clear description or uncertain | 1 |  | 1 |  | 0 |  | 1 |  | 6 | Some concerns |
| Brown et al, 2003 |  | Mechanical ventilation days | 0 | Select patients with specific diseases (trauma) | 1 |  | 1 |  | 0 |  | 0 | No specific control for any factors | 1 |  | 1 | >=30 | 1 |  | 5 | High |
|  |  | Length of ICU stay, Days | 0 |  | 1 |  | 1 |  | 0 |  | 0 |  | 1 |  | 1 |  | 1 |  | 5 | High |
|  |  | Length of hospital stay, Days | 0 |  | 1 |  | 1 |  | 0 |  | 0 |  | 1 |  | 1 |  | 1 |  | 5 | High |
| Nguyen&Ching et al, 2007 |  | Length of ICU stay, Days | 1 | Somewhat representative of the average prevalence of critically ill patients | 1 |  | 1 |  | 0 |  | 2 | To be matched for age, Gender, Body mass index, and admission Acute Physiology and Chronic Health Evaluation APACHE II score, and no significant difference in other important factors between groups | 1 |  | 0 | >=18 | 1 |  | 7 | Some concerns |
| Nguyen&Lam et al, 2007 |  |  | 1 |  | 1 |  | 1 |  | 0 |  | 0 | No specific control for any factors | 1 |  | 0 | >=20 | 1 |  | 5 | High |
| Stevens et al, 2008 | Only large GRV >75 mL×2 times | All-cause mortality | 0 | Select patients with specific diseases (therapeutic pentobarbital coma) | 1 |  | 1 |  | 1 | No mortality at the start of the study | 0 |  | 1 |  | 1 | >=36 | 1 |  | 6 | Some concerns |
|  |  | Mechanical ventilation days | 0 |  | 1 |  | 1 |  | 0 | No clear description or uncertain | 0 |  | 1 |  | 1 |  | 1 |  | 5 | High |
|  |  | Length of ICU stay, Days | 0 |  | 1 |  | 1 |  | 0 |  | 0 |  | 1 |  | 1 |  | 1 |  | 5 | High |
|  |  | Length of hospital stay, Days | 0 |  | 1 |  | 1 |  | 0 |  | 0 |  | 1 |  | 1 |  | 1 |  | 5 | High |
| Bejarano et al, 2013 | EF insufficiency in 3 feeding days | All-cause hospital mortality | 1 | Somewhat representative of the average prevalence of critically ill patients | 1 |  | 1 |  | 1 | No mortality at the start of the study | 0 |  | 1 |  | 0 | NA | 1 |  | 6 | Some concerns |
| Lavrentieva et al, 2014 | Large-GRV-containing GI symptoms cluster | All-cause ICU mortality | 0 | Select patients with specific diseases (burns) | 1 |  | 1 |  | 1 |  | 0 |  | 1 |  | 1 | >=30 | 1 |  | 6 | Some concerns |
|  |  | Mechanical ventilation days (survivors only) | 0 |  | 1 |  | 1 |  | 0 | No clear description or uncertain | 0 |  | 1 |  | 1 |  | 1 |  | 5 | High |
|  |  | Length of ICU stay (survivors only), Days | 0 |  | 1 |  | 1 |  | 0 |  | 0 |  | 1 |  | 1 |  | 1 |  | 5 | High |
| Gungabissoon et al, 2015 |  | All-cause 60-day mortality | 1 | Somewhat representative of the average prevalence of critically ill patients | 1 |  | 1 |  | 1 | No mortality at the start of the study | 1 | Possible control for important factors | 1 | Record linkage | 1 | 60 | 1 |  | 8 | Low |
|  |  | Length of ICU stay, Days | 1 |  | 1 |  | 1 |  | 0 |  | 1 |  | 1 |  | 1 |  | 1 |  | 7 | Some concerns |
|  |  | Length of hospital stay (survivors only), Days | 1 |  | 1 |  | 1 |  | 0 |  | 1 |  | 1 |  | 1 |  | 1 |  | 7 | Some concerns |
|  | GI symptoms cluster containing large GRV that should be greater than 200 mL | All-cause 60-day mortality | 1 |  | 1 |  | 1 |  | 1 | No mortality at the start of the study | 1 |  | 1 |  | 1 |  | 1 | 0.8 | 8 | Low |
|  |  | Length of ICU stay, Days | 1 |  | 1 |  | 1 |  | 0 |  | 1 |  | 1 |  | 1 |  | 1 |  | 7 | Some concerns |
|  |  | Length of hospital stay (survivors only), Days | 1 |  | 1 |  | 1 |  | 0 |  | 1 |  | 1 |  | 1 |  | 1 |  | 7 | Some concerns |
| Blaser et al, 2015 | Only large GRV >=500 mL | All-cause ICU mortality | 1 |  | 1 |  | 1 |  | 1 | No mortality at the start of the study | 1 |  | 1 | Reliable medical records | 1 | 90 | 1 | 1.0 | 8 | Low |
|  | >=250 mL |  | 1 |  | 1 |  | 1 |  | 1 |  | 1 |  | 1 |  | 1 |  | 1 |  | 8 | Low |
|  | >=1000 mL |  | 1 |  | 1 |  | 1 |  | 1 |  | 1 |  | 1 |  | 1 |  | 1 |  | 8 | Low |
|  | >=473 mL |  | 1 |  | 1 |  | 1 |  | 1 |  | 1 |  | 1 |  | 1 |  | 1 |  | 8 | Low |
|  | EF <20% of caloric needs on any feeding day |  | 1 |  | 1 |  | 1 |  | 1 |  | 1 |  | 1 |  | 1 |  | 1 |  | 8 | Low |
|  | <50% |  | 1 |  | 1 |  | 1 |  | 1 |  | 1 |  | 1 |  | 1 |  | 1 |  | 8 | Low |
|  | <23% |  | 1 |  | 1 |  | 1 |  | 1 |  | 1 |  | 1 |  | 1 |  | 1 |  | 8 | Low |
|  | EF <80% of caloric needs on feeding day 3 |  | 1 |  | 1 |  | 1 |  | 1 |  | 1 |  | 1 |  | 1 |  | 0 | 0.7 | 7 | Some concerns |
|  | <50% |  | 1 |  | 1 |  | 1 |  | 1 |  | 1 |  | 1 |  | 1 |  | 0 |  | 7 | Some concerns |
|  | <59% |  | 1 |  | 1 |  | 1 |  | 1 |  | 1 |  | 1 |  | 1 |  | 0 |  | 7 | Some concerns |
|  | EF <80% of caloric needs on feeding day 4 |  | 1 |  | 1 |  | 1 |  | 1 |  | 1 |  | 1 |  | 1 |  | 0 | 0.6 | 7 | Some concerns |
|  | <50% |  | 1 |  | 1 |  | 1 |  | 1 |  | 1 |  | 1 |  | 1 |  | 0 |  | 7 | Some concerns |
|  | <46% |  | 1 |  | 1 |  | 1 |  | 1 |  | 1 |  | 1 |  | 1 |  | 0 |  | 7 | Some concerns |
|  | At least 1 symptom among 4 candidates large-GRV-containing GI symptoms (1 out of 4) |  | 1 |  | 1 |  | 1 |  | 1 |  | 1 |  | 1 |  | 1 |  | 1 | 1.0 | 8 | Low |
|  | 2 out of 4 |  | 1 |  | 1 |  | 1 |  | 1 |  | 1 |  | 1 |  | 1 |  | 1 |  | 8 | Low |
|  | 3 out of 4 |  | 1 |  | 1 |  | 1 |  | 1 |  | 1 |  | 1 |  | 1 |  | 1 |  | 8 | Low |
|  | At least 1 symptom among 5 candidates large-GRV-containing GI symptoms (1 out of 5) |  | 1 |  | 1 |  | 1 |  | 1 |  | 1 |  | 1 |  | 1 |  | 1 |  | 8 | Low |
|  | 2 out of 5 |  | 1 |  | 1 |  | 1 |  | 1 |  | 1 |  | 1 |  | 1 |  | 1 |  | 8 | Low |
|  | 3 out of 5 |  | 1 |  | 1 |  | 1 |  | 1 |  | 1 |  | 1 |  | 1 |  | 1 |  | 8 | Low |
|  | Only large GRV >=500 mL | All-cause 90-day mortality | 1 |  | 1 |  | 1 |  | 1 |  | 1 |  | 1 |  | 1 |  | 1 |  | 8 | Low |
|  | >=250 mL |  | 1 |  | 1 |  | 1 |  | 1 |  | 1 |  | 1 |  | 1 |  | 1 |  | 8 | Low |
|  | >=1000 mL |  | 1 |  | 1 |  | 1 |  | 1 |  | 1 |  | 1 |  | 1 |  | 1 |  | 8 | Low |
|  | >=473 mL |  | 1 |  | 1 |  | 1 |  | 1 |  | 1 |  | 1 |  | 1 |  | 1 |  | 8 | Low |
|  | EF <20% of caloric needs on any feeding day |  | 1 |  | 1 |  | 1 |  | 1 |  | 1 |  | 1 |  | 1 |  | 1 |  | 8 | Low |
|  | <50% |  | 1 |  | 1 |  | 1 |  | 1 |  | 1 |  | 1 |  | 1 |  | 1 |  | 8 | Low |
|  | <23% |  | 1 |  | 1 |  | 1 |  | 1 |  | 1 |  | 1 |  | 1 |  | 1 |  | 8 | Low |
|  | <80% of caloric needs on feeding day 3 |  | 1 |  | 1 |  | 1 |  | 1 |  | 1 |  | 1 |  | 1 |  | 0 | 0.7 | 7 | Some concerns |
|  | <50% |  | 1 |  | 1 |  | 1 |  | 1 |  | 1 |  | 1 |  | 1 |  | 0 |  | 7 | Some concerns |
|  | <59% |  | 1 |  | 1 |  | 1 |  | 1 |  | 1 |  | 1 |  | 1 |  | 0 |  | 7 | Some concerns |
|  | EF <80% of caloric needs on feeding day 4 |  | 1 |  | 1 |  | 1 |  | 1 |  | 1 |  | 1 |  | 1 |  | 0 | 0.6 | 7 | Some concerns |
|  | <50% |  | 1 |  | 1 |  | 1 |  | 1 |  | 1 |  | 1 |  | 1 |  | 0 |  | 7 | Some concerns |
|  | <46% |  | 1 |  | 1 |  | 1 |  | 1 |  | 1 |  | 1 |  | 1 |  | 0 |  | 7 | Some concerns |
|  | At least 1 symptom among 4 candidates large-GRV-containing GI symptoms (1 out of 4) |  | 1 |  | 1 |  | 1 |  | 1 |  | 1 |  | 1 |  | 1 |  | 1 | 1.0 | 8 | Low |
|  | 2 out of 4 |  | 1 |  | 1 |  | 1 |  | 1 |  | 1 |  | 1 |  | 1 |  | 1 |  | 8 | Low |
|  | 3 out of 4 |  | 1 |  | 1 |  | 1 |  | 1 |  | 1 |  | 1 |  | 1 |  | 1 |  | 8 | Low |
|  | At least 1 symptom among 5 candidates large-GRV-containing GI symptoms (1 out of 5) |  | 1 |  | 1 |  | 1 |  | 1 |  | 1 |  | 1 |  | 1 |  | 1 |  | 8 | Low |
|  | 2 out of 5 |  | 1 |  | 1 |  | 1 |  | 1 |  | 1 |  | 1 |  | 1 |  | 1 |  | 8 | Low |
|  | 3 out of 5 |  | 1 |  | 1 |  | 1 |  | 1 |  | 1 |  | 1 |  | 1 |  | 1 |  | 8 | Low |
|  |  | All-cause ICU mortality (assessed by adjusted OR) | 1 |  | 1 |  | 1 |  | 1 |  | 2 | Adjusting for multiple demographic and medical covariates | 1 |  | 1 |  | 1 |  | 9 | Low |
|  | EF <23% of caloric needs on any feeding day | All-cause 90-day mortality (assessed by adjusted OR) | 1 |  | 1 |  | 1 |  | 1 |  | 2 |  | 1 |  | 1 |  | 1 |  | 9 | Low |
|  | Only large GRV >=500 mL |  | 1 |  | 1 |  | 1 |  | 1 |  | 2 |  | 1 |  | 1 |  | 1 |  | 9 | Low |
|  | >=250 mL |  | 1 |  | 1 |  | 1 |  | 1 |  | 2 |  | 1 |  | 1 |  | 1 |  | 9 | Low |
|  | >=1000 mL |  | 1 |  | 1 |  | 1 |  | 1 |  | 2 |  | 1 |  | 1 |  | 1 |  | 9 | Low |
|  | >=473 mL |  | 1 |  | 1 |  | 1 |  | 1 |  | 2 |  | 1 |  | 1 |  | 1 |  | 9 | Low |
|  | EF <20% of caloric needs on any feeding day |  | 1 |  | 1 |  | 1 |  | 1 |  | 2 |  | 1 |  | 1 |  | 1 |  | 9 | Low |
|  | <50% |  | 1 |  | 1 |  | 1 |  | 1 |  | 2 |  | 1 |  | 1 |  | 1 |  | 9 | Low |
|  | <23% |  | 1 |  | 1 |  | 1 |  | 1 |  | 2 |  | 1 |  | 1 |  | 1 |  | 9 | Low |
|  | EF <80% of caloric needs on feeding day 3 |  | 1 |  | 1 |  | 1 |  | 1 |  | 2 |  | 1 |  | 1 |  | 0 | 0.7 | 8 | Low |
|  | <50% |  | 1 |  | 1 |  | 1 |  | 1 |  | 2 |  | 1 |  | 1 |  | 0 |  | 8 | Low |
|  | <59% |  | 1 |  | 1 |  | 1 |  | 1 |  | 2 |  | 1 |  | 1 |  | 0 |  | 8 | Low |
|  | EF <80% of caloric needs on feeding day 4 |  | 1 |  | 1 |  | 1 |  | 1 |  | 2 |  | 1 |  | 1 |  | 0 | 0.6 | 8 | Low |
|  | <50% |  | 1 |  | 1 |  | 1 |  | 1 |  | 2 |  | 1 |  | 1 |  | 0 |  | 8 | Low |
|  | <46% |  | 1 |  | 1 |  | 1 |  | 1 |  | 2 |  | 1 |  | 1 |  | 0 |  | 8 | Low |
|  | At least 1 symptom among 4 candidates large-GRV-containing GI symptoms (1 out of 4) |  | 1 |  | 1 |  | 1 |  | 1 |  | 2 |  | 1 |  | 1 |  | 1 | 1.0 | 9 | Low |
|  | 2 out of 4 |  | 1 |  | 1 |  | 1 |  | 1 |  | 2 |  | 1 |  | 1 |  | 1 |  | 9 | Low |
|  | 3 out of 4 |  | 1 |  | 1 |  | 1 |  | 1 |  | 2 |  | 1 |  | 1 |  | 1 |  | 9 | Low |
|  | At least 1 symptom among 5 candidates large-GRV-containing GI symptoms (1 out of 5) |  | 1 |  | 1 |  | 1 |  | 1 |  | 2 |  | 1 |  | 1 |  | 1 |  | 9 | Low |
|  | 2 out of 5 |  | 1 |  | 1 |  | 1 |  | 1 |  | 2 |  | 1 |  | 1 |  | 1 |  | 9 | Low |
|  | 3 out of 5 |  | 1 |  | 1 |  | 1 |  | 1 |  | 2 |  | 1 |  | 1 |  | 1 |  | 9 | Low |
|  | Vomiting | All-cause ICU mortality | 1 |  | 1 |  | 1 |  | 1 |  | 1 | Possible control for important factors | 1 |  | 1 |  | 1 |  | 8 | Low |
|  | Absent bowel sounds |  | 1 |  | 1 |  | 1 |  | 1 |  | 1 |  | 1 |  | 1 |  | 1 |  | 8 | Low |
|  | Large GRV >=500 mL |  | 1 |  | 1 |  | 1 |  | 1 |  | 1 |  | 1 |  | 1 |  | 1 |  | 8 | Low |
|  | Abdominal distension |  | 1 |  | 1 |  | 1 |  | 1 |  | 1 |  | 1 |  | 1 |  | 1 |  | 8 | Low |
|  | Diarrhea |  | 1 |  | 1 |  | 1 |  | 1 |  | 1 |  | 1 |  | 1 |  | 1 |  | 8 | Low |
|  | Vomiting | All-cause 90-day mortality | 1 |  | 1 |  | 1 |  | 1 |  | 1 |  | 1 |  | 1 |  | 1 |  | 8 | Low |
|  | Absent bowel sounds |  | 1 |  | 1 |  | 1 |  | 1 |  | 1 |  | 1 |  | 1 |  | 1 |  | 8 | Low |
|  | Large GRV >500 mL |  | 1 |  | 1 |  | 1 |  | 1 |  | 1 |  | 1 |  | 1 |  | 1 |  | 8 | Low |
|  | Abdominal distension |  | 1 |  | 1 |  | 1 |  | 1 |  | 1 |  | 1 |  | 1 |  | 1 |  | 8 | Low |
|  | Diarrhea |  | 1 |  | 1 |  | 1 |  | 1 |  | 1 |  | 1 |  | 1 |  | 1 |  | 8 | Low |
| Merchan et al, 2017 | Large-GRV-containing GI symptoms cluster | All-cause hospital mortality | 0 | Select patients with specific diseases (septic shock) | 1 |  | 1 |  | 1 |  | 0 | No specific control for any factors | 1 |  | 1 | >=28 | 1 |  | 6 | Some concerns |
|  |  | All-cause ICU mortality | 0 |  | 1 |  | 1 |  | 1 |  | 0 |  | 1 |  | 1 |  | 1 |  | 6 | Some concerns |
|  |  | Length of hospital stay, Days | 0 |  | 1 |  | 1 |  | 0 | No clear description or uncertain | 0 |  | 1 |  | 1 |  | 1 |  | 5 | High |
|  |  | Length of ICU stay, Days | 0 |  | 1 |  | 1 |  | 0 |  | 0 |  | 1 |  | 1 |  | 1 |  | 5 | High |
| Wang&McIlroy et al, 2017 |  | Pneumonia | 1 | Somewhat representative of the average prevalence of critically ill patients | 1 |  | 1 |  | 0 |  | 0 |  | 1 |  | 1 | 30 | 1 |  | 6 | Some concerns |
|  |  | All-cause 30-day mortality | 1 |  | 1 |  | 1 |  | 1 | No mortality at the start of the study | 0 |  | 1 |  | 1 |  | 1 |  | 7 | Some concerns |
| Hu et al, 2017 | EF <80% of caloric needs in 7 feeding days | All-cause 28-day mortality | 1 |  | 1 |  | 1 |  | 1 |  | 1 | Possible control for important factors | 1 |  | 1 | 60 | 1 |  | 8 | Low |
|  |  | All-cause 60-day mortality | 1 |  | 1 |  | 1 |  | 1 |  | 1 |  | 1 |  | 1 |  | 1 |  | 8 | Low |
|  |  | Length of ICU stay, Days | 1 |  | 1 |  | 1 |  | 0 |  | 1 |  | 1 |  | 1 |  | 1 |  | 7 | Some concerns |
|  |  | All-cause 60-day mortality (assessed by adjusted OR) | 1 |  | 1 |  | 1 |  | 1 | No mortality at the start of the study | 2 | Adjusting for multiple demographic and medical covariates | 1 |  | 1 |  | 1 |  | 9 | Low |
|  |  |  | 1 |  | 1 |  | 1 |  | 1 |  | 2 |  | 1 |  | 1 |  | 1 |  | 9 | Low |
|  |  |  | 1 |  | 1 |  | 1 |  | 1 |  | 2 |  | 1 |  | 1 |  | 1 |  | 9 | Low |
| Li et al, 2019 | EF <80% of caloric needs in 3 feeding days | Length of hospital stay, Days | 0 | Select patients with specific diseases (moderately severe acute pancreatitis) | 1 |  | 1 |  | 0 | No clear description or uncertain | 0 | No specific control for any factors | 1 |  | 0 | >=20 | 1 |  | 4 | High |
| Virani et al, 2019 | EF <80% of caloric needs in 3 feeding days | All-cause mortality | 0 | Select patients with specific diseases (trauma) | 1 |  | 1 |  | 1 | No mortality at the start of the study | 0 |  | 1 |  | 1 | >=34 | 1 |  | 6 | Some concerns |
|  | Only large GRV >500 mL |  | 0 |  | 1 |  | 1 |  | 1 |  | 0 |  | 1 |  | 1 |  | 1 |  | 6 | Some concerns |
|  | Large-GRV-containing GI symptoms cluster |  | 0 |  | 1 |  | 1 |  | 1 |  | 0 |  | 1 |  | 1 |  | 1 |  | 6 | Some concerns |
|  | EF <80% of caloric needs in 3 feeding days | Length of hospital stay, Days | 0 |  | 1 |  | 1 |  | 0 | No clear description or uncertain | 0 |  | 1 |  | 1 |  | 1 |  | 5 | High |
|  |  | Length of ICU stay, Days | 0 |  | 1 |  | 1 |  | 0 |  | 0 |  | 1 |  | 1 |  | 1 |  | 5 | High |
|  | Only large GRV >500 mL | Length of hospital stay, Days | 0 |  | 1 |  | 1 |  | 0 |  | 0 |  | 1 |  | 1 |  | 1 |  | 5 | High |
|  |  | Length of ICU stay, Days | 0 |  | 1 |  | 1 |  | 0 |  | 0 |  | 1 |  | 1 |  | 1 |  | 5 | High |
|  | Large-GRV-containing GI symptoms cluster | Length of hospital stay, Days | 0 |  | 1 |  | 1 |  | 0 |  | 0 |  | 1 |  | 1 |  | 1 |  | 5 | High |
|  |  | Length of ICU stay, Days | 0 |  | 1 |  | 1 |  | 0 |  | 0 |  | 1 |  | 1 |  | 1 |  | 5 | High |
| Faramarzi et al, 2020 | Only large GRV >=250 mL | Pneumonia | 1 | Somewhat representative of the average prevalence of critically ill patients | 1 |  | 1 |  | 0 |  | 1 | Possible control for important factors | 1 |  | 0 | >=13 | 1 |  | 6 | Some concerns |
|  |  | All-cause mortality | 1 |  | 1 |  | 1 |  | 1 | No mortality at the start of the study | 1 |  | 1 |  | 0 |  | 1 |  | 7 | Some concerns |
|  |  | Mechanical ventilation days | 1 |  | 1 |  | 1 |  | 0 | No clear description or uncertain | 1 |  | 1 |  | 0 |  | 1 |  | 6 | Some concerns |
|  |  | Length of ICU stay, Days | 1 |  | 1 |  | 1 |  | 0 |  | 1 |  | 1 |  | 0 |  | 1 |  | 6 | Some concerns |
|  |  | All-cause mortality (assessed by adjusted OR) | 1 |  | 1 |  | 1 |  | 1 | No mortality at the start of the study | 2 | Adjusting for multiple demographic and medical covariates | 1 |  | 0 |  | 1 |  | 8 | Low |
|  |  | Pneumonia (assessed by adjusted OR) | 1 |  | 1 |  | 1 |  | 0 | No clear description or uncertain | 2 |  | 1 |  | 0 |  | 1 |  | 7 | Some concerns |
| Hu&Sun et al, 2020 | EF <80% of caloric needs in 3 feeding days | All-cause 28-day mortality | 1 |  | 1 |  | 1 |  | 1 | No mortality at the start of the study | 1 | Possible control for important factors | 1 |  | 1 | 60 | 1 |  | 8 | Low |
|  |  | All-cause 60-day mortality | 1 |  | 1 |  | 1 |  | 1 |  | 1 |  | 1 |  | 1 |  | 1 |  | 8 | Low |
|  |  | Length of ICU stay, Days | 1 |  | 1 |  | 1 |  | 0 | No clear description or uncertain | 1 |  | 1 |  | 1 |  | 1 |  | 7 | Some concerns |
|  |  | Mechanical ventilation days | 1 |  | 1 |  | 1 |  | 0 |  | 1 |  | 1 |  | 1 |  | 1 |  | 7 | Some concerns |
|  |  | All-cause 60-day mortality (assessed by adjusted OR) | 1 |  | 1 |  | 1 |  | 1 | No mortality at the start of the study | 2 | Adjusting for multiple demographic and medical covariates | 1 |  | 1 |  | 1 |  | 9 | Low |
| Mao et al, 2020 | Large-GRV-containing GI symptoms cluster | All-cause 28-day mortality | 0 | Select patients with specific diseases (consecutive sepsis) | 1 |  | 1 |  | 1 |  | 0 | No specific control for any factors | 1 |  | 0 | >=25 | 1 |  | 5 | High |
|  |  | Mechanical ventilation days | 0 |  | 1 |  | 1 |  | 0 | No clear description or uncertain | 0 |  | 1 |  | 0 |  | 1 |  | 4 | High |
|  |  | Length of ICU stay, Days | 0 |  | 1 |  | 1 |  | 0 |  | 0 |  | 1 |  | 0 |  | 1 |  | 4 | High |
| Sierp et al, 2020 | Only large GRV >=250 mL | All-cause ICU mortality | 0 | Select patients with specific diseases (burns) | 1 |  | 1 |  | 1 | No mortality at the start of the study | 0 |  | 1 |  | 1 | >=94 | 1 |  | 6 | Some concerns |
|  |  | All-cause hospital mortality | 0 |  | 1 |  | 1 |  | 1 |  | 0 |  | 1 |  | 1 |  | 1 |  | 6 | Some concerns |
|  |  | Pneumonia | 0 |  | 1 |  | 1 |  | 0 | No clear description or uncertain | 0 |  | 1 |  | 1 |  | 1 |  | 5 | High |
|  |  | Mechanical ventilation days | 0 |  | 1 |  | 1 |  | 0 |  | 0 |  | 1 |  | 1 |  | 1 |  | 5 | High |
| Heyland et al, 2021 | Large-GRV-containing GI symptoms cluster | All-cause 60-day mortality | 1 | Somewhat representative of the average prevalence of critically ill patients | 1 |  | 1 |  | 1 | No mortality at the start of the study | 1 | Possible control for important factors | 1 | Record linkage | 1 | 60 | 1 |  | 8 | Low |
|  |  | Length of ICU stay, Days | 1 |  | 1 |  | 1 |  | 0 |  | 1 |  | 1 |  | 1 |  | 1 |  | 7 | Some concerns |
|  |  | Length of ICU stay (among 60-day hospital survivors), Days | 1 |  | 1 |  | 1 |  | 0 |  | 1 |  | 1 |  | 1 |  | 1 | 0.8 | 7 | Some concerns |
|  |  | Length of hospital stay (among 60-day hospital survivors), Days | 1 |  | 1 |  | 1 |  | 0 |  | 1 |  | 1 |  | 1 |  | 1 | 1.0 | 7 | Some concerns |
|  |  | All-cause 60-day mortality (assessed by adjusted OR) | 1 |  | 1 |  | 1 |  | 1 | No mortality at the start of the study | 2 | Adjusting for multiple demographic and medical covariates | 1 |  | 1 |  | 1 |  | 9 | Low |
|  |  | Length of ICU stay (assessed by adjusted OR) | 1 |  | 1 |  | 1 |  | 0 | No clear description or uncertain | 2 |  | 1 |  | 1 |  | 1 |  | 8 | Low |
|  |  | Length of ICU stay (among 60-day hospital survivors) (assessed by adjusted OR) | 1 |  | 1 |  | 1 |  | 0 |  | 2 |  | 1 |  | 1 |  | 1 | 0.8 | 8 | Low |
|  |  | All-cause 60-day mortality | 1 |  | 1 |  | 1 |  | 1 | No mortality at the start of the study | 1 | Possible control for important factors | 1 |  | 1 |  | 0 | 0.2 | 7 | Some concerns |
|  |  | Length of ICU stay, Days | 1 |  | 1 |  | 1 |  | 0 |  | 1 |  | 1 |  | 1 |  | 0 |  | 6 | Some concerns |
|  |  | Length of ICU stay (among 60-day hospital survivors), Days | 1 |  | 1 |  | 1 |  | 0 |  | 1 |  | 1 |  | 1 |  | 0 | 0.2 | 6 | Some concerns |
|  |  | All-cause 60-day mortality (assessed by adjusted OR) | 1 |  | 1 |  | 1 |  | 1 | No mortality at the start of the study | 2 | Adjusting for multiple demographic and medical covariates | 1 |  | 1 |  | 0 | 0.2 | 8 | Low |
|  |  | Length of ICU stay (assessed by adjusted OR) | 1 |  | 1 |  | 1 |  | 0 | No clear description or uncertain | 2 |  | 1 |  | 1 |  | 0 |  | 7 | Some concerns |
|  |  | Length of ICU stay (among 60-day hospital survivors) (assessed by adjusted OR) | 1 |  | 1 |  | 1 |  | 0 |  | 2 |  | 1 |  | 1 |  | 0 | 0.2 | 7 | Some concerns |
| Lin&Liu et al, 2021 | Vomiting | All-cause 28-day mortality | 1 |  | 1 |  | 1 |  | 1 | No mortality at the start of the study | 0 | No specific control for any factors | 1 |  | 1 | 28 | 1 | 1.0 | 7 | Some concerns |
|  | Absent bowel sounds |  | 1 |  | 1 |  | 1 |  | 1 |  | 0 |  | 1 |  | 1 |  | 1 |  | 7 | Some concerns |
|  | Only large GRV ˃500 mL |  | 1 |  | 1 |  | 1 |  | 1 |  | 0 |  | 1 |  | 1 |  | 1 |  | 7 | Some concerns |
|  | Abdominal distension |  | 1 |  | 1 |  | 1 |  | 1 |  | 0 |  | 1 |  | 1 |  | 1 |  | 7 | Some concerns |
|  | Diarrhea |  | 1 |  | 1 |  | 1 |  | 1 |  | 0 |  | 1 |  | 1 |  | 1 |  | 7 | Some concerns |
|  | At least 1 symptom among 4 candidates large-GRV-containing GI symptoms (1 out of 4) |  | 1 |  | 1 |  | 1 |  | 1 |  | 0 |  | 1 |  | 1 |  | 1 |  | 7 | Some concerns |
|  | 2 out of 4 |  | 1 |  | 1 |  | 1 |  | 1 |  | 0 |  | 1 |  | 1 |  | 1 |  | 7 | Some concerns |
|  | 3 out of 4 |  | 1 |  | 1 |  | 1 |  | 1 |  | 0 |  | 1 |  | 1 |  | 1 |  | 7 | Some concerns |
|  | At least 1 symptom among 5 candidates large-GRV-containing GI symptoms (1 out of 5) |  | 1 |  | 1 |  | 1 |  | 1 |  | 0 |  | 1 |  | 1 |  | 1 |  | 7 | Some concerns |
|  | 2 out of 5 |  | 1 |  | 1 |  | 1 |  | 1 |  | 0 |  | 1 |  | 1 |  | 1 |  | 7 | Some concerns |
|  | 3 out of 5 |  | 1 |  | 1 |  | 1 |  | 1 |  | 0 |  | 1 |  | 1 |  | 1 |  | 7 | Some concerns |
| Lin&Chen et al, 2021 | Large-GRV-containing GI symptoms cluster | All-cause mortality | 0 | Select patients with specific diseases (cardiopulmonary bypass) | 1 |  | 1 |  | 1 |  | 1 | Possible control for important factors | 1 | Reliable medical records | 0 | >=26 | 1 |  | 6 | Some concerns |
|  |  | Length of hospital stay, Days | 0 |  | 1 |  | 1 |  | 0 | No clear description or uncertain | 1 |  | 1 |  | 0 |  | 1 |  | 5 | High |
| Liu et al, 2021 | GI symptoms cluster without large GRV | All-cause mortality | 0 | Select patients with specific diseases (COVID-19) | 1 |  | 1 |  | 1 | No mortality at the start of the study | 1 |  | 1 |  | 1 | >=42 | 1 |  | 7 | Some concerns |
|  |  | Length of ICU stay, Days | 0 |  | 1 |  | 1 |  | 0 | No clear description or uncertain | 1 |  | 1 |  | 1 |  | 1 |  | 6 | Some concerns |
|  |  | Length of hospital stay, Days | 0 |  | 1 |  | 1 |  | 0 |  | 1 |  | 1 |  | 1 |  | 1 |  | 6 | Some concerns |
|  |  | Mechanical ventilation days | 0 |  | 1 |  | 1 |  | 0 |  | 1 |  | 1 |  | 1 |  | 1 |  | 6 | Some concerns |
|  |  | All-cause mortality (assessed by adjusted OR) | 0 |  | 1 |  | 1 |  | 1 | No mortality at the start of the study | 2 | Adjusting for multiple demographic and medical covariates | 1 |  | 1 |  | 1 |  | 8 | Low |
| Yahyapoor et al, 2021 | Large-GRV-containing GI symptoms cluster | Length of hospital stay, Days | 1 | Somewhat representative of the average prevalence of critically ill patients | 1 |  | 1 |  | 0 | No clear description or uncertain | 0 | No specific control for any factors | 1 |  | 0 | >=20 | 1 |  | 5 | High |
|  |  | Length of ICU stay, Days | 1 |  | 1 |  | 1 |  | 0 |  | 0 |  | 1 |  | 0 |  | 1 |  | 5 | High |
| Drakos et al, 2022 | EF <80% of caloric needs in 3 feeding days | All-cause 50-day mortality | 0 | Select patients with specific diseases (COVID-19) | 1 |  | 1 |  | 1 | No mortality at the start of the study | 0 |  | 1 |  | 1 | >=150 | 1 | 1.0 | 6 | Some concerns |
|  |  | All-cause 100-day mortality | 0 |  | 1 |  | 1 |  | 1 |  | 0 |  | 1 |  | 1 |  | 1 |  | 6 | Some concerns |
|  |  | All-cause 150-day mortality | 0 |  | 1 |  | 1 |  | 1 |  | 0 |  | 1 |  | 1 |  | 1 |  | 6 | Some concerns |
| Hu&Deng et al, 2022 | Large-GRV-containing GI symptoms cluster | All-cause 28-day mortality | 0 | Select patients with specific diseases (sepsis) | 1 |  | 1 |  | 1 |  | 0 |  | 1 |  | 1 | 28 | 1 | 1.0 | 6 | Some concerns |
| Wang&Yang et al, 2022 | GI symptoms cluster without large GRV |  | 1 | Somewhat representative of the average prevalence of critically ill patients | 1 |  | 1 |  | 1 |  | 0 |  | 1 |  | 1 |  | 1 |  | 7 | Some concerns |

OR=odds ratio, GRV=gastric residual volume, EF=enteral feeding, GI=gastrointestinal, ICU=intensive care unit, APACHE II=Acute Physiology and Chronic Health Evaluation II.
