# Supplementary material for: Different definitions of feeding intolerance and their associations with outcomes of critically ill adults receiving enteral nutrition: a systematic review and meta-analysis
Source: J Intensive Care. 2023 Jul 5;11:29. doi: 10.1186/s40560-023-00674-3 (PMC10320932; doi:10.1186/s40560-023-00674-3)
Supplement: Supplementary file 8 — Additional file 8. Table S6: Characteristics of included studies. [file 40560_2023_674_MOESM8_ESM.docx]

# Table S6: Characteristics of included studies

| **Author, year** | **Study design (location of the study)** | **Patients** | | | **Exposure** | | **Outcome** | | |
| --- | --- | --- | --- | --- | --- | --- | --- | --- | --- |
|  |  | **sample size (male% )** | **Age** | **Diseases** | **Number of patients** | **Definition of the exposed cohort** | **Outcome indicators** | **Absolute effect (n.event/total or mean±sd for exposed cohort)** | **Relative effect (adjusted** **OR, 95%** **CI)** |
| Mentec et al, 2001 | Prospective (France) | 153 (56) | 65±15 | Surgery%: 26, Trauma%: 5, Digestive%: 7 | 70 | Large-GRV-containing GI symptoms cluster | Pneumonia | 30/70 | - |
|  |  |  |  |  |  |  | All-cause ICU mortality | 29/70 | - |
|  |  |  |  |  |  |  | All-cause hospital mortality | 37/70 | - |
|  |  |  |  |  |  |  | Length of ICU stay, days | 23±21 | - |
| Brown et al, 2003 | Prospective (America) | 20 (70) | 34.7±12.9 | Surgery%: 0, Trauma%: 100, Mechanical ventilation%: 100 | 6 |  | Mechanical ventilation days | 12.1±13.5 | - |
|  |  |  |  |  |  |  | Length of ICU stay, days | 14.5±6.9 | - |
|  |  |  |  |  |  |  | Length of hospital stay, days | 30.7±15.2 | - |
| Nguyen&Ching et al, 2007 | Retrospective (Australia) | 145 (62) | 54.3±2.3 | Surgery%: 18, Trauma%: 58, Burns%: 10, Sepsis%: 54, Mechanical ventilation%: 100 | 95 |  | Length of ICU stay, days | 18±2 | - |
| Nguyen&Lam et al, 2007 |  | 272 (54) | 54.8±18.4 | Surgery%: 31, Trauma%: 22, Burns%: 5 | 155 |  |  | 19.3±34.6 | - |
| Stevens et al, 2008 | Retrospective (America) | 61 (75) | 33.8±16.3 | Surgery%: 0 | 29 | Only large GRV >75 mL×2 times | All-cause mortality | 6/29 | - |
|  |  |  |  |  |  |  | Mechanical ventilation days | 19.1±11.1 | - |
|  |  |  |  |  |  |  | Length of ICU stay, days | 25.8±12 | - |
|  |  |  |  |  |  |  | Length of hospital stay, days | 42.6±36.4 | - |
| Bejarano et al, 2013 | Prospective (Spain) | 72 (67) | 57.7±14.7 | Surgery%: 22, Trauma%: 42, Digestive%: 9, Sepsis%: 24, Mechanical ventilation%: 95 | 21 | EF insufficiency in 3 feeding days | All-cause hospital mortality | 9/21 | - |
| Lavrentieva et al, 2014 | Prospective (Greece) | 46 (76) | 49.7±21.7 | Surgery%: 0, Burns%: 100, Sepsis%: 100 | 16 | Large-GRV-containing GI symptoms cluster | All-cause ICU mortality | 10/16 | - |
|  |  |  |  |  |  |  | Mechanical ventilation days (survivors only) | 25.5±10.4 | - |
|  |  |  |  |  |  |  | Length of ICU stay (survivors only), days | 31±23 | - |
| Gungabissoon et al, 2015 | Retrospective (21 countries, 167 ICUs) | 1888 (61) | 57.6±17.6 | Surgery%: 9, Trauma%: 14, Digestive%: 3, Sepsis%: 12, Mechanical ventilation%: 100 | 576 |  | All-cause 60-day mortality | 177/576 | - |
|  |  |  |  |  |  |  | Length of ICU stay, days | 19.1±16.1 | - |
|  |  |  |  |  |  |  | Length of hospital stay (survivors only), days | 26.8±15.5 | - |
|  |  |  |  | Abdominal surgery%: 5, Trauma%: 14, Digestive%: 3, Sepsis%: 12, Mechanical ventilation%: 100 | 451 | GI symptoms cluster containing large GRV that should be greater than 200 mL | All-cause 60-day mortality | 133/451 | - |
|  |  |  |  |  |  |  | Length of ICU stay, days | 19.1±15.9 | - |
|  |  |  |  |  |  |  | Length of hospital stay (survivors only), days | 28.5±18.9 | - |
| Blaser et al, 2015 | Prospective (Estonia) | 1712 (63) | 71.5±65.2 | Mechanical ventilation%: 82 | 371 | Only large GRV >=500 mL | All-cause ICU mortality | 85/371 | - |
|  |  |  |  |  | 690 | >=250 mL |  | 128/690 | - |
|  |  |  |  |  | 128 | >=1000 mL |  | 37/128 | - |
|  |  |  |  |  | 381 | >=473 mL |  | 86/381 | - |
|  |  |  |  |  | 928 | EF <20% of caloric needs on any feeding day |  | 167/928 | - |
|  |  |  |  |  | 1459 | <50% |  | 213/1459 | - |
|  |  |  |  |  | 989 | <23% |  | 181/989 | - |
|  |  |  |  |  | 897 | EF <80% of caloric needs on feeding day 3 |  | 113/897 | - |
|  |  |  |  |  | 644 | <50% |  | 91/644 | - |
|  |  |  |  |  | 574 | <59% |  | 86/574 | - |
|  |  |  |  |  | 728 | EF <80% of caloric needs on feeding day 4 |  | 96/728 | - |
|  |  |  |  |  | 478 | <50% |  | 79/478 | - |
|  |  |  |  |  | 447 | <46% |  | 78/447 | - |
|  |  |  |  |  | 848 | At least 1 symptom among 4 candidates large-GRV-containing GI symptoms (1 out of 4) |  | 150/848 | - |
|  |  |  |  |  | 391 | 2 out of 4 |  | 87/391 | - |
|  |  |  |  |  | 79 | 3 out of 4 |  | 24/79 | - |
|  |  |  |  |  | 972 | At least 1 symptom among 5 candidates large-GRV-containing GI symptoms (1 out of 5) |  | 163/972 | - |
|  |  |  |  |  | 520 | 2 out of 5 |  | 112/520 | - |
|  |  |  |  |  | 146 | 3 out of 5 |  | 52/146 | - |
|  |  |  |  |  | 371 | Only large GRV >=500 mL | All-cause 90-day mortality | 138/371 | - |
|  |  |  |  |  | 690 | >=250 mL |  | 237/690 | - |
|  |  |  |  |  | 128 | >=1000 mL |  | 55/128 | - |
|  |  |  |  |  | 381 | >=473 mL |  | 140/381 | - |
|  |  |  |  |  | 928 | EF <20% of caloric needs on any feeding day |  | 345/928 | - |
|  |  |  |  |  | 1459 | <50% |  | 456/1459 | - |
|  |  |  |  |  | 989 | <23% |  | 372/989 | - |
|  |  |  |  |  | 897 | <80% of caloric needs on feeding day 3 |  | 274/897 | - |
|  |  |  |  |  | 644 | <50% |  | 210/644 | - |
|  |  |  |  |  | 574 | <59% |  | 188/574 | - |
|  |  |  |  |  | 728 | EF <80% of caloric needs on feeding day 4 |  | 231/728 | - |
|  |  |  |  |  | 478 | <50% |  | 161/478 | - |
|  |  |  |  |  | 447 | <46% |  | 157/447 | - |
|  |  |  |  |  | 848 | At least 1 symptom among 4 candidates large-GRV-containing GI symptoms (1 out of 4) |  | 298/848 | - |
|  |  |  |  |  | 391 | 2 out of 4 |  | 147/391 | - |
|  |  |  |  |  | 79 | 3 out of 4 |  | 37/79 | - |
|  |  |  |  |  | 972 | At least 1 symptom among 5 candidates large-GRV-containing GI symptoms (1 out of 5) |  | 327/972 | - |
|  |  |  |  |  | 520 | 2 out of 5 |  | 197/520 | - |
|  |  |  |  |  | 146 | 3 out of 5 |  | 74/146 | - |
|  |  |  |  |  |  |  | All-cause ICU mortality | - | 3.39 (2.23 to 5.14) |
|  |  |  |  |  | 989 | EF <23% of caloric needs on any feeding day | All-cause 90-day mortality | - | 2.34 (1.8 to 3.04)* |
|  |  |  |  |  | 371 | Only large GRV >=500 mL |  | - | 1.07 (0.81 to 1.41) |
|  |  |  |  |  | 690 | >=250 mL |  | - | 0.98 (0.77 to 1.25) |
|  |  |  |  |  | 128 | >=1000 mL |  | - | 1.39 (0.29 to 2.11) |
|  |  |  |  |  | 381 | >=473 mL |  | - | 1.03 (0.78 to 1.36) |
|  |  |  |  |  | 928 | EF <20% of caloric needs on any feeding day |  | - | 1.71 (1.33 to 2.19) |
|  |  |  |  |  | 1459 | <50% |  | - | 1.24 (0.83 to 1.85) |
|  |  |  |  |  | 989 | <23% |  | - | 2.07 (1.59 to 2.7)* |
|  |  |  |  |  | 897 | EF <80% of caloric needs on feeding day 3 |  | - | 0.97 (0.74 to 1.27) |
|  |  |  |  |  | 644 | <50% |  | - | 1.2 (0.88 to 1.63) |
|  |  |  |  |  | 574 | <59% |  | - | 1.02 (0.81 to 1.3) |
|  |  |  |  |  | 728 | EF <80% of caloric needs on feeding day 4 |  | - | 0.91 (0.69 to 1.19) |
|  |  |  |  |  | 478 | <50% |  | - | 0.95 (0.77 to 1.32) |
|  |  |  |  |  | 447 | <46% |  | - | 1.04 (0.8 to 1.35) |
|  |  |  |  |  | 848 | At least 1 symptom among 4 candidates large-GRV-containing GI symptoms (1 out of 4) |  | - | 1.13 (0.88 to 1.44) |
|  |  |  |  |  | 391 | 2 out of 4 |  | - | 1.13 (0.86 to 1.49) |
|  |  |  |  |  | 79 | 3 out of 4 |  | - | 1.32 (0.77 to 2.25) |
|  |  |  |  |  | 972 | At least 1 symptom among 5 candidates large-GRV-containing GI symptoms (1 out of 5) |  | - | 1.07 (0.83 to 1.38) |
|  |  |  |  |  | 520 | 2 out of 5 |  | - | 1.25 (0.97 to 1.61) |
|  |  |  |  |  | 146 | 3 out of 5 |  | - | 1.64 (1.1 to 2.44) |
|  |  |  |  |  | 432 | Vomiting | All-cause ICU mortality | 62/432 | - |
|  |  |  |  |  | 370 | Absent bowel sounds |  | 81/370 | - |
|  |  |  |  |  | 176 | Large GRV >=500 mL |  | 39/176 | - |
|  |  |  |  |  | 67 | Abdominal distension |  | 25/67 | - |
|  |  |  |  |  | 56 | Diarrhea |  | 17/56 | - |
|  |  |  |  |  | 432 | Vomiting | All-cause 90-day mortality | 120/432 | - |
|  |  |  |  |  | 370 | Absent bowel sounds |  | 137/370 | - |
|  |  |  |  |  | 176 | Large GRV >500 mL |  | 66/176 | - |
|  |  |  |  |  | 67 | Abdominal distension |  | 37/67 | - |
|  |  |  |  |  | 56 | Diarrhea |  | 31/56 | - |
| Merchan et al, 2017 | Retrospective (America) | 120 (60) | 65.5±19.4 | Surgery%: 0, Sepsis%: 100, Mechanical ventilation%: 100 | 46 | Large-GRV-containing GI symptoms cluster | All-cause hospital mortality | 22/46 | - |
|  |  |  |  |  |  |  | All-cause ICU mortality | 20/46 | - |
|  |  |  |  |  |  |  | Length of hospital stay, days | 20±11.8 | - |
|  |  |  |  |  |  |  | Length of ICU stay, days | 13.3±8.1 | - |
| Wang&McIlroy et al, 2017 | Retrospective (New Zealand) | 455 (62) | 56±50 | Surgery%: 70, Digestive%: 2, Sepsis%: 7 | 162 |  | Pneumonia | 2/162 | - |
|  |  |  |  |  |  |  | All-cause 30-day mortality | 32/162 | - |
| Hu et al, 2017 | Prospective (China, 14 ICUs) | 418 (72) | 65.1±18.3 | Surgery%: 17, Mechanical ventilation%: 85 | 106 | EF <80% of caloric needs in 7 feeding days | All-cause 28-day mortality | 41/106 | - |
|  |  |  |  |  |  |  | All-cause 60-day mortality | 47/106 | - |
|  |  |  |  |  |  |  | Length of ICU stay, days | 12.6±5.52 | - |
|  |  |  |  |  |  |  | All-cause 60-day mortality | - | 2.55 (1.79 to 3.65)* |
|  |  |  |  |  |  |  |  | - | 2.33 (1.59 to 3.4)* |
|  |  |  |  |  |  |  |  | - | 2.41 (1.65 to 3.51)* |
| Li et al, 2019 | Retrospective (China) | 568 (58) | 48.7±42.3 | Surgery%: 0, Digestive%: 100 | 184 | EF <80% of caloric needs in 3 feeding days | Length of hospital stay, days | 15.8±12.7 | - |
| Virani et al, 2019 | Retrospective (America) | 147 (73) | 42.2±3.0 | Surgery%: 0, Trauma%: 100 | 49 | EF <80% of caloric needs in 3 feeding days | All-cause mortality | 4/49 | - |
|  |  |  |  |  | 67 | Only large GRV >500 mL |  | 7/67 | - |
|  |  |  |  |  | 59 | Large-GRV-containing GI symptoms cluster |  | 4/59 | - |
|  |  |  |  |  | 49 | EF <80% of caloric needs in 3 feeding days | Length of hospital stay, days | 29.6±2.3 | - |
|  |  |  |  |  |  |  | Length of ICU stay, days | 16.7±1.5 | - |
|  |  |  |  |  | 67 | Only large GRV >500 mL | Length of hospital stay, days | 30±2.2 | - |
|  |  |  |  |  |  |  | Length of ICU stay, days | 15.5±1.3 | - |
|  |  |  |  |  | 59 | Large-GRV-containing GI symptoms cluster | Length of hospital stay, days | 33.9±2.6 | - |
|  |  |  |  |  |  |  | Length of ICU stay, days | 17.2±3.6 | - |
| Faramarzi et al, 2020 | Prospective (Iran) | 150 (63) | 57.7±19.0 | Surgery%: 0, Trauma%: 16, Sepsis%: 9, Mechanical ventilation%: 100 | 53 | Only large GRV >=250 mL | Pneumonia | 15/53 | - |
|  |  |  |  |  |  |  | All-cause mortality | 19/53 | - |
|  |  |  |  |  |  |  | Mechanical ventilation days | 8.71±4.33 | - |
|  |  |  |  |  |  |  | Length of ICU stay, days | 12.96±5.86 | - |
|  |  |  |  |  |  |  | All-cause mortality | - | 1.96 (0.73 to 5.28) |
|  |  |  |  |  |  |  | Pneumonia | - | 0.78 (0.27 to 2.18) |
| Hu&Sun et al, 2020 | Prospective (China, 14 ICUs) | 499 (68) | 65.0±18.1 | Surgery%: 19, Sepsis%: 34 | 196 | EF <80% of caloric needs in 3 feeding days | All-cause 28-day mortality | 70/196 | - |
|  |  |  |  |  |  |  | All-cause 60-day mortality | 79/196 | - |
|  |  |  |  |  |  |  | Length of ICU stay, days | 11.1±6.1 | - |
|  |  |  |  |  |  |  | Mechanical ventilation days | 9.96±6.23 | - |
|  |  |  |  |  |  |  | All-cause 60-day mortality | - | 1.66 (1.22 to 2.26) |
| Mao et al, 2020 | Retrospective (China) | 132 (70) | 60.6±18.1 | Surgery%: 0, Sepsis%: 100 | 35 | Large-GRV-containing GI symptoms cluster | All-cause 28-day mortality | 11/35 | - |
|  |  |  |  |  |  |  | Mechanical ventilation days | 17.5±13.5 | - |
|  |  |  |  |  |  |  | Length of ICU stay, days | 23.7±14.7 | - |
| Sierp et al, 2020 | Retrospective (Australia) | 59 (83) | 43.0±18.5 | Surgery%: 0, Burns%: 100, Mechanical ventilation%: 100 | 30 | Only large GRV >=250 mL | All-cause ICU mortality | 5/30 | - |
|  |  |  |  |  |  |  | All-cause hospital mortality | 0/30 | - |
|  |  |  |  |  |  |  | Pneumonia | 8/30 | - |
|  |  |  |  |  |  |  | Mechanical ventilation days | 8±7.4 | - |
| Heyland et al, 2021 | Retrospective (41 countries, 785 ICUs) | 15918 (61) | 59.4±17.6 | Surgery%: 32, Mechanical ventilation%: 100 | 4036 | Large-GRV-containing GI symptoms cluster | All-cause 60-day mortality | 1251/4036 | - |
|  |  |  |  |  |  |  | Length of ICU stay, days | 20.3±19.2 | - |
|  |  |  |  | Mechanical ventilation%: 82 | 2785 |  | Length of ICU stay (among 60-day hospital survivors), days | 22.3±21.9 | - |
|  |  |  |  | Surgery%: 32, Mechanical ventilation%: 100 | 4036 |  | Length of hospital stay (among 60-day hospital survivors), days | 47.8±27.7 | - |
|  |  |  |  |  |  |  | All-cause 60-day mortality | - | 1.5 (1.38 to 1.64) |
|  |  |  |  |  |  |  | Length of ICU stay, days | - | 0.74 (0.71 to 0.77) |
|  |  |  |  | Mechanical ventilation%: 82 | 2785 |  | Length of ICU stay (among 60-day hospital survivors), days | - | 0.7 (0.66 to 0.73) |
|  |  |  |  |  | 633 |  | All-cause 60-day mortality | 194/633 | - |
|  |  |  |  |  |  |  | Length of ICU stay, days | 19.5±15.6 | - |
|  |  |  |  |  | 439 |  | Length of ICU stay (among 60-day hospital survivors), days | 21.5±16.9 | - |
|  |  |  |  |  | 633 |  | All-cause 60-day mortality | - | 1.44 (1.16 to 1.77) |
|  |  |  |  |  |  |  | Length of ICU stay, days | - | 0.73 (0.67 to 0.8) |
|  |  |  |  |  | 439 |  | Length of ICU stay (among 60-day hospital survivors), days | - | 0.65 (0.58 to 0.73) |
| Lin&Liu et al, 2021 | Retrospective (China, 118 ICUs) | 1098 (71) | 67.7±19.5 | Sepsis%: 2, Mechanical ventilation%: 26 | 62 | Vomiting | All-cause 28-day mortality | 19/62 | - |
|  |  |  |  |  | 38 | Absent bowel sounds |  | 6/38 | - |
|  |  |  |  |  | 3 | Only large GRV ˃500 mL |  | 1/3 | - |
|  |  |  |  |  | 317 | Abdominal distension |  | 82/317 | - |
|  |  |  |  |  | 103 | Diarrhea |  | 34/103 | - |
|  |  |  |  |  | 390 | At least 1 symptom among 4 candidates large-GRV-containing GI symptoms (1 out of 4) |  | 102/390 | - |
|  |  |  |  |  | 89 | 2 out of 4 |  | 32/89 | - |
|  |  |  |  |  | 6 | 3 out of 4 |  | 2/6 | - |
|  |  |  |  |  | 413 | At least 1 symptom among 5 candidates large-GRV-containing GI symptoms (1 out of 5) |  | 106/413 | - |
|  |  |  |  |  | 103 | 2 out of 5 |  | 33/103 | - |
|  |  |  |  |  | 7 | 3 out of 5 |  | 3/7 | - |
| Lin&Chen et al, 2021 | Prospective (China) | 237 (59) | 53.8±12.3 | Surgery%: 100 | 152 | Large-GRV-containing GI symptoms cluster | All-cause mortality | 6/152 | - |
|  |  |  |  |  |  |  | Length of hospital stay, days | 21.1±8 | - |
| Liu et al, 2021 | Retrospective (America) | 323 (64) | 59.6±14.9 | Surgery%: 0 | 180 | GI symptoms cluster without large GRV | All-cause mortality | 61/180 | - |
|  |  |  |  |  |  |  | Length of ICU stay, days | 22.7±11.5 | - |
|  |  |  |  |  |  |  | Length of hospital stay, days | 30.5±17 | - |
|  |  |  |  |  |  |  | Mechanical ventilation days | 22.4±13.3 | - |
|  |  |  |  |  |  |  | All-cause mortality | - | 5.95 (3.53 to 10.03) |
| Yahyapoor et al, 2021 | Prospective (Iran, 3 ICUs) | 245 (50) | 58.4±19.2 | Surgery%: 0, Digestive%: 10, Sepsis%: 23 | 162 | Large-GRV-containing GI symptoms cluster | Length of hospital stay, days | 20.2±21.7 | - |
|  |  |  |  |  |  |  | Length of ICU stay, days | 17.38±17.5 | - |
| Drakos et al, 2022 | Retrospective (America) | 218 (69) | 59.8±14.2 | Surgery%: 0 | 138 | EF <80% of caloric needs in 3 feeding days | All-cause 50-day mortality | 70/138 | - |
|  |  |  |  |  |  |  | All-cause 100-day mortality | 74/138 | - |
|  |  |  |  |  |  |  | All-cause 150-day mortality | 76/138 | - |
| Hu&Deng et al, 2022 | Retrospective (China, 2 ICUs) | 195 (71) | 71.5±13.8 | Surgery%: 0, Sepsis%: 100, Mechanical ventilation%: 76 | 86 | Large-GRV-containing GI symptoms cluster | All-cause 28-day mortality | 6/86 | - |
| Wang&Yang et al, 2022 | Retrospective (China) | 38 (63) | 61±14.4 | Surgery%: 0, Trauma%: 11, Sepsis%: 29 | 17 | GI symptoms cluster without large GRV |  | 2/17 | - |

OR=odds ratio, CI= confidence interval, GRV=Gastric residual volume, GI=gastrointestinal, EF=enteral feeding, ICU=intensive care unit.
