# Supplementary material for: Different definitions of feeding intolerance and their associations with outcomes of critically ill adults receiving enteral nutrition: a systematic review and meta-analysis
Source: J Intensive Care. 2023 Jul 5;11:29. doi: 10.1186/s40560-023-00674-3 (PMC10320932; doi:10.1186/s40560-023-00674-3)

# Fig S3: Forest plots of partial main results

## 3.1 Association between FI and all-cause mortality


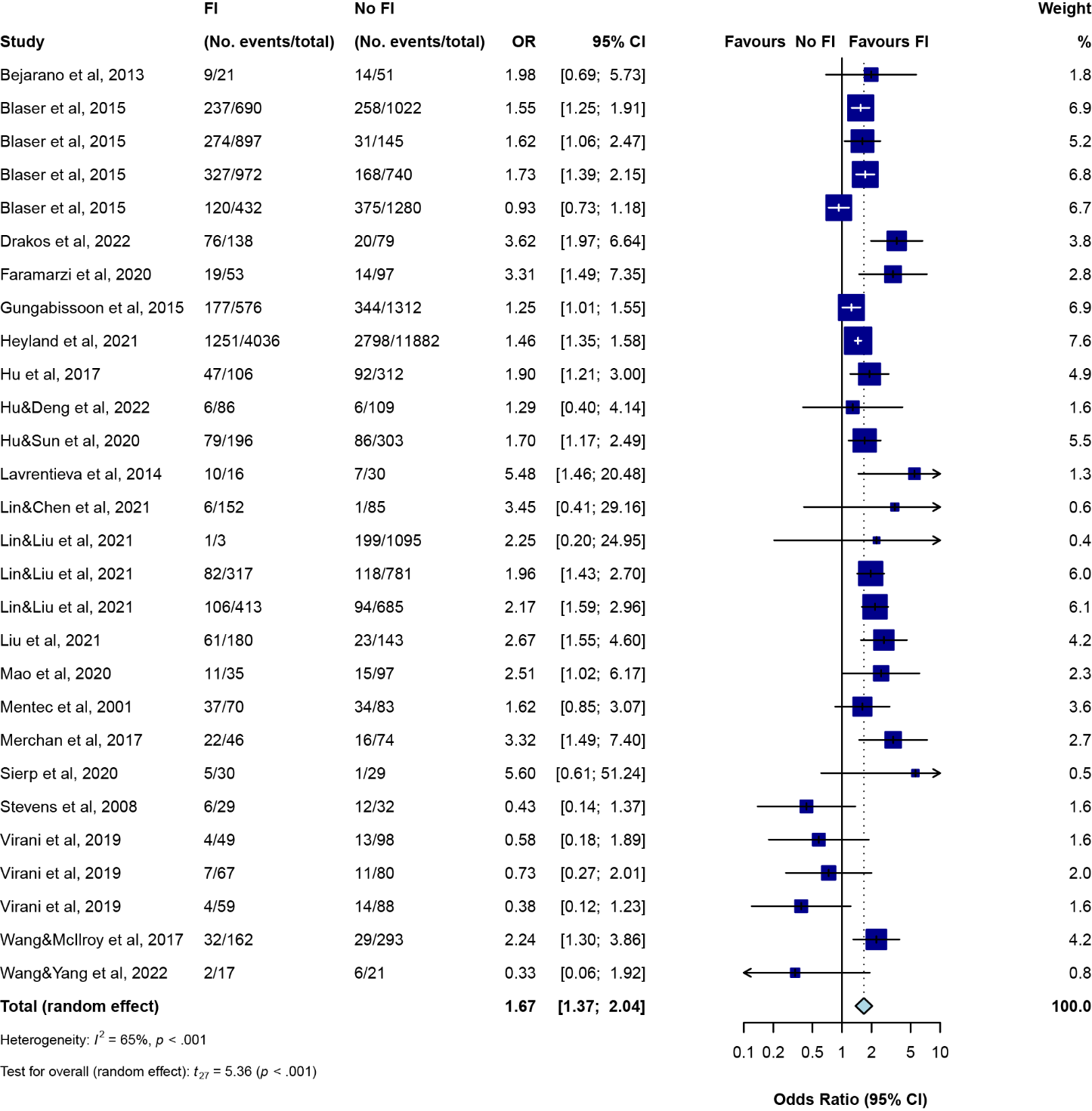


## 3.2 Association between FI and all-cause ICU mortality


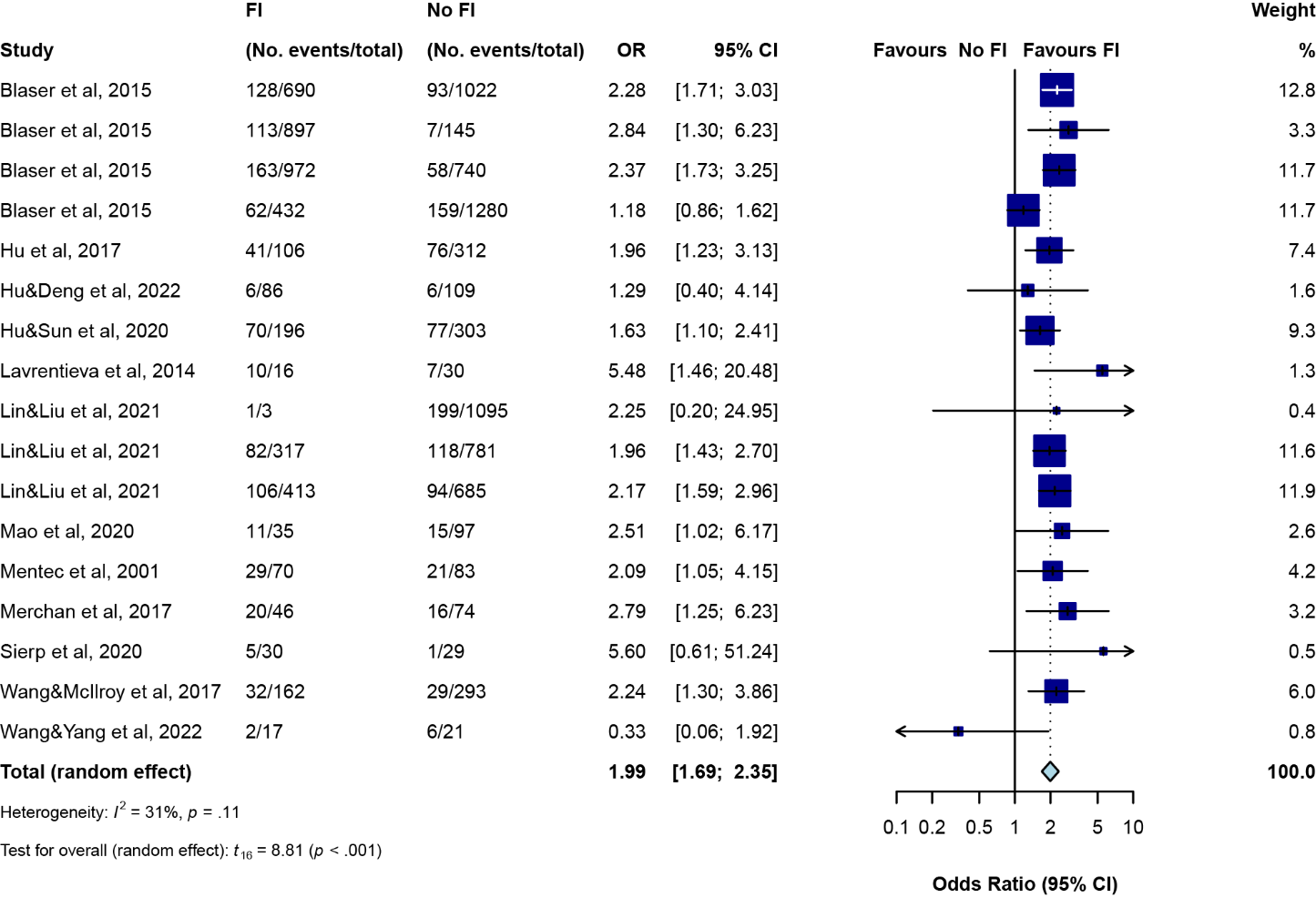


## 3.3 Association between FI and all-cause hospital mortality


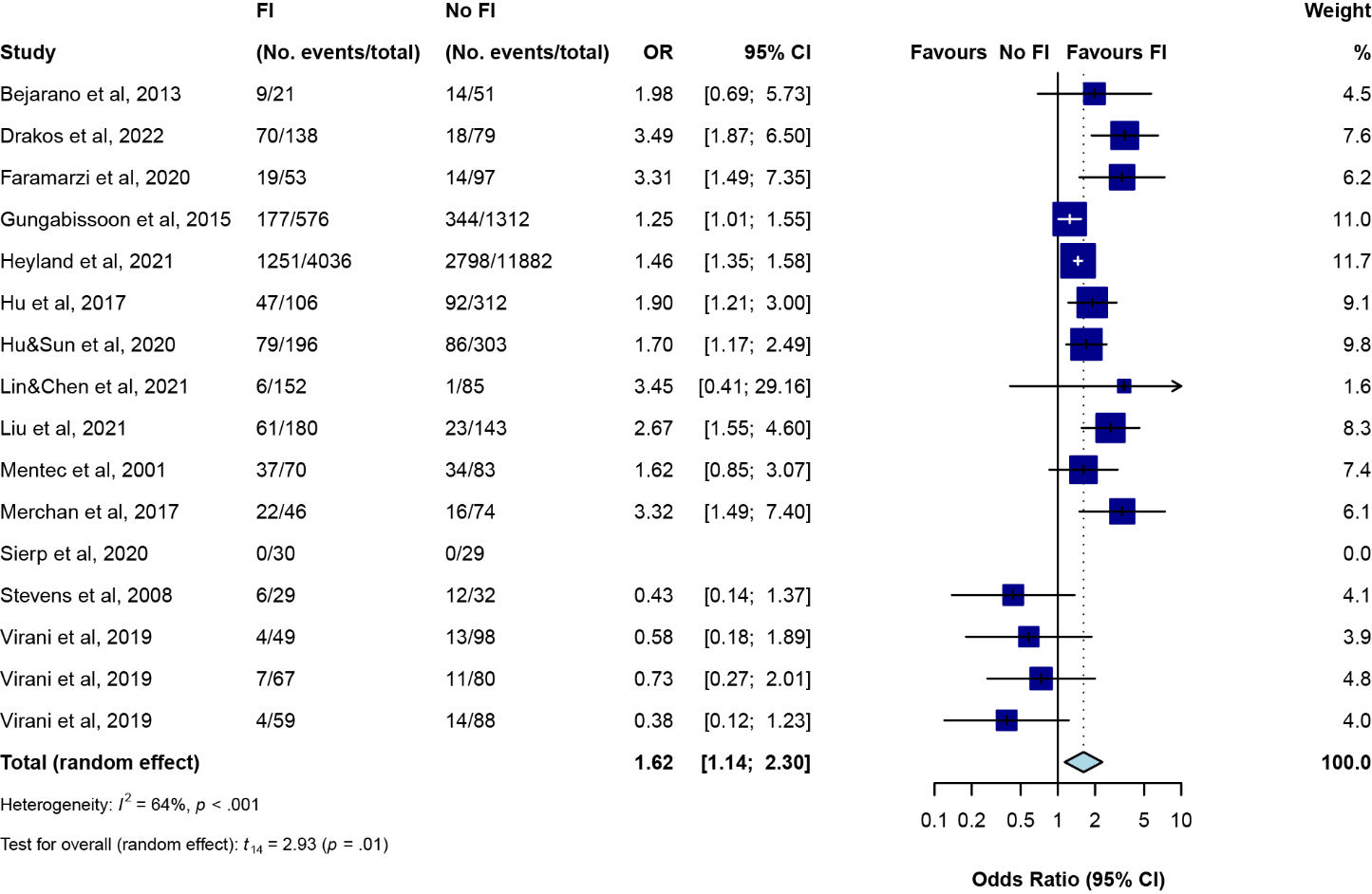


## 3.4 Association between FI and all-cause long-term mortality


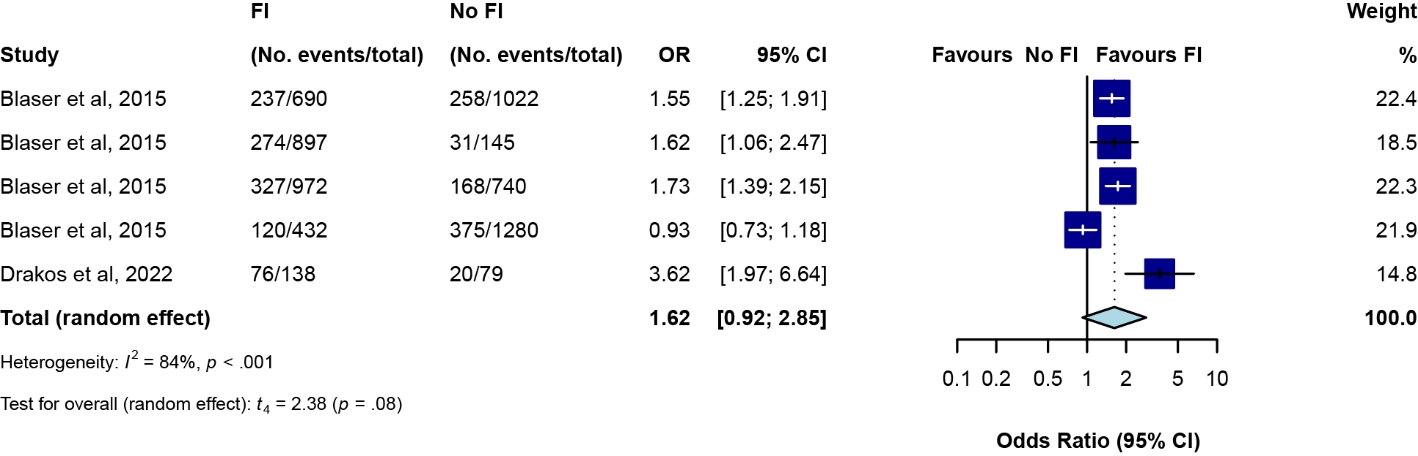


## 3.5 Association between FI and pneumonia


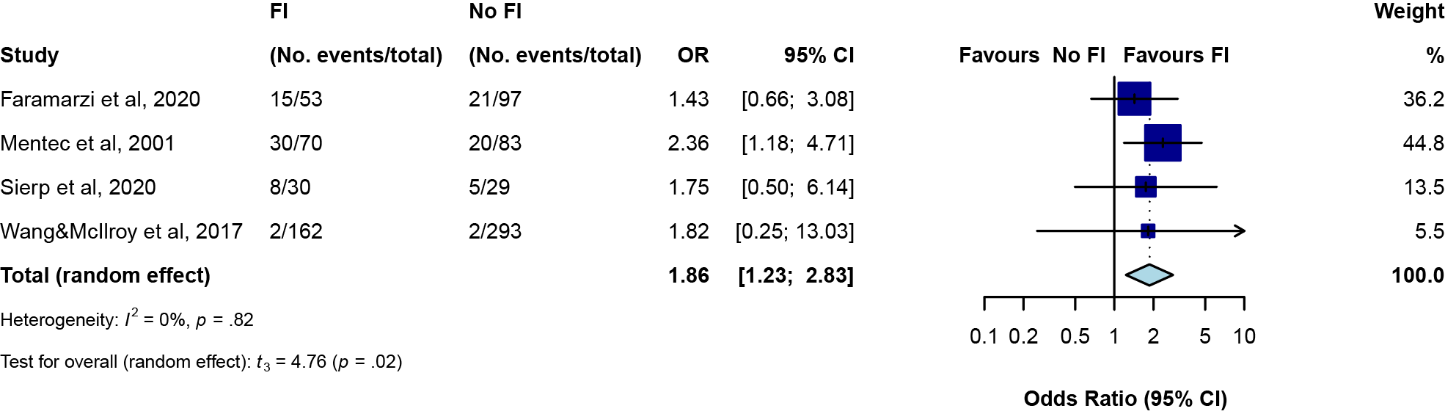


## 3.6 Association between FI and length of ICU stay


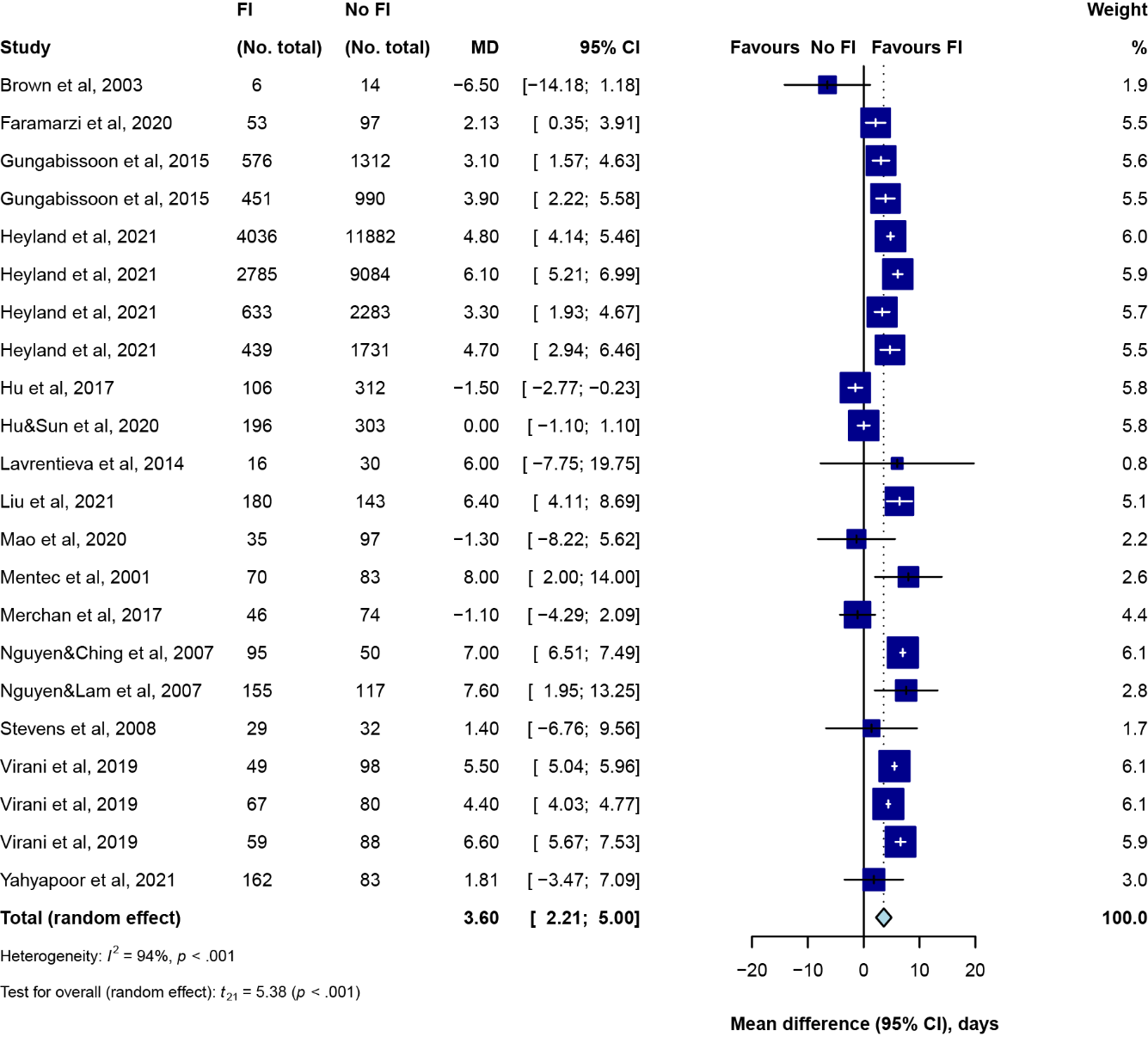


## 3.7 Association between FI and length of hospital stay


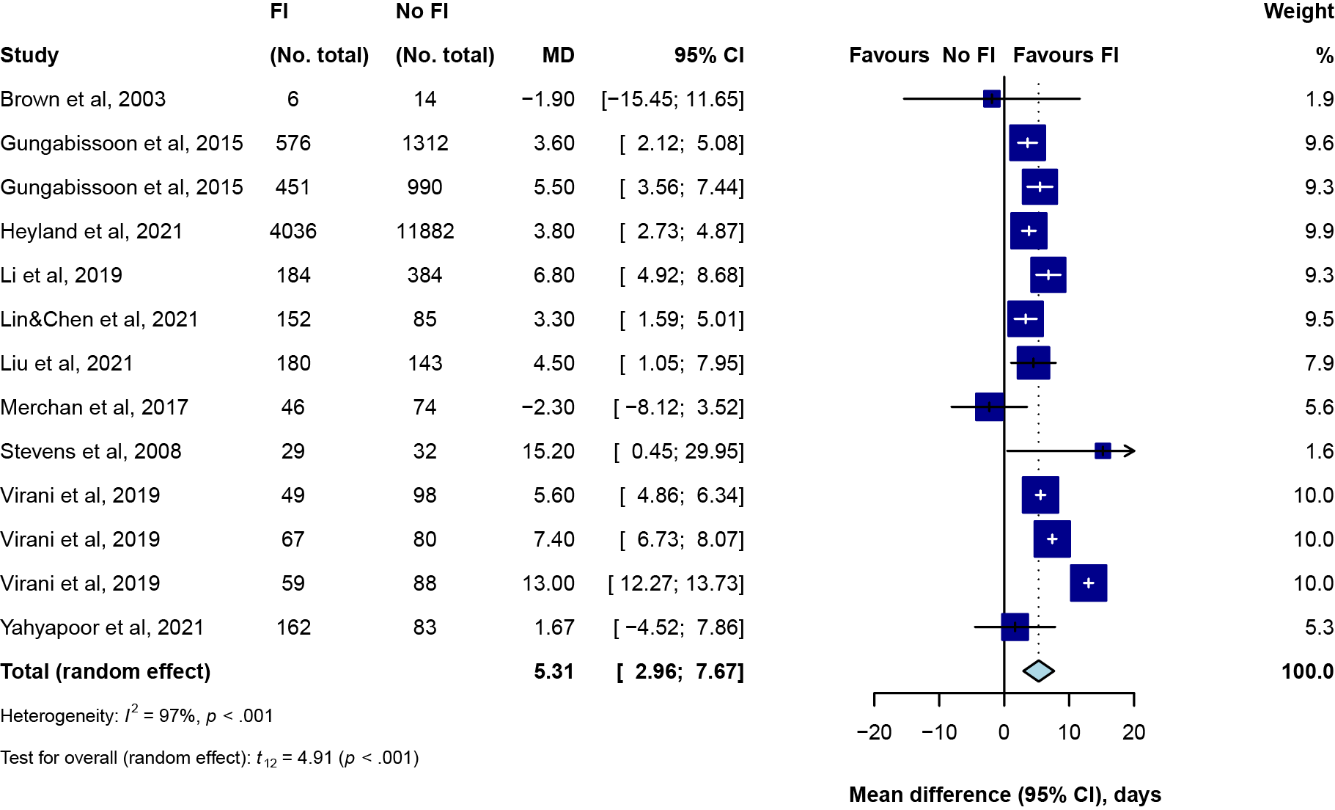


## 3.8 Association between FI and mechanical ventilation days


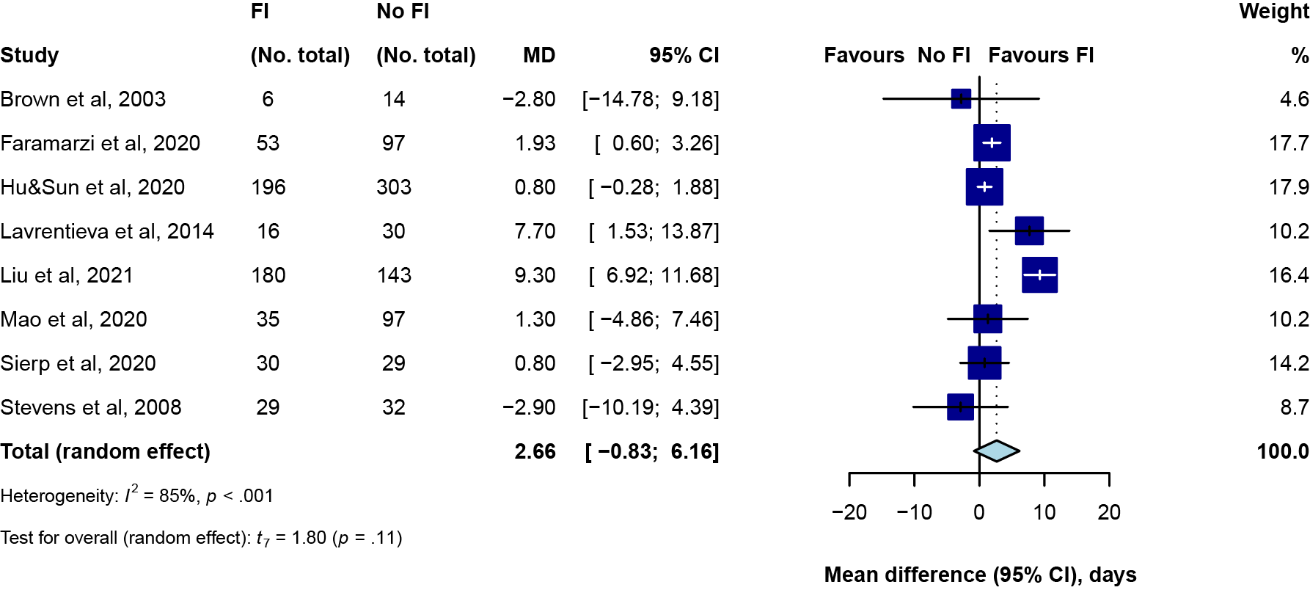


## 3.9 Association between FI and ICU mortality after redefining exposed cohort by defining FI according to GI symptoms cluster and EF insufficiency


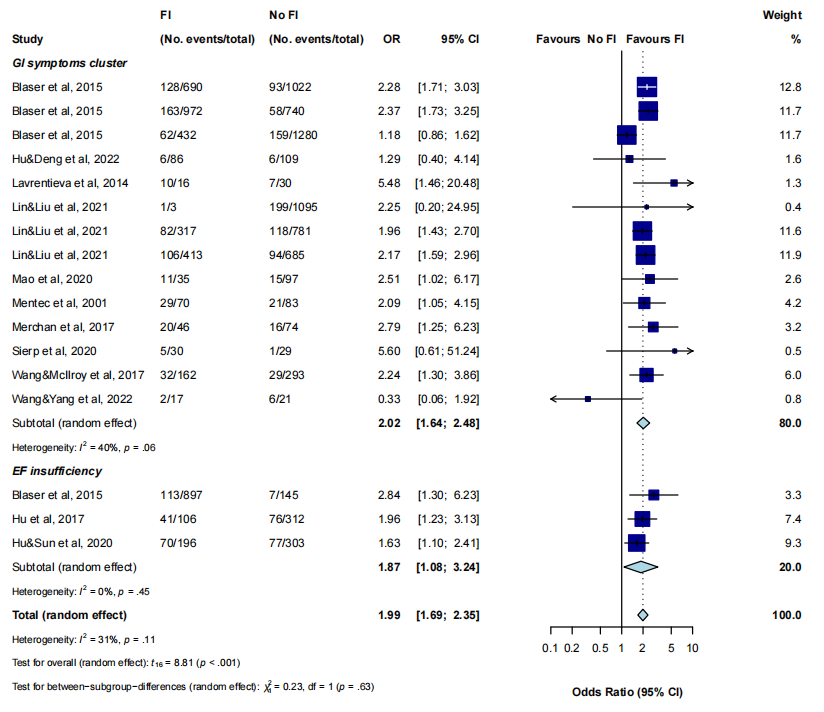


## 3.10 Association between FI and hospital mortality after redefining exposed cohort by defining FI according to GI symptoms cluster and EF insufficiency


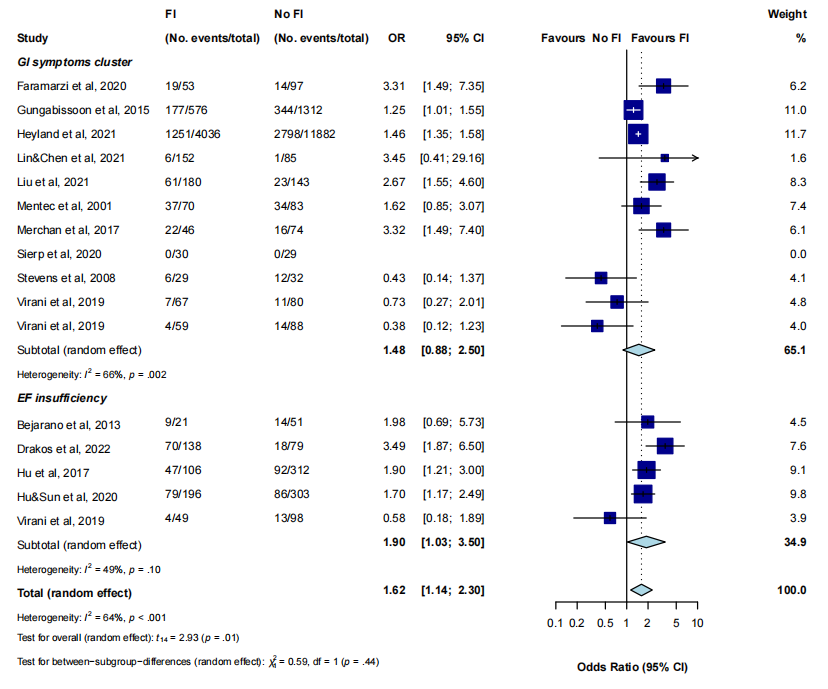


## 3.11 Association between FI and long-term mortality after redefining exposed cohort by defining FI according to GI symptoms cluster and EF insufficiency


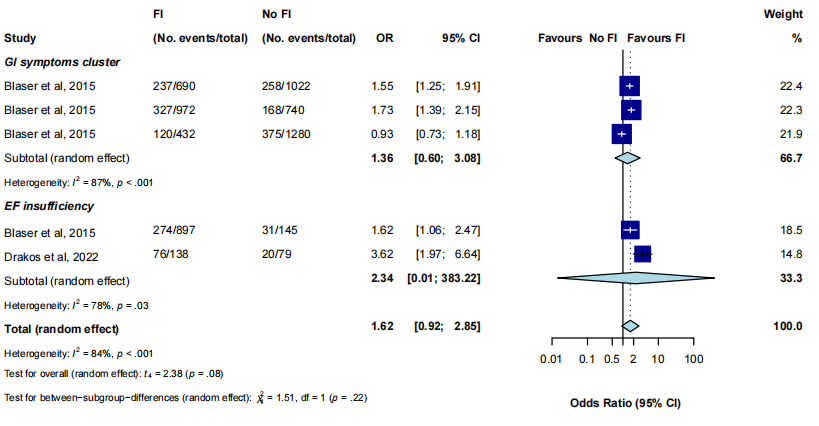

Supplement: Supplementary file 9 — Additional file 9. Fig S3 Forest plots of partial main results. [file 40560_2023_674_MOESM9_ESM.docx]
