# Supplementary material for: Different definitions of feeding intolerance and their associations with outcomes of critically ill adults receiving enteral nutrition: a systematic review and meta-analysis
Source: J Intensive Care. 2023 Jul 5;11:29. doi: 10.1186/s40560-023-00674-3 (PMC10320932; doi:10.1186/s40560-023-00674-3)
Supplement: Supplementary file 10 — Additional file 10. Table S7: FI pooled mortality/incidence of pneumonia and prevalence across different definitions [file 40560_2023_674_MOESM10_ESM.docx]

# Table S7: FI pooled mortality/incidence of pneumonia and prevalence across different definitions

| **Related Outcomes** | **FI types** | **FI definitions** | **Pooled mortality/ incidence of pneumonia, OR (95% CI)** | **Pooled prevalence of FI, % (95% CI)** |
| --- | --- | --- | --- | --- |
| All-cause hospital mortality | Overall | Any kind of definitions | 1.62 (1.14 to 2.3) | 41.68 (35.46 to 47.9) |
|  | Defining FI by GIS or EF insufficiency | Any GIS with large GRV | 1.42 (1.1 to 1.83) | 40.48 (29.41 to 51.56) |
|  |  | Any GIS without large GRV | 2.67 (1.55 to 4.6) | 55.73 (50.31 to 61.14) |
|  |  | EF insufficiency with any threshold | 1.9 (1.03 to 3.5) | 38.23 (24.88 to 51.58) |
|  |  | Large GRV with any threshold | 1.07 (0.08 to 15.2) | 43.75 (36.66 to 50.84) |
|  | Defining FI by No. of GIS | No. of GIS ≥ 1 | 1.44 (1.19 to 1.74) | 40.6 (27.15 to 54.05) |
|  |  | No. of GIS ≥ 2 | 0.38 (0.12 to 1.23) | 40.14 (32.21 to 48.06) |
|  | Defining FI by any GIS with large GRV | GIS+GRV ≥ 75±50 mL | 0.43 (0.14 to 1.37) | 47.54 (35.01 to 60.07) |
|  |  | GIS+GRV ≥ 250±50 mL | 1.9 (1.4 to 2.57) | 41.49 (31.61 to 51.38) |
|  |  | GIS+GRV ≥ 500±50 mL | 1.65 (0.27 to 9.98) | 43.5 (38.76 to 48.23) |
|  | Defining FI by large GRV | GRV ≥ 75±50 mL | 0.43 (0.14 to 1.37) | 47.54 (35.01 to 60.07) |
|  |  | GRV ≥ 250±50 mL | 3.31 (1.49 to 7.35) | 42.22 (27.11 to 57.32) |
|  |  | GRV ≥ 500±50 mL | 0.73 (0.27 to 2.01) | 45.58 (37.53 to 53.63) |
|  | Defining FI by EF insufficiency | EI percentage < 80% | 1.9 (1.03 to 3.5) | 38.23 (24.88 to 51.58) |
| All-cause long-term mortality | Overall | Any kind of definitions | 1.62 (0.92 to 2.85) | 54.37 (34.02 to 74.72) |
|  | Defining FI by GIS or EF insufficiency | Any GIS with large GRV | 1.73 (1.39 to 2.15) | 56.78 (54.43 to 59.12) |
|  |  | Any GIS without large GRV | 0.93 (0.73 to 1.18) | 25.23 (23.18 to 27.29) |
|  |  | EF insufficiency with any threshold | 2.34 (0.01 to 383.22) | 75.05 (53.02 to 97.09) |
|  |  | Large GRV with any threshold | 1.55 (1.25 to 1.91) | 40.3 (37.98 to 42.63) |
|  | Defining FI by No. of GIS | No. of GIS ≥ 1 | 1.73 (1.39 to 2.15) | 56.78 (54.43 to 59.12) |
|  |  | No. of GIS ≥ 2 | 1.83 (1.47 to 2.28) | 30.37 (28.2 to 32.55) |
|  |  | No. of GIS ≥ 3 | 2.8 (1.98 to 3.94) | 8.53 (7.21 to 9.85) |
|  | Defining FI by a specific symptom | Abdominal distention | 3.2 (1.95 to 5.24) | 3.91 (2.99 to 4.83) |
|  |  | Absent bowel sounds | 1.62 (1.27 to 2.06) | 21.61 (19.66 to 23.56) |
|  |  | Diarrhea | 3.19 (1.86 to 5.45) | 3.27 (2.43 to 4.11) |
|  |  | Vomiting | 0.93 (0.73 to 1.18) | 25.23 (23.18 to 27.29) |
|  | Defining FI by any GIS with large GRV | GIS+GRV ≥ 250±50 mL | 2.25 (0.01 to 473.08) | 51.75 (28.93 to 74.57) |
|  |  | GIS+GRV ≥ 500±50 mL | 1.67 (1.02 to 2.72) | 39.51 (5.68 to 73.34) |
|  |  | GIS+GRV ≥ 1000 mL | 1.96 (1.36 to 2.83) | 7.48 (6.23 to 8.72) |
|  | Defining FI by large GRV | GRV ≥ 250±50 mL | 1.55 (1.25 to 1.91) | 40.3 (37.98 to 42.63) |
|  |  | GRV ≥ 500±50 mL | 1.6 (1.25 to 2.03) | 22.25 (20.28 to 24.23) |
|  |  | GRV ≥ 1000 mL | 1.96 (1.36 to 2.83) | 7.48 (6.23 to 8.72) |
|  | Defining FI by EF insufficiency | EI percentage < 20% | 2.94 (2.33 to 3.71) | 57.77 (55.43 to 60.11) |
|  |  | EI percentage < 50% | 2.49 (1.74 to 3.57) | 85.22 (83.54 to 86.9) |
|  |  | EI percentage < 80% | 2.34 (0.01 to 383.22) | 75.05 (53.02 to 97.09) |
| All-cause ICU mortality | Overall | Any kind of definitions | 2 (1.69 to 2.35) | 38.74 (30.12 to 47.37) |
|  | Defining FI by GIS or EF insufficiency | Any GIS with large GRV | 2.29 (1.98 to 2.65) | 40.36 (33.72 to 46.99) |
|  |  | Any GIS without large GRV | 1.33 (0.29 to 5.99) | 29.54 (22.35 to 36.74) |
|  |  | EF insufficiency with any threshold | 1.87 (1.08 to 3.24) | 50.28 (14.24 to 86.32) |
|  |  | Large GRV with any threshold | 2.31 (1.63 to 3.27) | 30.07 (-0.21 to 60.36) |
|  | Defining FI by No. of GIS | No. of GIS ≥ 1 | 2.29 (1.98 to 2.65) | 40.36 (33.72 to 46.99) |
|  |  | No. of GIS ≥ 2 | 2.61 (1.07 to 6.37) | 19.87 (-0.71 to 40.44) |
|  |  | No. of GIS ≥ 3 | 4.49 (1.87 to 10.83) | 4.56 (-3.17 to 12.29) |
|  | Defining FI by a specific symptom | Abdominal distention | 2.86 (0.02 to 481.07) | 16.36 (-8.1 to 40.82) |
|  |  | Absent bowel sounds | 1.55 (0 to 1160.32) | 12.52 (-5.27 to 30.31) |
|  |  | Diarrhea | 2.68 (0.65 to 11.02) | 6.28 (0.29 to 12.26) |
|  |  | Vomiting | 1.49 (0.04 to 52.55) | 15.42 (-3.77 to 34.62) |
|  | Defining FI by any GIS with large GRV | GIS+GRV ≥ 250±50 mL | 2.07 (1.71 to 2.51) | 35.63 (28.92 to 42.33) |
|  |  | GIS+GRV ≥ 500±50 mL | 2.52 (1.96 to 3.23) | 30.52 (16.29 to 44.75) |
|  |  | GIS+GRV ≥ 1000 mL | 3.09 (2.05 to 4.67) | 7.48 (6.23 to 8.72) |
|  | Defining FI by large GRV | GRV ≥ 250±50 mL | 2.31 (0.54 to 9.93) | 43.63 (34.03 to 53.24) |
|  |  | GRV ≥ 500±50 mL | 2.58 (2.08 to 3.19) | 11.24 (-10.3 to 32.78) |
|  |  | GRV ≥ 1000 mL | 3.09 (2.05 to 4.67) | 7.48 (6.23 to 8.72) |
|  | Defining FI by EF insufficiency | EI percentage < 20% | 3.82 (2.68 to 5.47) | 57.77 (55.43 to 60.11) |
|  |  | EI percentage < 50% | 5.24 (2.55 to 10.74) | 85.22 (83.54 to 86.9) |
|  |  | EI percentage < 80% | 1.87 (1.08 to 3.24) | 50.28 (14.24 to 86.32) |
| All-cause mortality | Overall | Any kind of definitions | 1.67 (1.37 to 2.04) | 40.3 (34.17 to 46.43) |
|  | Defining FI by GIS or EF insufficiency | Any GIS with large GRV | 1.71 (1.32 to 2.21) | 40.01 (33.29 to 46.72) |
|  |  | Any GIS without large GRV | 1.41 (0.44 to 4.54) | 38.12 (23.62 to 52.61) |
|  |  | EF insufficiency with any threshold | 1.85 (1.21 to 2.82) | 46.3 (27.11 to 65.49) |
|  |  | Large GRV with any threshold | 1.45 (0.59 to 3.59) | 36.11 (20.75 to 51.48) |
|  | Defining FI by No. of GIS | No. of GIS ≥ 1 | 1.77 (1.42 to 2.2) | 39.99 (32.68 to 47.31) |
|  |  | No. of GIS ≥ 2 | 1.36 (0.15 to 12.59) | 26.35 (8.6 to 44.1) |
|  |  | No. of GIS ≥ 3 | 2.82 (1.64 to 4.85) | 4.56 (-3.17 to 12.29) |
|  | Defining FI by a specific symptom | Abdominal distention | 2.92 (1.04 to 8.17) | 12.19 (-4.07 to 28.45) |
|  |  | Absent bowel sounds | 1.69 (0.55 to 5.22) | 15.54 (3.65 to 27.42) |
|  |  | Diarrhea | 2.82 (1.94 to 4.1) | 5.24 (1.32 to 9.17) |
|  |  | Vomiting | 1.24 (0.47 to 3.26) | 18.68 (5.87 to 31.5) |
|  | Defining FI by any GIS with large GRV | GIS+GRV ≥ 75±50 mL | 0.43 (0.14 to 1.37) | 47.54 (35.01 to 60.07) |
|  |  | GIS+GRV ≥ 250±50 mL | 1.89 (1.51 to 2.36) | 39.78 (31.95 to 47.6) |
|  |  | GIS+GRV ≥ 500±50 mL | 1.71 (1.15 to 2.54) | 32.64 (19.83 to 45.46) |
|  |  | GIS+GRV ≥ 1000 mL | 1.96 (1.36 to 2.83) | 7.48 (6.23 to 8.72) |
|  | Defining FI by large GRV | GRV ≥ 75±50 mL | 0.43 (0.14 to 1.37) | 47.54 (35.01 to 60.07) |
|  |  | GRV ≥ 250±50 mL | 2.23 (0.57 to 8.75) | 40.3 (35.39 to 45.21) |
|  |  | GRV ≥ 500±50 mL | 1.37 (0.49 to 3.84) | 22.45 (-3.05 to 47.96) |
|  |  | GRV ≥ 1000 mL | 1.96 (1.36 to 2.83) | 7.48 (6.23 to 8.72) |
|  | Defining FI by EF insufficiency | EI percentage < 20% | 2.94 (2.33 to 3.71) | 57.77 (55.43 to 60.11) |
|  |  | EI percentage < 50% | 2.49 (1.74 to 3.57) | 85.22 (83.54 to 86.9) |
|  |  | EI percentage < 80% | 1.85 (1.21 to 2.82) | 46.3 (27.11 to 65.49) |
| The incidence of pneumonia | Overall | Any kind of definitions | 1.86 (1.23 to 2.83) | 40.59 (33.73 to 47.44) |
|  | Defining FI by GIS or EF insufficiency | Any GIS with large GRV | 2.3 (0.81 to 6.48) | 40.13 (30.24 to 50.01) |
|  |  | Large GRV with any threshold | 1.51 (0.49 to 4.68) | 42.22 (27.11 to 57.32) |
|  | Defining FI by any GIS with large GRV | GIS+GRV ≥ 250±50 mL | 1.54 (1.13 to 2.09) | 38.82 (30.99 to 46.64) |
|  |  | GIS+GRV ≥ 500±50 mL | 2.36 (1.18 to 4.71) | 45.75 (37.86 to 53.65) |

FI=feeding intolerance, GISs=gastrointestinal symptoms, GRV=Gastric residual volume, EI=EF, ICU=intensive care unit, No.=number, CI= confidence interval.
