# Supplementary material for: Different definitions of feeding intolerance and their associations with outcomes of critically ill adults receiving enteral nutrition: a systematic review and meta-analysis
Source: J Intensive Care. 2023 Jul 5;11:29. doi: 10.1186/s40560-023-00674-3 (PMC10320932; doi:10.1186/s40560-023-00674-3)
Supplement: Supplementary file 11 — Additional file 11. Fig S4: Data summary of relative effects for all-cause mortality, all-cause ICU mortality and length of ICU stay overall and by different FI definitions. [file 40560_2023_674_MOESM11_ESM.docx]

# Fig S4: Data summary of relative effects for all-cause mortality, all-cause ICU mortality and length of ICU stay overall and by different FI definitions


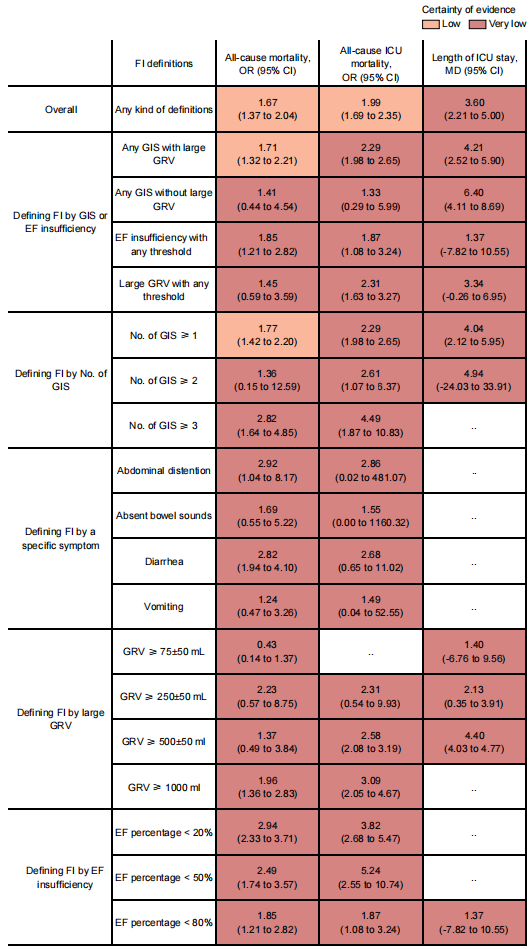


The color of each cell indicates the certainty of evidence according to the Grading of Recommendations Assessment, Development, and Evaluation. FI=feeding intolerance, GISs=gastrointestinal symptoms, GRV=Gastric residual volume, EF=enteral feeding, ICU= intensive care unit, No.=number, OR=odds ratio, CI= confidence interval.
