# Supplementary material for: Different definitions of feeding intolerance and their associations with outcomes of critically ill adults receiving enteral nutrition: a systematic review and meta-analysis
Source: J Intensive Care. 2023 Jul 5;11:29. doi: 10.1186/s40560-023-00674-3 (PMC10320932; doi:10.1186/s40560-023-00674-3)
Supplement: Supplementary file 12 — Additional file 12. Fig S5: Subgroup analyses for all-cause mortality, all-cause ICU mortality and length of ICU stay according to FI definition-related key elements. [file 40560_2023_674_MOESM12_ESM.docx]

# Fig S5: Subgroup analyses for all-cause mortality, all-cause ICU mortality and length of ICU stay according to FI definition-related key elements


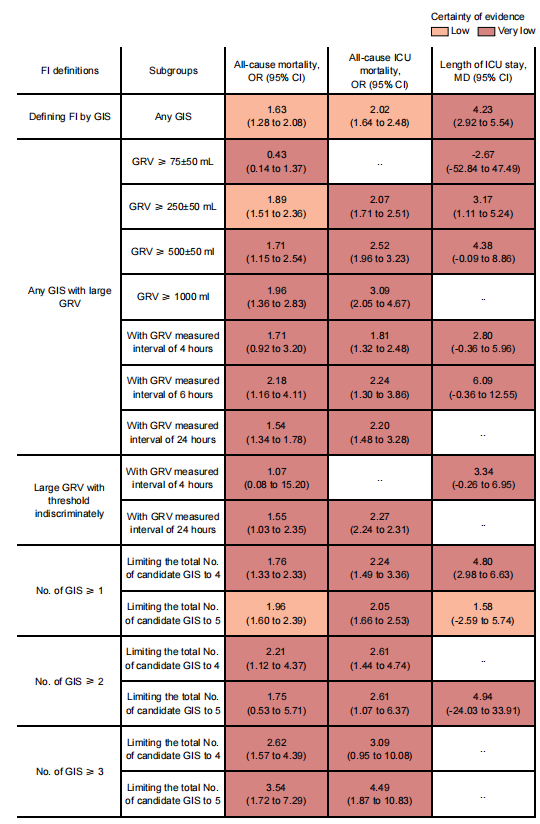


The certainty of the evidence was rated by the Grading of Recommendations Assessment, Development, and Evaluation criteria. GISs here no matter in candidate GISs or selected GISs to determine FI at least included large GRV. GISs are referred to as large GRV alone or large GRV combined with another one or any combination of symptoms including vomiting, absent bowel sounds, abdominal distension, and diarrhea. FI=feeding intolerance, GISs=gastrointestinal symptoms, GRV=Gastric residual volume, ICU= intensive care unit, No.=number, OR=odds ratio, CI= confidence interval.
