# Supplementary material for: Different definitions of feeding intolerance and their associations with outcomes of critically ill adults receiving enteral nutrition: a systematic review and meta-analysis
Source: J Intensive Care. 2023 Jul 5;11:29. doi: 10.1186/s40560-023-00674-3 (PMC10320932; doi:10.1186/s40560-023-00674-3)
Supplement: Supplementary file 13 — Additional file 13. Fig S6: Subgroup analyses for all-cause mortality and all-cause ICU mortality, as well as the length of ICU stay according to characteristic levels of the study patients regardless of the kinds of FI definitions. [file 40560_2023_674_MOESM13_ESM.docx]

# Fig S6: Subgroup analyses for all-cause mortality and all-cause ICU mortality, as well as the length of ICU stay according to characteristic levels of the study patients regardless of the kinds of FI definitions


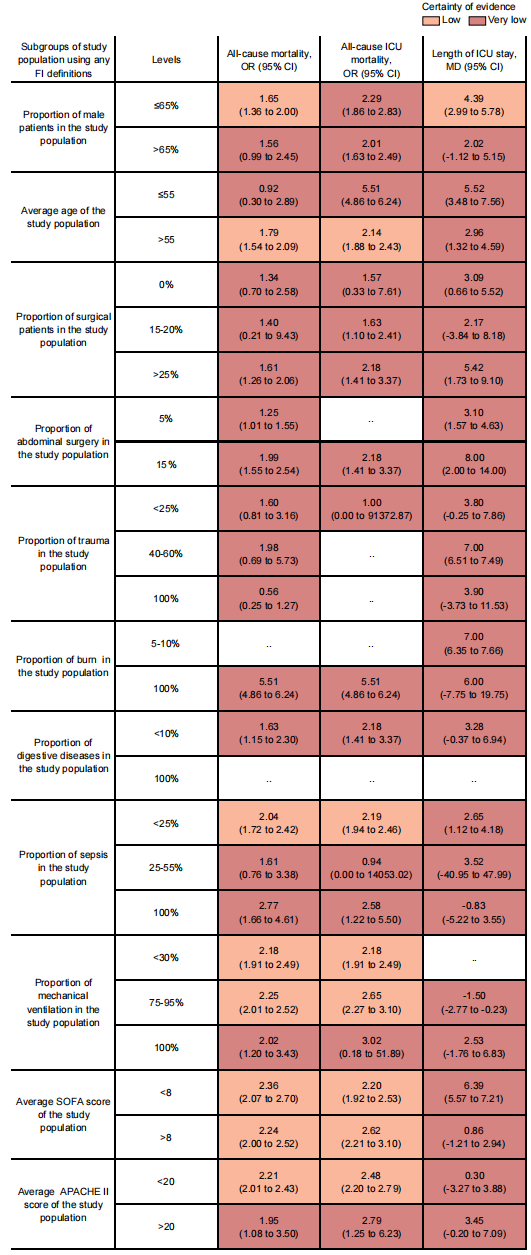


The certainty of the evidence was rated by the Grading of Recommendations Assessment, Development, and Evaluation criteria. Data were expressed as ORs and MDs along with their 95% CIs. FI=feeding intolerance, ICU=intensive care unit, SOFA=Sequential Organ Failure Assessment, APACHE II=Acute Physiology, and Chronic Health Evaluation II, OR=odds ratio, MD=mean difference, CI=confidence interval.
