# Supplementary material for: Different definitions of feeding intolerance and their associations with outcomes of critically ill adults receiving enteral nutrition: a systematic review and meta-analysis
Source: J Intensive Care. 2023 Jul 5;11:29. doi: 10.1186/s40560-023-00674-3 (PMC10320932; doi:10.1186/s40560-023-00674-3)
Supplement: Supplementary file 14 — Additional file 14. Fig S7: Data summary of absolute effects on all-cause mortality and all-cause ICU mortality overall and by FI definitions. [file 40560_2023_674_MOESM14_ESM.docx]

# Fig S7: Data summary of absolute effects on all-cause mortality and all-cause ICU mortality overall and by FI definitions


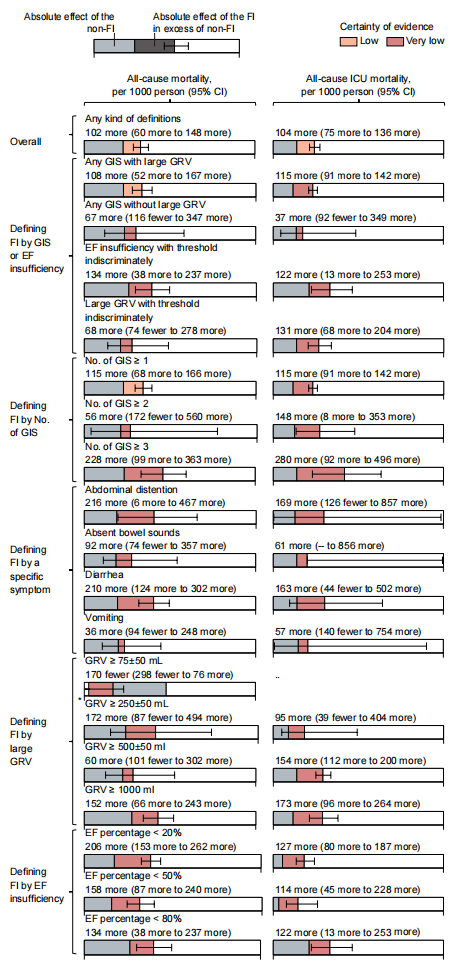


Effects of FI and non-FI changed together in pairs with the different definitions of FI. Absolute effects of the FI in excess of non-FI were estimated overall or across 18 different FI definitions via a random effect meta-analysis of rates. This pooled effect represents how many more patients with poor outcomes can expect to occur due to FI. *Axis starting point is -200 per 1000 events. FI=feeding intolerance, GISs=gastrointestinal symptoms, GRV=Gastric residual volume, EF=enteral feeding, ICU=intensive care unit, No.=number, CI= confidence interval.
