# Supplementary material for: Different definitions of feeding intolerance and their associations with outcomes of critically ill adults receiving enteral nutrition: a systematic review and meta-analysis
Source: J Intensive Care. 2023 Jul 5;11:29. doi: 10.1186/s40560-023-00674-3 (PMC10320932; doi:10.1186/s40560-023-00674-3)
Supplement: Supplementary file 15 — Additional file 15. Fig S8: Two-dimensional graphs of FI prevalence versus all-cause mortality and all-cause ICU mortality by different FI definitions. [file 40560_2023_674_MOESM15_ESM.docx]

# Fig S8: Two-dimensional graphs of FI prevalence versus all-cause mortality and all-cause ICU mortality by different FI definitions


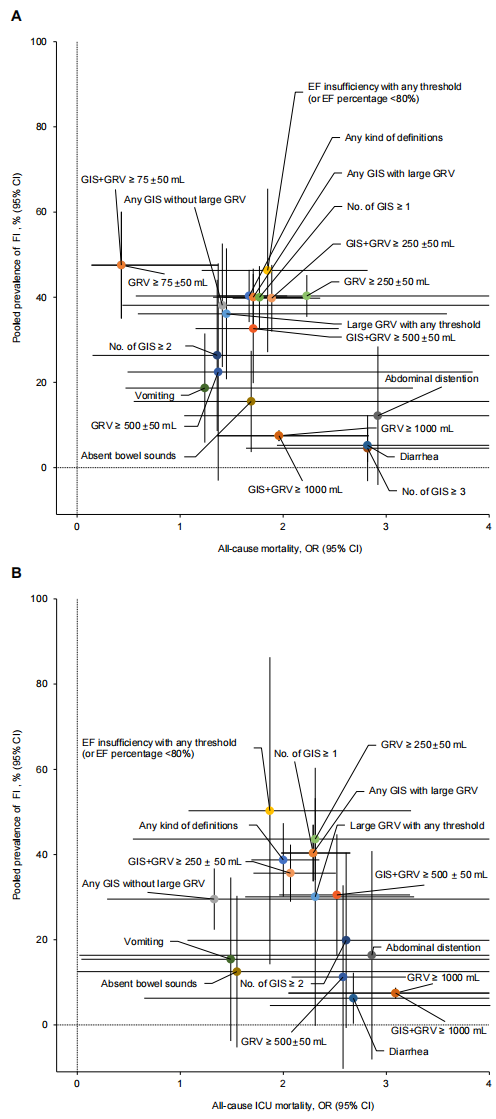


(A) All-cause mortality versus FI prevalence. (B) All-cause ICU mortality versus ICU FI prevalence. Effect sizes for FI by different definitions are represented by colored nodes, with bars representing the corresponding 95% CIs. FI prevalence by EF percentage <20% or <50% was not pooled due to their cohorts being completely covered by the same cohort with EF percentage <80% in our study, if not, significant selectivity errors were expected. FI=feeding intolerance, EF=enteral feeding, GRV=Gastric residual volume, GISs=gastrointestinal symptoms, ICU=intensive care unit, No.=number, OR=odds ratio, CI= confidence interval.
