# Supplementary material for: Different definitions of feeding intolerance and their associations with outcomes of critically ill adults receiving enteral nutrition: a systematic review and meta-analysis
Source: J Intensive Care. 2023 Jul 5;11:29. doi: 10.1186/s40560-023-00674-3 (PMC10320932; doi:10.1186/s40560-023-00674-3)

# Fig S9: Forest plots of sensitivity analysis

## 9.1 Sensitivity analysis for the association between FI and all-cause mortality by removal of the only study written in Chinese


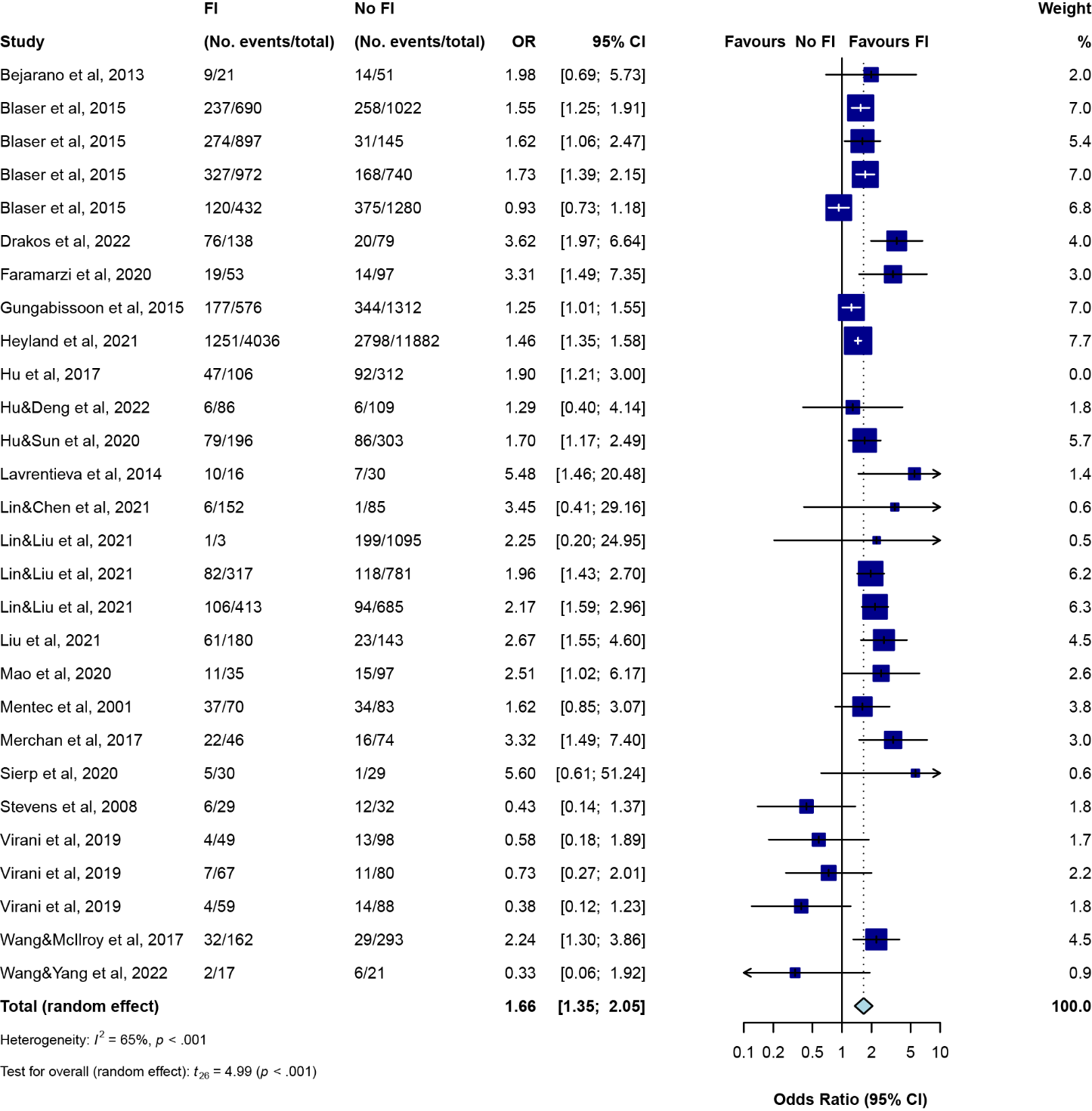


## 9.2 Sensitivity analysis for the association between FI and all-cause ICU mortality by removal of the only study written in Chinese


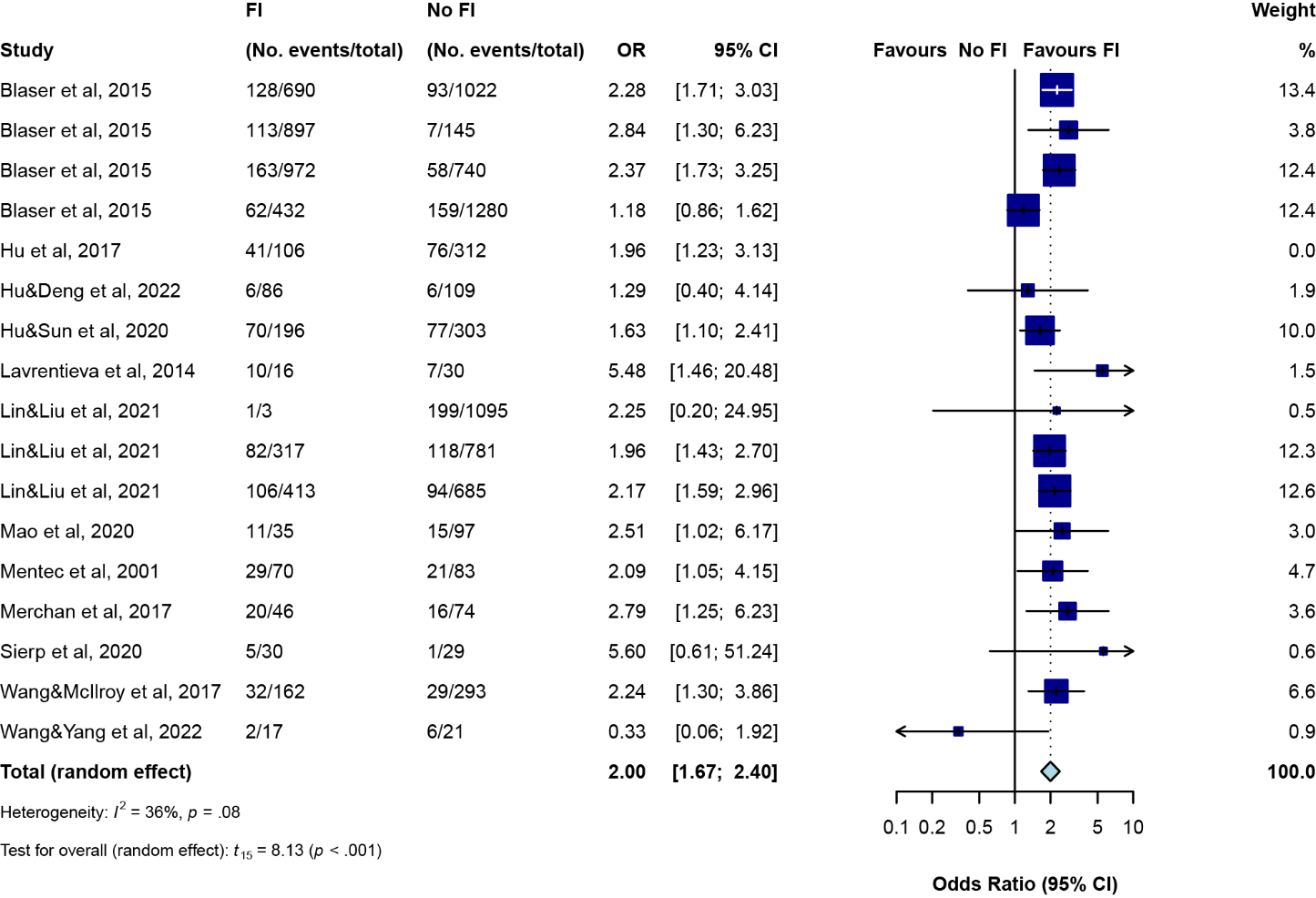


## 9.3 Sensitivity analysis for the association between FI and all-cause hospital mortality by removal of the only study written in Chinese


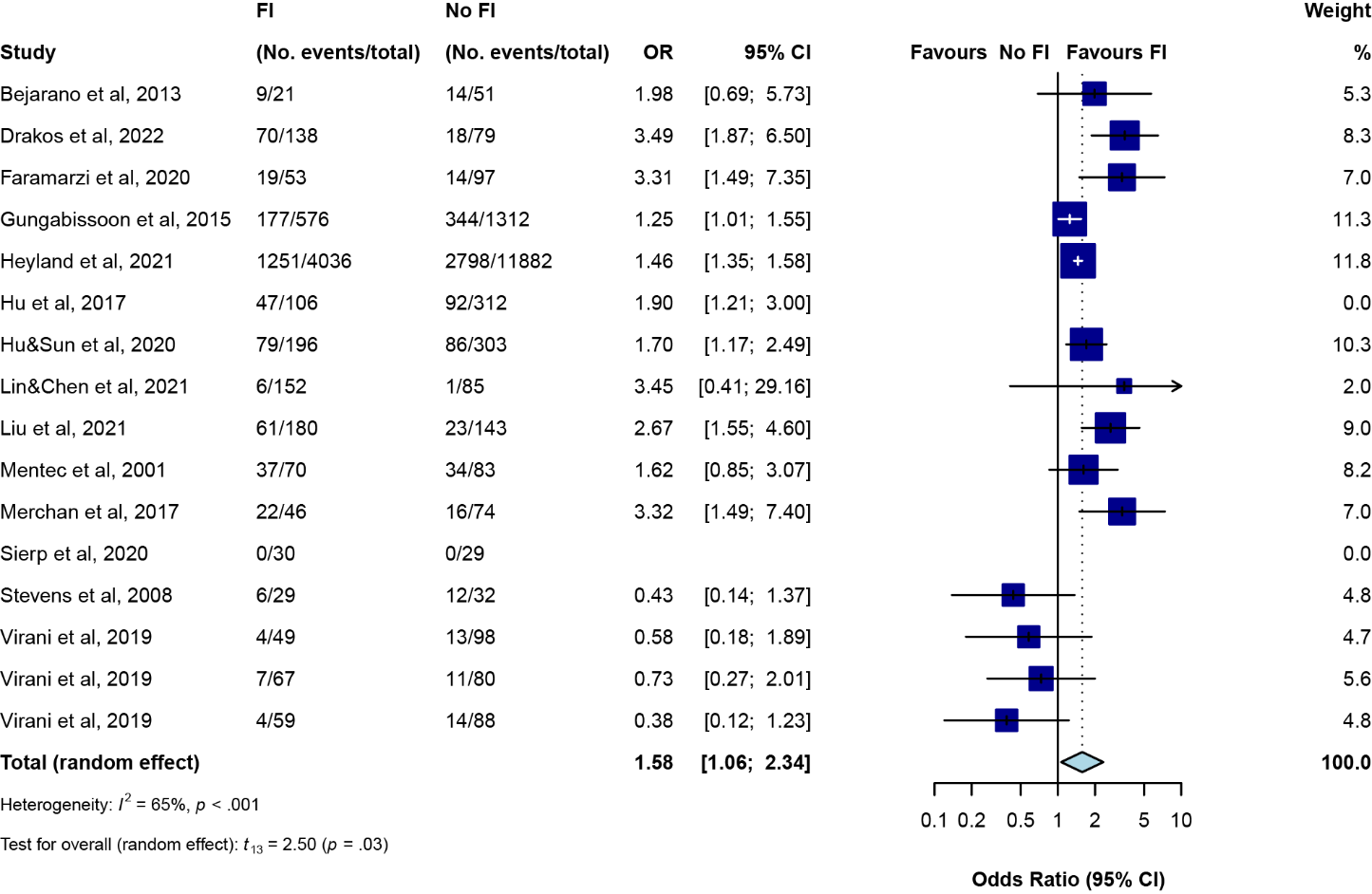


## 9.4 Sensitivity analysis for the association between FI and length of ICU stay by removal of the only study written in Chinese


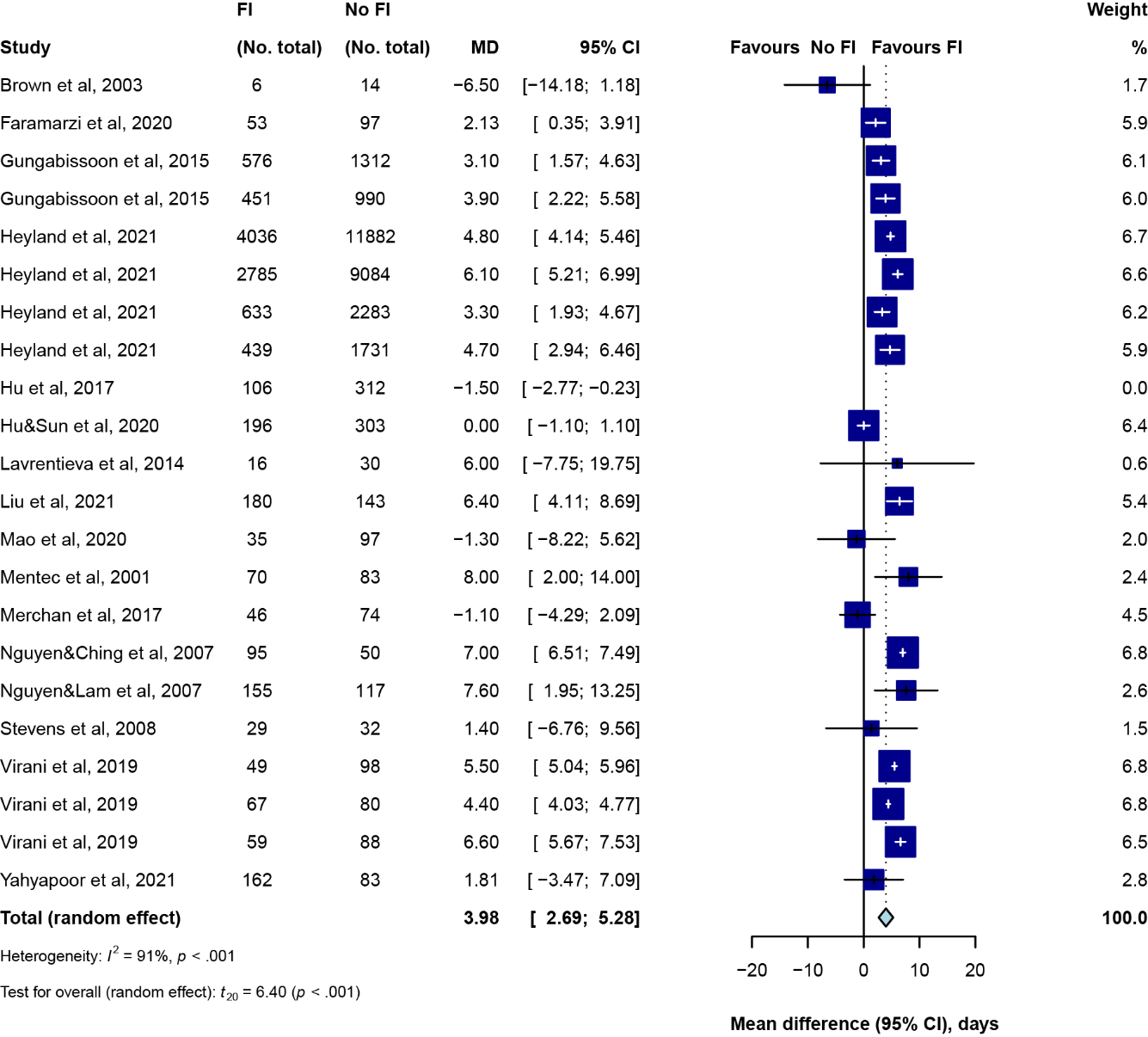


## 9.5 Sensitivity analysis for the association between FI and all-cause mortality after redefining exposed cohort by defining FI according to GI symptoms cluster and EF insufficiency by removal of the only study written in Chinese


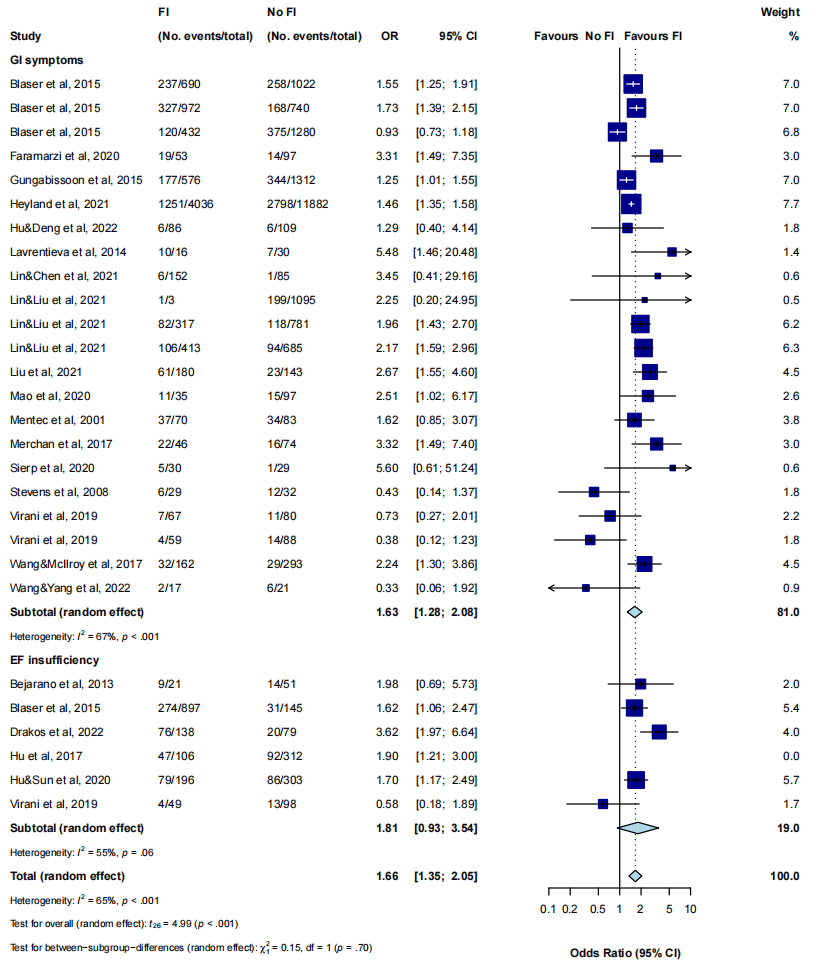


## 9.6 Sensitivity analysis for the association between FI and all-cause ICU mortality after redefining exposed cohort by defining FI according to GI symptoms cluster and EF insufficiency by removal of the only study written in Chinese


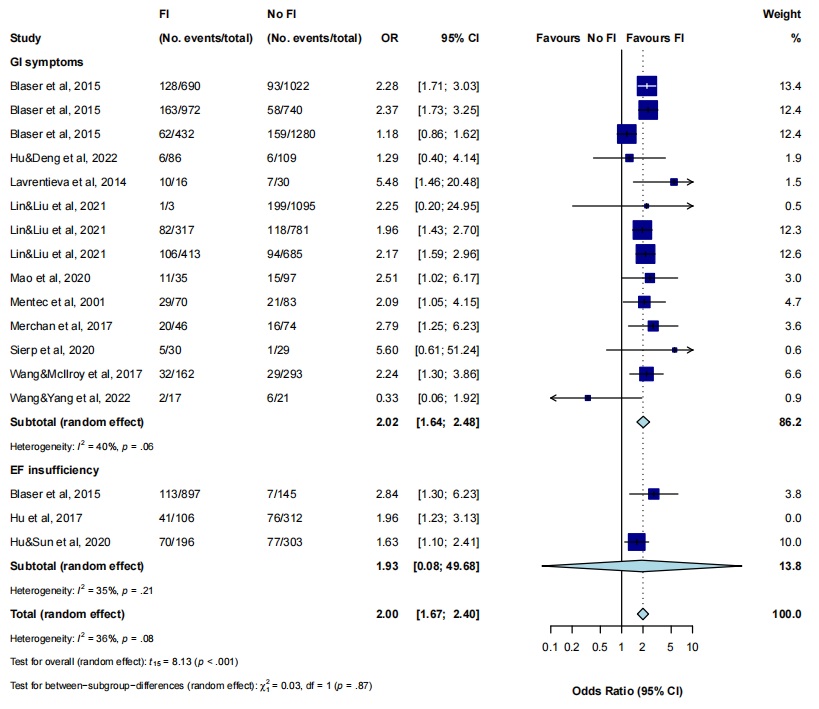


## 9.7 Sensitivity analysis for the association between FI and all-cause hospital mortality after redefining exposed cohort by defining FI according to GI symptoms cluster and EF insufficiency by removal of the only study written in Chinese


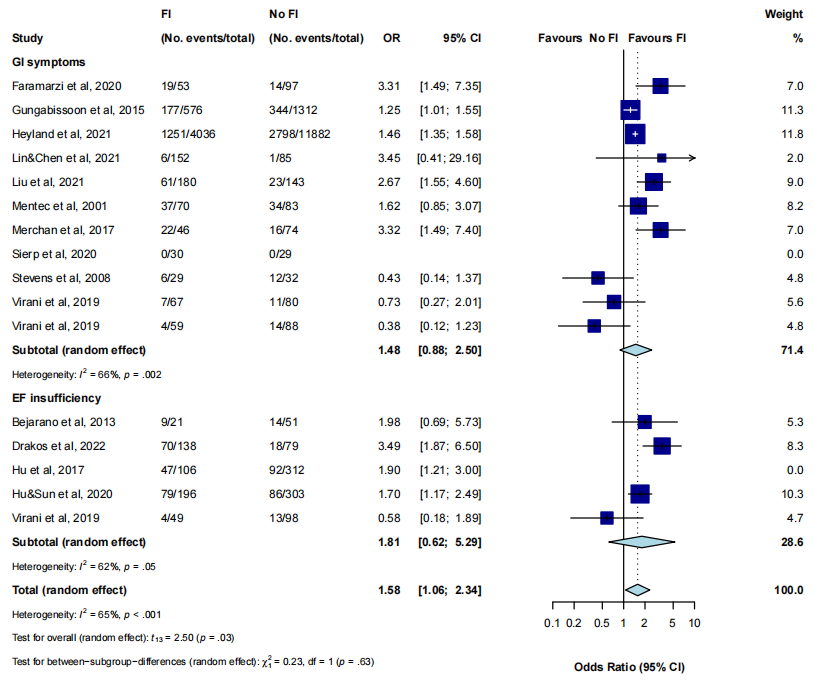


## 9.8 Sensitivity analysis for the association between FI and length of ICU stay after redefining exposed cohort by defining FI according to GI symptoms cluster and EF insufficiency by removal of the only study written in Chinese


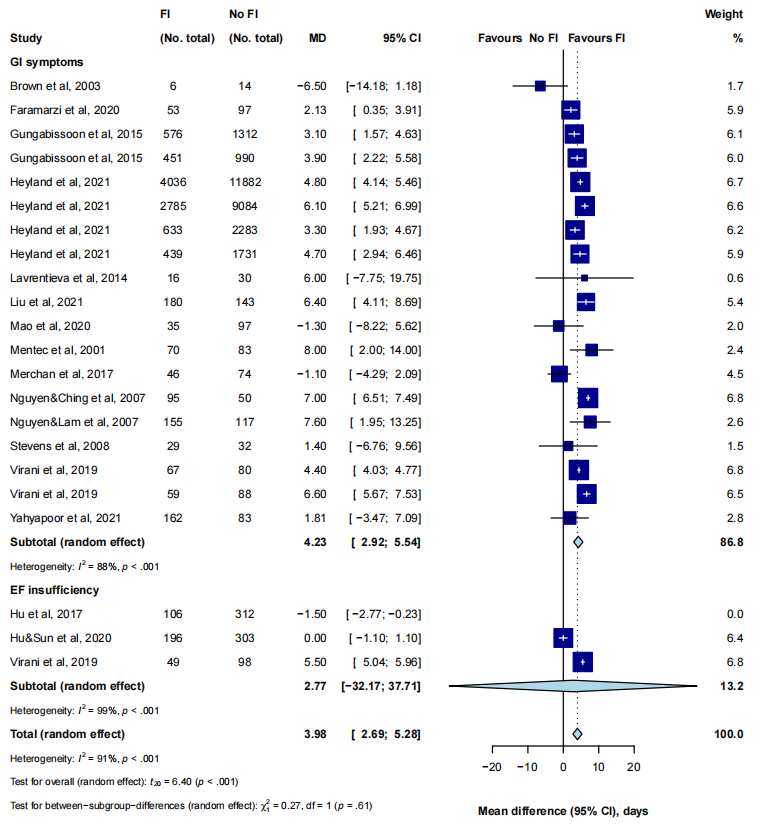

Supplement: Supplementary file 16 — Additional file 16. Fig S9: Forest plots of sensitivity analysis. [file 40560_2023_674_MOESM16_ESM.docx]
